# First global synthesis of heat-related mental health impacts in children and young people: a systematic review and meta-analysis

**DOI:** 10.1093/ije/dyag121

**Published:** 2026-07-25

**Authors:** Shevonne Kwan, Jacqueline Stephens, Corey J A Bradshaw, Blesson M Varghese, Kirrilly Thompson, Melinda A Judge, Peter N Le Souëf, Syeda H Fatima

**Affiliations:** College of Medicine and Public Health, Flinders Health and Medical Research Institute, Flinders University, Adelaide, South Australia, Australia; College of Medicine and Public Health, Flinders Health and Medical Research Institute, Flinders University, Adelaide, South Australia, Australia; Global Ecology | Partuyarta Ngadluku Wardli Kuu, College of Science and Engineering, Flinders University, Adelaide, South Australia, Australia; School of Public Health, College of Health, Adelaide University, Adelaide, South Australia, Australia; College of Medicine and Public Health, Flinders Health and Medical Research Institute, Flinders University, Adelaide, South Australia, Australia; National Centre for Education and Training on Addiction, Flinders University, Adelaide, South Australia, Australia; School of Medicine and Public Health, The University of Newcastle, Callaghan, New South Wales, Australia; Wal-Yan Respiratory Research Centre, The Kids Research Institute Australia, Perth, Western Australia, Australia; School of Mathematics and Statistics, University of Western Australia, Western Australia, Australia; School of Medicine, The University of Western Australia, Perth, Western Australia, Australia; College of Medicine and Public Health, Flinders Health and Medical Research Institute, Flinders University, Adelaide, South Australia, Australia; Global Ecology | Partuyarta Ngadluku Wardli Kuu, College of Science and Engineering, Flinders University, Adelaide, South Australia, Australia; HEAL Global Research Centre, Health Research Institute, University of Canberra, Canberra, Australian Capital Territory, Australia

**Keywords:** climate change, mental health, children and adolescents, heat exposure, high temperature, heatwaves, systematic review

## Abstract

**Background:**

Climate change is increasing the frequency and severity of non-optimal temperatures and heatwaves, posing risks to public health, especially for children and young people. We performed a systematic review and meta-analysis to assess the impacts of (i) non-optimal ambient temperatures and (ii) heatwaves on mental health outcomes in this group globally.

**Methods:**

We searched five databases for studies published between 2000 and 2024. Following the Preferred Reporting Items for Systematic Reviews and Meta-Analyses (PRISMA) guidelines, we selected studies by using the population–exposure–comparison–outcome (PECO) framework (PROSPERO protocol ID CRD42024524650). We performed a random-effects meta-analysis, assessed the risk of bias, and examined variation by age, gender, income, and climate zones.

**Results:**

Of 23 included studies, 19 examined ambient temperature and 4 examined heatwaves. A 1°C increase in daily ambient temperature was associated with a 0.9% increase in morbidity-related mental health outcomes across varied healthcare encounters and diagnoses (relative risk = 1.009; 95% confidence interval: 1.003–1.016). Children aged 5–18 years were most vulnerable, with a 1.5% increased risk associated with high temperatures (1.005–1.026) and 25% increased risk during heatwaves (1.006–1.561). Climate-zone analyses showed that children and young people in humid subtropical and hot-summer continental zones had the highest risks from high temperatures (1.004–1.036 and 1.004–1.038, respectively).

**Conclusion:**

High temperatures and heatwaves were associated with adverse mental health risks in children and young people. Data were lacking for some of the hottest and most socio-economically disadvantaged regions globally. Future research should prioritize these regions, explore mechanistic pathways, and generate disaggregated data.

Key Messages

- We examined the association between heat exposure and mental health outcomes in children and youth.
- We found a 0.9% higher risk of broad mental health outcomes associated with heat exposure, especially among those aged 5–18 years and in humid and hot-summer climates.
- These findings highlight the need for targeted heat–mental health protection for children, especially in high-risk and under-studied regions.

## Introduction

The mean global temperature has risen by >1.2°C since pre-industrial times [1], with 3.6 billion people living in regions that are highly vulnerable to climate change [2]. Climate change affects health through pathways including food insecurity, infectious-disease transmission, respiratory illness, heat-related illness and death, undernutrition, and mental health challenges [1, 2].

High temperatures can harm health through physiological stress responses such as dehydration and electrolyte imbalance, which may impair both physical and mental functioning and exacerbate symptoms including confusion, fatigue, and irritability [3]. Because the neurotransmitters involved in thermoregulation (e.g. serotonin, dopamine, and noradrenaline) also influence mood, heat-related disruptions may also contribute directly to mental health disorders [4].

In 2019, 293 million children and young people aged 5–24 years were living with one or more mental disorders, making mental illness a leading contributor to the global disease burden [5]. Rising temperatures have been associated with adverse mental health outcomes, including increased emergency visits, anxiety and depression, and suicide attempts [6–8]. Children and young people may be especially vulnerable because of their physiological and behavioural susceptibility to heat, including reduced heat-dissipation capacity, limited emotional regulation, and lower likelihood of adopting protective behaviours during extreme heat [9, 10].

Several global systematic reviews have established a strong, positive association between heat and adverse mental health outcomes [11–14] but most were focused on general populations or the elderly. Evidence for children and young people remains limited and inconclusive [15, 16]. Our objective was therefore to conduct a global systematic review and meta-analysis focused on the mental health impacts of heat in children and young people, to guide future policies, interventions, and climate-adaptation strategies.

## Methods

We performed a systematic review and meta-analysis by following the Preferred Reporting Items for Systematic Reviews and Meta-Analyses (PRISMA) [17] guidelines (PRISMA checklist: Supplementary Section A, Table A1). We registered the protocol with the PROSPERO International prospective register of systematic reviews (ID CRD42024524650). We present a systematic review that does not involve any original research with human participants or animals, so no ethical approval was required.

**Table 1:** Study eligibility criteria based on PECO elements.

**Participants**	Children and young people aged 0–25 years. ‘Children’ and ‘young people’ can refer to different age ranges across studies; in this review, we used these terms to refer to individuals aged 0–25 years [19, 20]
**Exposure**	As the authors of the original studies predefined non-optimal ambient temperatures and heatwave events, there is no universally applicable definition of ‘heatwave’ because the effects vary at subnational scales due to geography, topography, and other conditions [21, 22]; we therefore refer to heatwaves and non-optimal ambient temperatures defined by the region in question
**Comparator**	For high-temperature studies, we estimated risks relative to reference temperatures, thresholds, or percentiles associated with lower risk; for heatwave studies, we compared the effect estimates to non-heatwave periods
**Outcome**	Morbidity-related mental health outcomes. ‘Mental health outcomes’ include morbidity-related mental disorders and psychosocial disabilities as defined by the International Classification of Diseases (Supplementary Section B, Table B2) and ‘mental states’ indicating distress and impaired functioning as described by the World Health Organization [23]

### Search strategy

We searched five electronic databases (PubMed, Embase, Scopus, Web of Science, and PsycINFO) by using a comprehensive search strategy structured around three main concepts: (i) mental health outcomes, (ii) temperature exposure, and (iii) the population of children and young people (Supplementary Section B, Table B1).

### Eligibility criteria

We included epidemiological studies that quantitatively assessed the associations between ambient temperatures or heatwaves and mental health outcomes among children and young people (aged 0–25 years). We excluded studies that (i) focused solely on body temperature, subjective temperature, or indoor temperature as the exposure; (ii) did not include children and young people; (iii) addressed only physical health outcomes; or (iv) were not published in English. Our inclusion criteria used the participants–exposure–comparisons–outcome (PECO) framework [18] (Table 1).

### Study selection and data extraction

Two co-authors (S.K., S.F.) independently screened titles, abstracts, and full texts. We resolved discrepancies through discussions with a third co-author (J.S.). We extracted information from each eligible article, including author, publication year, location, age group, population, study period, study design, analytical methods, measures of exposures, effect estimates, and corresponding reference temperature values. The quantitative synthesis ultimately included studies reporting morbidity-related mental health outcomes, such as emergency-department visits, hospital admissions, outpatient visits, crisis-support calls, and diagnosis-specific outcomes; mortality endpoints were not represented in the pooled analysis. We also extracted lag structures and, where multiple lag estimates were reported, preferentially selected cumulative lag-effect estimates while retaining the original lag window rather than imposing a uniform lag period across the studies (Supplementary Section C, Table C1). In addition, we extracted climate type, income status, and summary exposure values of the study region by using global datasets [24, 25].

Studies on high temperatures reported effect estimates by using per-degree increases, temperature percentiles or quintiles, or comparisons between extreme and reference temperatures. For percentile-based studies, we extracted the reference and maximum temperatures used to estimate risk. To enable quantitative synthesis, we standardized all estimates to reflect the risk/degree or/quintile increase, in line with the original metric used (Supplementary Section C, Table C1). We excluded Nori-Sarma *et al.* [26] because that study did not meet our predefined eligibility criteria in full (age group = 18–26 years). However, to evaluate its potential influence on the meta-analysis, we performed a separate sensitivity analysis incorporating that study. We additionally recorded whether analyses were conducted year-round or during restricted warm-season/summer periods, as this could have influenced the comparability of the temperature effect estimates across the studies. For heatwave studies, we extracted the effect estimates as relative risks without any conversion because they directly compared the risk of outcomes during heatwave and non-heatwave periods.

### Risk-of-bias assessment

We evaluated the risk of bias by using the Office of Health Assessment and Translation approach developed by the National Institutes of Environmental Health Sciences-National Toxicology Program [27]. The approach was developed to adapt the ‘grading of recommendations assessment, development, and evaluation’ (GRADE) method for environmental and occupational health based on design considerations that are specific and relevant [27]. We evaluated each study based on several components, including exposure assessment, outcome assessment, confounding bias, selection/recruitment bias, incomplete outcome data, selective reporting, conflicts of interest, and other potential sources of bias (Supplementary Section D, Table D1).

### Synthesis of evidence

We performed separate meta-analyses for studies with high temperature and heatwaves as the exposure. We used the method of DerSimonian and Laird [28] and obtained overall estimates by using random-effects models, which accounts for both within- and among-study variability [28]. For studies reporting multiple relative risk estimates across subgroups or mental health outcomes, we used fixed-effect pooling to derive a single study-level estimate for the main meta-analysis and avoid double-counting (Supplementary Section C, Table C1). However, because some estimates were derived from the same underlying study population, variance may have been underestimated where within-study correlations were present [29]. We performed subgroup analyses based on age classifications, climate zones, the income status of the country, and summary exposure values where evidence was adequate (*n *≥ 2 studies). In addition, to explore whether temporal coverage contributed to between-study heterogeneity, we conducted a subgroup analysis by study period (warm season vs year-round). We excluded studies in which diurnal temperature variation or annual mean temperature was used as an exposure metric or those with exposure–outcome definitions that were not directly comparable to the short-term temperature and heatwave measures used in the pooled meta-analysis. However, we included their findings in the narrative synthesis to ensure broader contextual representation. We assessed heterogeneity by using Cochrane’s Q and Higgins’ *I*^2^ statistics, and evaluated publication bias through funnel plots and Egger’s tests. We performed sensitivity analyses by using trim-and-fill and leave-one-out methods to assess the impact of missing and influential studies on the overall estimates.

### Quality of evidence

We assessed the quality of evidence by using the Navigation Guide methodology—a modified GRADE approach for observational studies [30]. Each body of evidence started with a baseline rating of ‘moderate’ that we evaluated across five domains: risk of bias, indirectness, inconsistency, imprecision, and publication bias. We downgraded evidence for concerns in these domains and upgraded when large effects, dose–response relationships, or confounding likely reduced the observed effect. We assigned final ratings as ‘high’, ‘moderate’, ‘low’, or ‘very low’.

## Results

### Study characteristics

**Figure 1:**
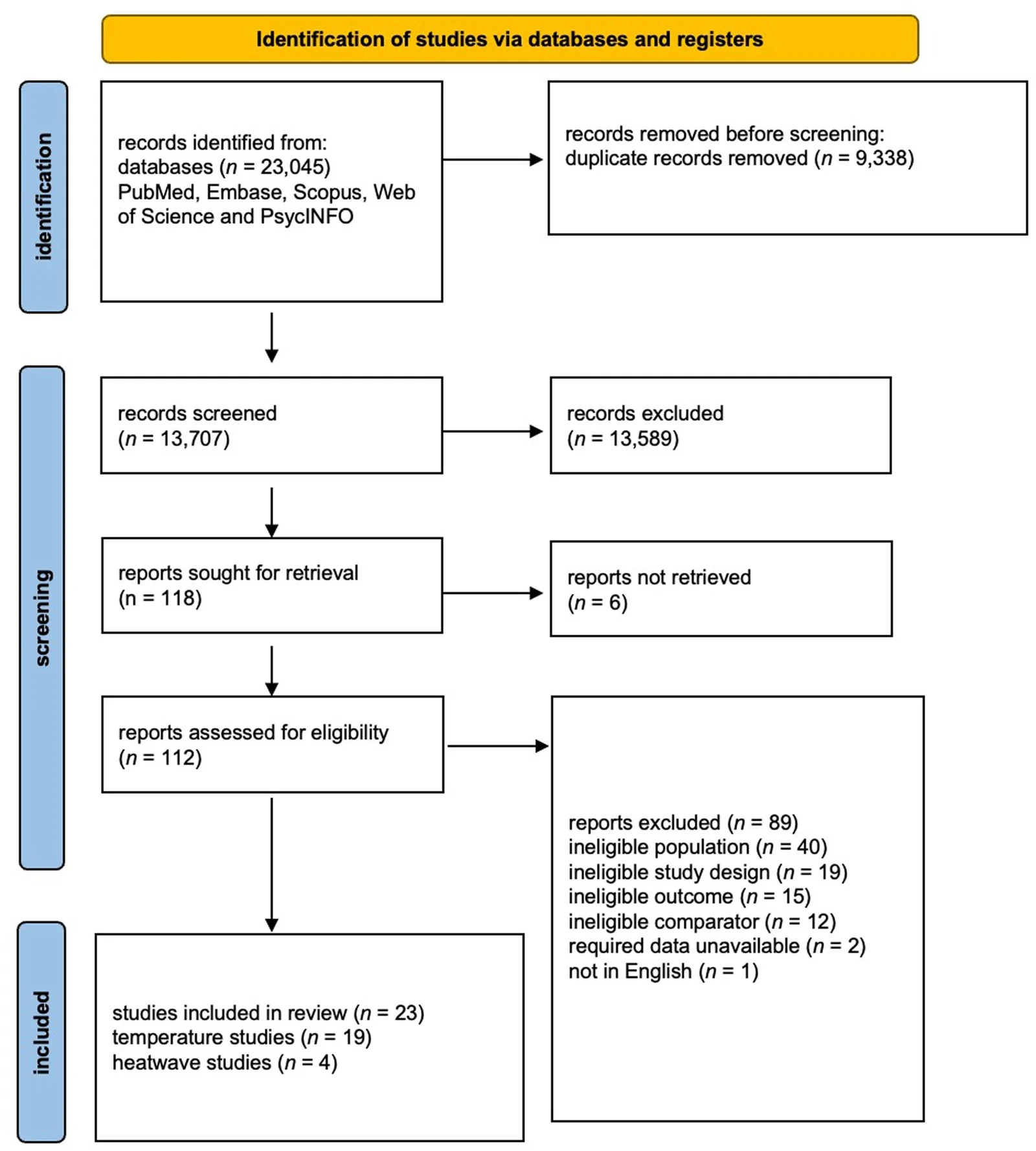
Flowchart depicting the study**-**selection process for identifying relevant studies (*n *= 23).

We identified 23 045 records, with 13 707 remaining after duplicate removal for title and abstract screening. After excluding 13 589 records, we assessed 118 full texts and included 23 studies in the meta-analysis and qualitative synthesis (Fig. 1) [17]. Supplementary Section E, Tables E1 and E2 present the study characteristics and main findings for high-temperature and heatwave studies.

**Figure 2:**
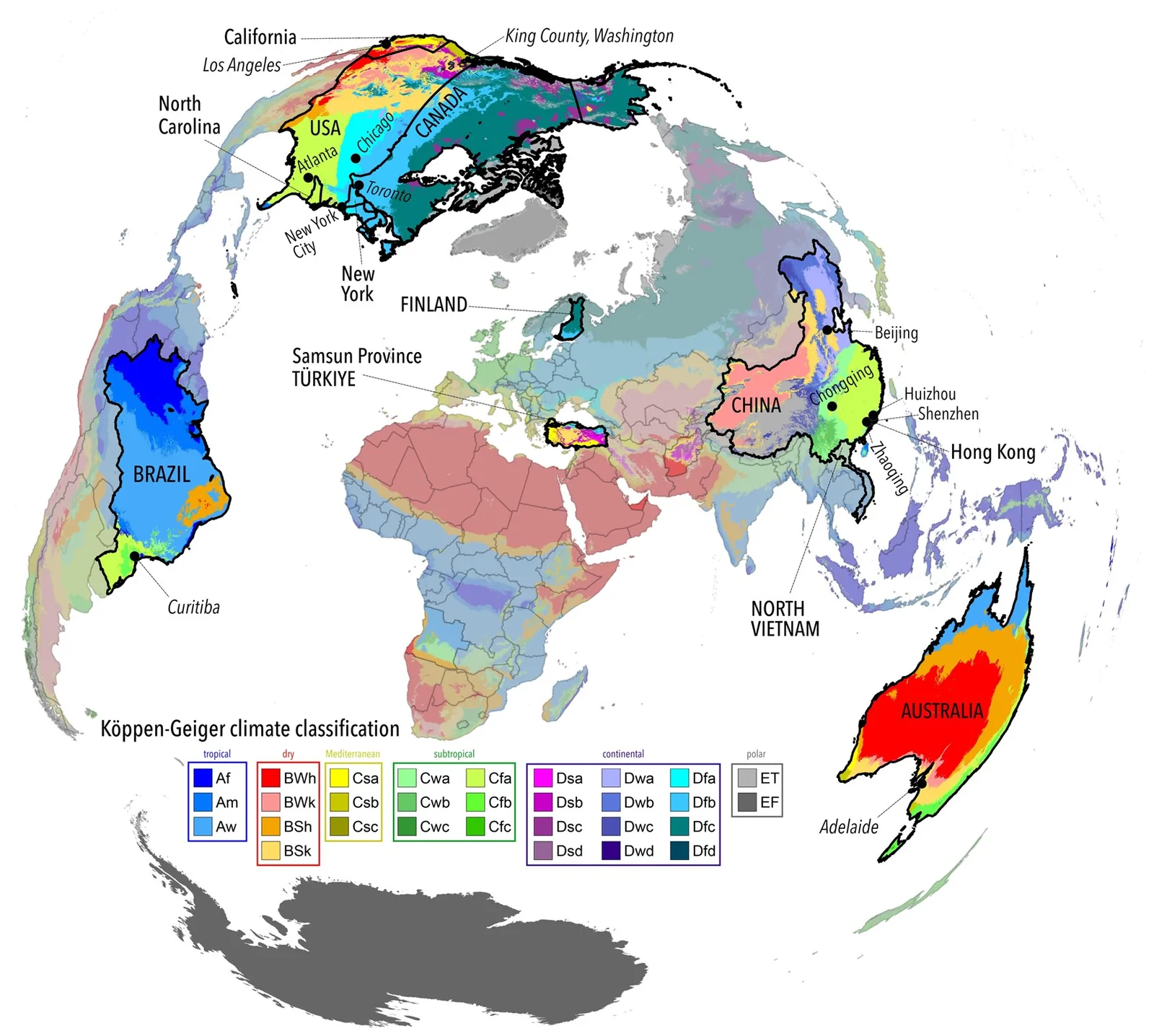
Global distribution of studies based on study regions, overlaid on the updated Köppen–Geiger global climate zones. The Köppen–Geiger classification system categorizes climate into five main groups: A (tropical): Af (tropical rain forest), Am (tropical monsoon), Aw (tropical savanna). B (dry): BWh (hot desert), BWk (cold desert), BSh (hot semi-arid), BSk (cold semi-arid). C (temperate): Csa, Csb, Csc (Mediterranean), Cwa (monsoon-influenced humid subtropical), Cwb, Cwc (subtropical highland). D (continental): Dsa, Dsb, Dsc, Dsd (Mediterranean-influenced), Dfa, Dfb, Dfc, Dfd (humid continental). E (polar): ET (tundra), EF (ice cap). Note: Af: tropical rainforest climate; Am: tropical monsoon climate; Aw: tropical savanna climate (dry winter); BWh: hot desert climate; BWk: cold desert climate; BSh: hot semi-arid (steppe) climate; BSk: cold semi-arid (steppe) climate; Csa: hot-summer Mediterranean climate; Csb: warm-summer Mediterranean climate; Csc: cold-summer Mediterranean climate (rare); Cwa: monsoon-influenced humid subtropical climate (dry winter, hot summer); Cwb: subtropical highland climate (dry winter, warm summer); Cwc: subtropical highland climate (dry winter, cool summer); Cfa: humid subtropical climate (no dry season, hot summer); Cfb: temperate oceanic climate (no dry season, warm summer); Cfc: subpolar oceanic climate (no dry season, cool summer); Dsa: hot-summer continental climate (dry summer); Dsb: warm-summer continental climate (dry summer); Dsc: cool-summer continental climate (dry summer); Dsd: extremely cold-summer continental climate (dry summer; very rare); Dwa: hot-summer continental climate (dry winter); Dwb: warm-summer continental climate (dry winter); Dwc: cool-summer continental climate (dry winter); Dwd: extremely cold-summer continental climate (dry winter); Dfa: hot-summer humid continental climate; Dfb: warm-summer humid continental climate; Dfc: subarctic climate (cool summer); Dfd: extremely cold subarctic climate.

All studies examining high temperatures (*n *= 19) and heatwaves (*n *= 4) as exposure were published between 2003 and 2024, collectively covering a population of ∼26 million. Most were performed in the USA (*n *= 10) [6, 15, 16, 31–37], followed by China (*n *= 3) [38–40], with no studies from the Pacific region or Africa. Three studies were undertaken in the Mediterranean climate zones (Csa/Csb/Csc) [6, 41, 42], four occurred in humid subtropical climate zones (Cfa) [15, 16, 32, 36], and several spanned multiple climate zones [6, 33, 34, 37, 39, 43] (Fig. 2).

### Risk-of-bias assessment

**Figure 3:**
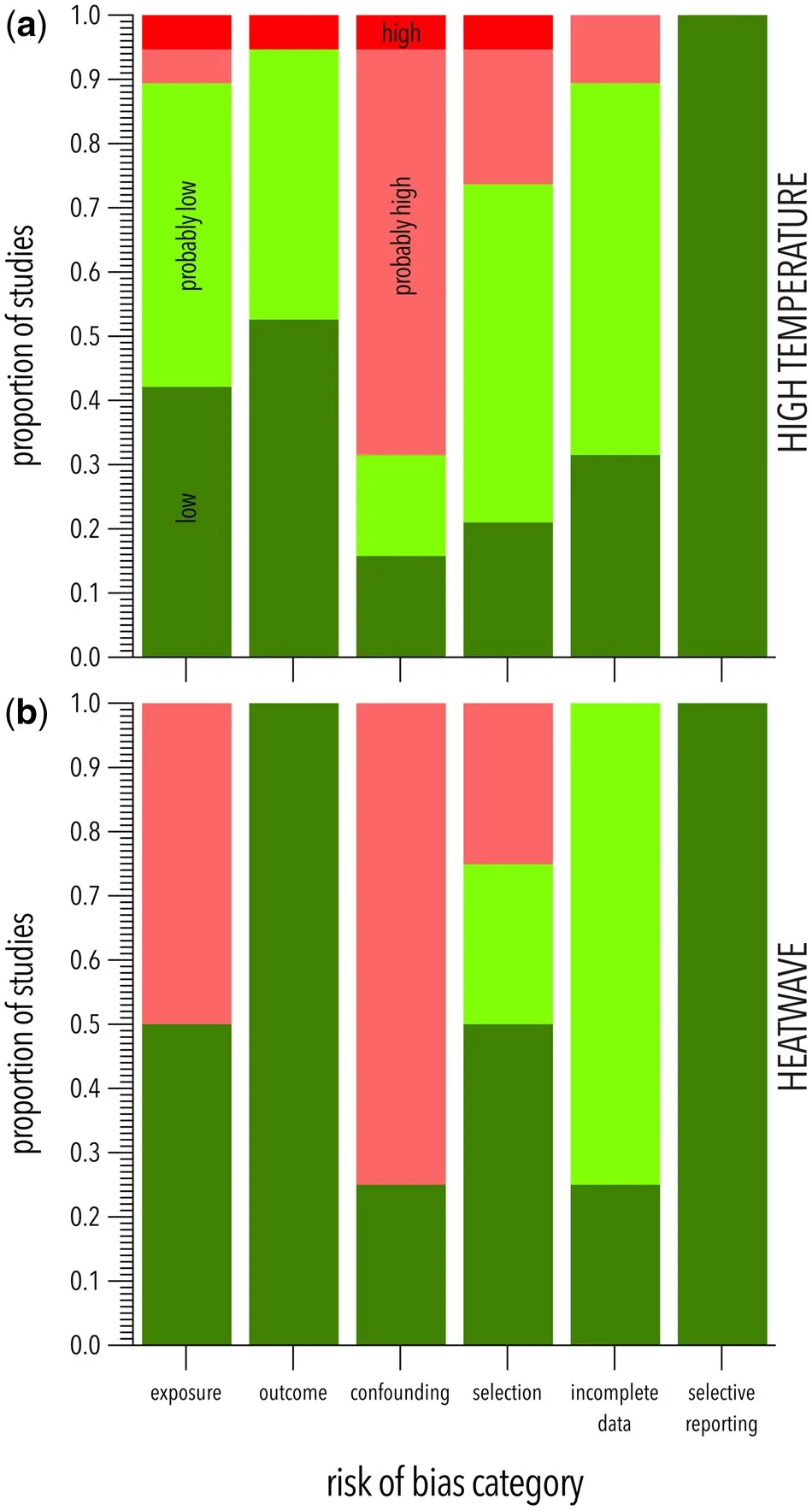
Risk-of-bias assessment for (a) high-temperature and (b) heatwave studies as per the four-point scale of the Office of Health Assessment and Translation tool.

We assessed the risk of bias within individual studies and across studies for both high-temperature (Fig. 3a) and heatwave exposure (Fig. 3b). Among the high-temperature studies, most showed a low risk of bias in outcome assessment (100%), selective reporting (100%), exposure assessment (89%), incomplete data (89%), and selection bias (74%). Only 32% of the high-temperature studies adequately controlled for confounding. For heatwave studies, exposure assessment, confounding, and selection bias were concerns in three out of four of the studies, while the other domains were generally rated as low-risk. Supplementary Materials (Sections F and G) provide detailed risk-of-bias assessments for individual high-temperature and heatwave studies, with summary assessments shown in Supplementary Figures F1 and G1.

### Synthesis of evidence

**Figure 4:**
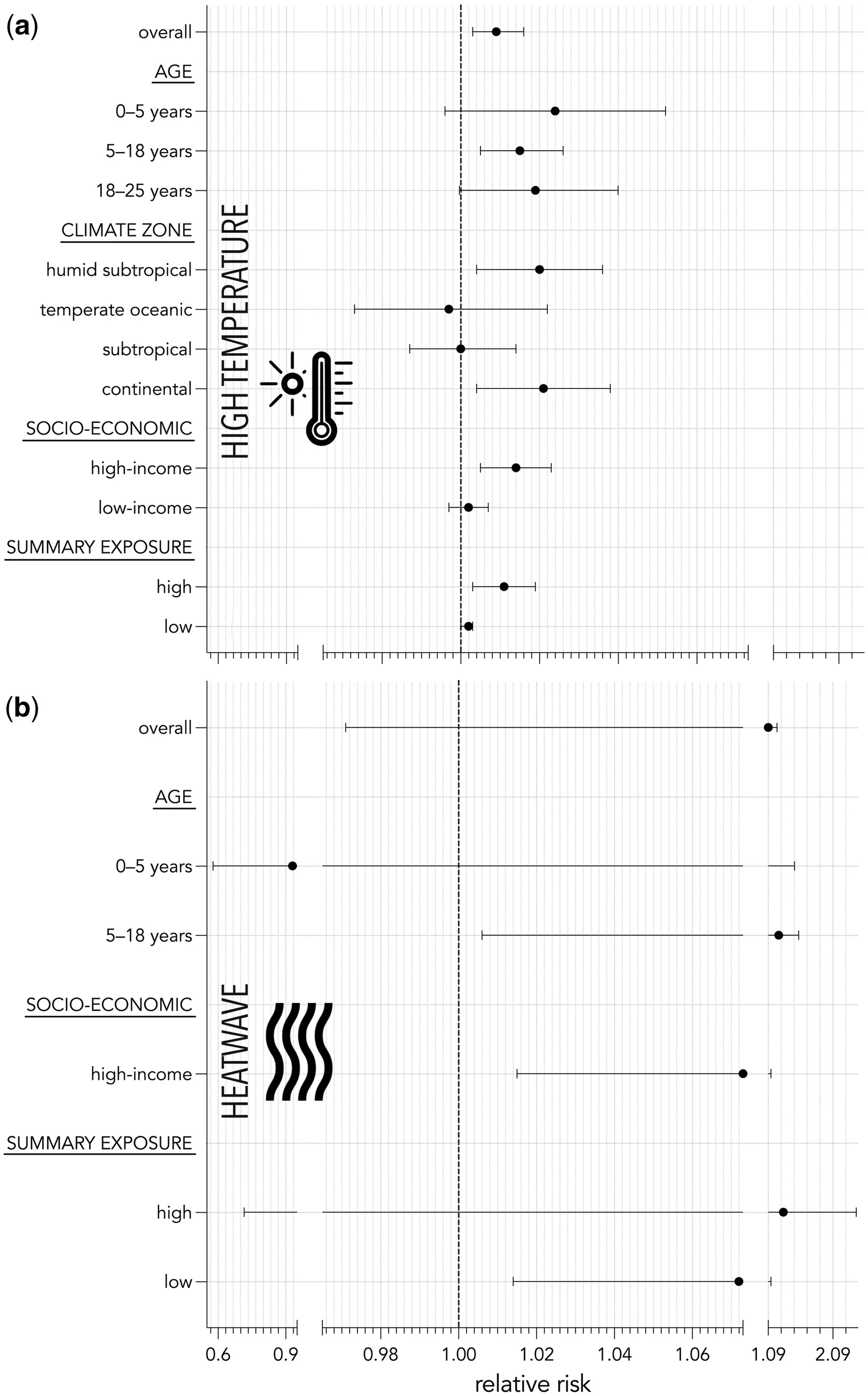
Subgroup meta-analysis of the association between (a) high temperature and adverse mental health outcomes in children and youth and (b) heatwaves and adverse mental health outcomes in children and youth. Points and error bars indicate RR and 95% CIs by age group, climate zone, socio-economic index, and summary exposure. The vertical dashed line indicates no effect (relative risk = 1). See Supplementary Material, Supplementary Sections H and I for additional associated statistics (type I error estimate, *I*^2^ = heterogeneity, and number of studies).

A meta-analysis of 14 studies indicated that each 1°C increase in temperature was associated with a 0.9% increase in the risk of mental health outcomes in children and young people [relative risk (RR) = 1.009; 95% confidence interval (CI): 1.003–1.016], with high heterogeneity (*I*^2^ = 82.61%, *P* < .001), where the *P* value is for heterogeneity (Fig. 4a and Supplementary Section H, Figure H1). An age-stratified analysis indicated no association in the age group of 0–5 years (*n *= 2) but we observed elevated risks in those aged 5–18 years (RR = 1.016; 95% CI: 1.005–1.027; *I*^2^ = 79.93%, *P* < .001) and 18–25 years (RR = 1.019; 95% CI: 0.998–1.040; *I*^2^ = 90.99%, *P* < .001) (Supplementary Section H, Figures H2–H12).

The highest relative risks were in the hot-summer humid continental climate (Dfa) (RR = 1.021; 95% CI: 1.004–1.038; *I*^2^ = 32.61%, *P* = .223) and humid subtropical climate (Cfa) (RR = 1.021; 95% CI: 1.004–1.039; *I*^2^ = 88.65%, *P* < .001), although the heterogeneity varied. In contrast, there were no associations in the temperate oceanic climate (Cfb) or monsoon-influenced humid subtropical climate (Cwa). Studies from high-income countries (*n *= 8) had a 1.4% increase in risk/1°C rise (RR = 1.014; 95% CI: 1.005–1.023; *I*^2^ = 81.26%, *P* < .001) whereas studies from middle-income countries (*n *= 6) had no clear association. The risk was also higher in countries with greater summary exposure (≥25, *n *= 11) (RR = 1.011; 95% CI: 1.003–1.019; *I*^2^ = 78.16%, *P* < .001) compared with those with lower summary exposure (*n *= 3) (RR = 1.002; 95% CI: 1.000–1.003; *I*^2^ < 0.01%, *P* = .429). In an additional subgroup analysis by study period, the pooled RR was 1.011 (95% CI: 1.001, 1.021; *I*^2^ = 81.07%, *P* < .001) for warm-season studies and 1.007 (95% CI: 0.998, 1.017; *I*^2^ = 77.51%, *P* < .001) for year-round studies (Supplementary Section H, Figures H13 and H14).

A meta-analysis of four studies assessing heatwave exposure showed no association with adverse mental health outcomes in children and young people (RR = 1.092; 95% CI: 0.971–1.227; *I*^2^ = 15.57%, *P* = .141) (Fig. 4b and Supplementary Section I, Figure I1). An age-stratified analysis indicated no association in children aged 0–5 years whereas those aged 5–18 years had a 25.3% higher risk (RR = 1.253; 95% CI: 1.006–1.561; *I*^2^ = 19.7%, *P* = .0076). No data were available for individuals aged ≥18 years. A subgroup analysis by socio-demographic index showed a positive association in high-income countries (*n *= 3) (RR = 1.073; 95% CI: 1.015–1.135; *I*^2^ = < 0.01%, *P* = .013). Similarly, studies from countries with low summary exposure values (*n *= 2) had a 7.2% increased risk (RR = 1.072; 95% CI: 1.014–1.134; *I*^2^ < 0.01%, *P* = .015) whereas those from countries with high summary exposure values (*n *= 2) reported no clear association (Supplementary Section I, Figures I2–I6).

### Publication bias and sensitivity analysis

The high-temperature studies were heterogenous (*I*^2^ = 80.1%) but there was no evidence of publication bias or missing studies (Table 2). Sensitivity analyses, including leave-one-out and the inclusion of Nori-Sarma *et al.* [53], confirmed the robustness of the pooled estimate (RR = 1.009; 95% CI: 1.003–1.016) (Supplementary Section H, Figures H15–H18).

**Table 2:** Publication bias and sensitivity analysis for high-temperature studies.

Analysis type	Estimated missing value	*τ* ^2^ (SE)	*I* ^2^ (%)	*Q* (type I error)	Pooled risk ratio [RR (95% CI)]	Conclusion
Egger’s test	–	–	–	–	Intercept = 0.0016 (95% CI: –0.0022 to 0.0055), *P* = .1216	Evidence of publication bias
Trim and fill (left)	0	–	–	–	NA	No adjustment for missing studies
Overall	0	0.00004 (0.0081)	82.61	*Q *= 74.77 (*P* < .01)	1.009 (95% CI: 1.003–1.016)	Evidence of substantial heterogeneity

*P*, type I error; *I*^2^, heterogeneity; *Q*, *Q* statistic; τ², between-study variance; –, not applicable or not estimated.

Heatwave studies had low heterogeneity (*I*^2^ = 15.6%) and no evidence of publication bias (Table 3). The trim-and-fill method identified one potentially missing study and the adjusted pooled estimate indicated a stronger association (RR = 1.083; 95% CI: 1.026–1.143) (Supplementary Section I, Figures I8 and I9).

**Table 3:** Publication bias and sensitivity analysis for heatwave studies.

Analysis type	Estimated missing studies	*τ* ^2^ (SE)	*I* ^2^ (%)	*Q* (type I error)	Pooled risk ratio [RR (95% CI)]	Conclusion
Egger’s test	–	–	–	–	Intercept = 0.069 (95% CI: –0.1369 to 0.2757), *P* = .7759	No evidence of publication bias
Trim and fill (left)	1	0.0 (0.0192)	0.0	*Q *= 5.54 (*P *= .59)	1.083 (95% CI: 1.026–1.143)	Adjusted pooled effect after imputing one missing study
Overall		0 (0.1472)	15.57 (*P *= .141)	*Q *= 3.553 (*P *= .314)	1.092 (95% CI: 0.971–1.227)	Pooled effect with no heterogeneity

*P*, type I error; *I*^2^, heterogeneity; *Q*, *Q* statistic; τ², between-study variance; –, not applicable or not estimated.

We rated the overall quality of evidence as moderate for both high**-**temperature and heatwave exposures, with dose–response relationships observed, but limitations due to heterogeneity (high temperature) and imprecision (heatwave) (Supplementary Section J, Table J1). The findings from five studies excluded from the meta-analysis are summarized in Supplementary Section K, Table K1 and were broadly consistent with an adverse effect of heat exposure on mental health outcomes.

## Discussion

This is the first systematic review and meta-analysis to have synthesized global evidence on the mental health impacts of heat exposure in children and young people. We found a 0.9% increase in broad morbidity-related mental health outcomes per 1°C rise in daily temperature. We found no clear association for heatwaves, likely due to limited data. Children aged 5–18 years were the most vulnerable, with a 1.5% increase for high temperatures and 25% for heatwaves. The risks were highest in humid subtropical climate (Cfa) and hot-summer humid continental climate (Dfa) zones (2% per 1°C), in high-income countries (1.4%), and in regions with high summary exposure values (1.1%).

The heightened vulnerability in young children and adolescents may reflect social and developmental stressors, including disruptions to outdoor activities, structured education, and recreational opportunities. Additional pathways may include sleep disruption during hot nights, which can affect well-being and emotional regulation, as well as indirect effects through parental mental health, caregiving stress, and family conflict [44, 45]. Adolescence is also a sensitive period for mental health development, which may increase susceptibility to environmental stressors such as extreme heat [46]. Higher temperatures have also been associated with poor learning and academic performance in children [47, 48].

From a climate-zone perspective, the combined effects of intense heat and humidity in hot, humid regions could exacerbate both physical discomfort and psychological stress. The higher risks observed in high-income countries may partly reflect urban heat island effects, as well as surveillance and ascertainment bias, because stronger mental health diagnostic infrastructure, more complete reporting, and higher healthcare-seeking behaviour increase the likelihood that heat-related mental health presentations are captured in routine datasets [49, 50]. In contrast, the weaker associations observed in middle-income countries may reflect under-reporting, limited mental health surveillance systems, and differences in healthcare infrastructure rather than lower true vulnerability. However, when stratified by summary exposure, countries with higher summary exposure consistently had elevated risks, highlighting that cumulative heat exposure—not just income—could be an important driver of vulnerability.

These findings also highlight important gaps in the current evidence base and priorities for future research, which should prioritize under-represented settings, particularly in low- and middle-income countries and among socio-economically disadvantaged populations, and use longitudinal designs to clarify the long-term mental health impacts of sustained heat exposure in childhood [16, 51]. Most of the included studies assessed broad International Classification of Diseases (ICD)-coded mental disorders (F00–F99) rather than condition-specific estimates. Evidence was particularly limited for subclinical distress and for conditions such as conduct disorders, autism, and eating disorders.

Interpretation of the heatwave findings also requires caution, as heatwave effects may be delayed and become more apparent during prolonged events rather than after a single day of extreme heat [52, 53]. More research is required to clarify the association between heatwaves and mental health outcomes in children and young people.

Our study has some limitations. Substantial heterogeneity across high-temperature studies limits the generalizability of the pooled estimates. Although we applied harmonization procedures and explored heterogeneity through subgroup analyses by age, climate zone, socio-economic context, summary exposure, and study period (warm season vs year-round), heterogeneity remained substantial across most of the subgroups. This suggests that residual heterogeneity likely reflects genuine differences across studies in populations, climatic and geographic settings, lag structures, exposure definitions and metrics (e.g. per 1°C, percentiles/quintiles, study-specific heat thresholds), analytical approaches, outcome definition, and ascertainment. Although mortality endpoints were not included in the pooled analysis, the reviewed studies varied substantially in morbidity-related mental health outcomes, including emergency-department visits, hospital admissions, outpatient visits, crisis-support calls, and diagnosis-specific outcomes. This variation in outcome setting, severity, and clinical specificity may have influenced both the magnitude and interpretation of the pooled estimates. More detailed subgroup analyses by outcome type were not feasible because of the limited number of studies within individual outcome categories.

Subgroup analyses based on a few studies (*n *= 2–4) reduce reliability and should be interpreted cautiously. This was particularly relevant for children aged 0–5 years and those aged >18 years, for whom only two studies were available, as well as for several other subgroup categories. These estimates are therefore less stable and more sensitive to individual study characteristics. In addition, the overall number of studies on heatwave exposure was limited, so findings for heatwave-related mental health outcomes should also be interpreted cautiously and cannot yet be generalized without further evidence. We were also unable to assess condition-specific effects because most of the included studies reported aggregated mental health outcomes, often combining diverse disorders such as depression, anxiety, ADHD, and neurodevelopmental conditions [54]. This diagnostic overlap could have obscured condition-specific associations. For studies reporting multiple estimates, deriving a single study-level estimate may have underestimated variance where estimates were correlated within the same underlying study population. This may have led to some overestimation of precision. Our review was also limited to English-language studies, potentially excluding relevant research from under-represented regions, and inconsistent definitions of exposures and outcomes (e.g. ‘heatwave’, ‘mental health’) could have contributed to variability.

Residual confounding is another important limitation. Although we assessed confounding in the risk-of-bias evaluation, only 32% of the high-temperature studies adequately controlled for it. Important correlated factors, including air pollution, seasonal influences, and individual-level socio-economic conditions, were not consistently accounted for across the studies and may therefore have partly contributed to the observed associations.

## Conclusions

Our review emphasizes the growing vulnerability of child and adolescent mental health in the context of a warming climate. These findings support the need to integrate mental health into climate-adaptation efforts and to develop targeted interventions for this demographic. However, evidence for heatwave-related mental health outcomes remains limited and conclusions for this exposure should be interpreted cautiously until more primary research becomes available. This review also reveals major data gaps in the hottest and most disadvantaged regions. Future research should prioritize these areas, use standardized methods and longitudinal designs, and explore diagnostic pathways to inform equitable, climate-resilient mental health strategies for children and young people.

## Ethics approval

Ethics approval was not required because this study was a systematic review and meta-analysis of data from previously published studies and did not involve the recruitment of human participants, collection of new participant data or access to identifiable individual-level information. 

## Supplementary Material

dyag121_Supplementary_Data

## Data Availability

The data underlying this systematic review and meta-analysis were extracted from the published studies cited in the article. The study-level extracted data are available from the corresponding author upon reasonable request. The analytical code used for the primary overall meta-analysis of incremental hot-temperature exposure and associated diagnostic analyses is publicly available through GitHub and archived in Zenodo at https://doi.org/10.5281/zenodo.21458614.
