# Supplementary material for: First global synthesis of heat-related mental health impacts in children and young people: a systematic review and meta-analysis
Source: Int J Epidemiol. 2026 Jul 25;55(4):dyag121. doi: 10.1093/ije/dyag121 (PMC13401444; doi:10.1093/ije/dyag121)
Supplement: dyag121_Supplementary_Data [file dyag121_supplementary_data.docx]

# **First global synthesis of heat-related mental health impacts in children and young people: a systematic review and meta-analysis**

**Supplementary material**

Shevonne Kwan, Jacqueline Stephens, Corey J. A. Bradshaw, Blesson M. Varghese, Kirrilly Thompson, Melinda A Judge, Peter N. Le Souëf, Syeda H. Fatima

Table of Contents

**Section A – Prisma checklist**

Table A1. Prisma 2020 checklist ......................................................................................................................................................... 4

**Section B – Search strategy**

Table B1. Search strategy .................................................................................................................................................................... 6

Table B2. ICD 9, ICD 10 and ICD 11 codes ...................................................................................................................................... 7

**Section C – Data extraction and standardisation**

Table C1: Summary of Data Processing and Effect Estimate Conversions……………………………………………...…………..8

**Section D - Criteria for the quality assessment of each study**

Table D1. Criteria for risk assessment as per the Office of Health Assessment and Translation risk-of-bias rating tool ................. 10

**Section E – Study characteristics**

Table E1. Study characteristics and main findings for high temperature studies ................................................................................13

Table E2. Study characteristics and main findings for heatwave studies ............................................................................................17

**Section F – Assessment of risk of bias in individual studies for temperature studies**

Table F1. Risk-of-bias assessment of Aydin-Ghormoz et al., 2022 ................................................................................................... 18

Table F2. Risk-of-bias assessment of Basu et al., 2018 ...................................................................................................................... 19

Table F3. Risk-of-bias assessment of Bernstein et al., 2022 .............................................................................................................. 20

Table F4. Risk-of-bias assessment of Calkins et al., 2016 ................................................................................................................. 21

Table F5. Risk-of-bias assessment of Chan et al., 2018 ..................................................................................................................... 22

Table F6. Risk-of-bias assessment of Cohen et al., 2024 ................................................................................................................... 23

Table F7. Risk-of-bias assessment of Corvetto et al., 2023 ................................................................................................................ 24

Table F8. Risk-of-bias assessment of DaSilva et al., 2020 ................................................................................................................. 25

Table F9. Risk-of-bias assessment of Deng et al., 2022 ..................................................................................................................... 26

Table F10. Risk-of-bias assessment of Doganay et al., 2003 ............................................................................................................. 27

Table F11. Risk-of-bias assessment of Komulainen et al., 2022 ........................................................................................................ 28

Table F12. Risk-of-bias assessment of Niu et al., 2024 ...................................................................................................................... 29

Table F13. Risk-of-bias assessment of Niu et al., 2023 ..................................................................................................................... 30

Table F14. Risk-of-bias assessment of Stowell et al., 2023................................................................................................................ 31

Table F15. Risk-of-bias assessment of Sugg et al., 2019 ................................................................................................................... 32

Table F16. Risk-of-bias assessment of Wang et al., 2014 .................................................................................................................. 33

Table F17. Risk-of-bias assessment of Younan et al., 2018 ............................................................................................................... 34

Table F18. Risk-of-bias assessment of Zhang et al., 2020 ................................................................................................................. 35

Table F19. Risk-of-bias assessment of Zhou et al., 2023 ................................................................................................................... 36

Figure F1. Risk assessment of high temperature studies as per the 4-point scale of the Office of Health Assessment and Translation tool ..................................................................................................................................................................................................... 37

**Section G – Assessment of risk of bias in individual studies for heatwave studies**

Table G1. Risk-of-bias assessment of Nitschke et al., 2007 ............................................................................................................... 38

Table G2. Risk-of-bias assessment of Nitschke et al., 2011 ............................................................................................................... 39

Table G3. Risk-of-bias assessment of Sewell et al., 2024 .................................................................................................................. 40

Table G4. Risk-of-bias assessment of Trang et al., 2016 ................................................................................................................... 41

Figure G1. Risk assessment of heatwave studies as per the 4-point scale of the Office of Health Assessment and Translation tool.

............................................................................................................................................................................................................ 42

**Section H – Results for temperature studies**

Figure H1. Forest plot of meta-analytical results for temperature studies (*n* = 14) ............................................................................ 43

Subgroup analysis with respect to climate zones ............................................................................................................................... 44

Figure H2. Forest plot for climate 1 analysis: humid subtropical climate (Cfa) ............................................................................... 44

Figure H3. Forest plot for climate 2 analysis: temperate oceanic climate (Cfb) ............................................................................... 44

Figure H4. Forest plot for climate 4 analysis: humid subtropical climate (Cwa) .............................................................................. 45

Figure H5. Forest plot for climate 5: hot-summer humid continental climate (Dfa) ........................................................................ 45

Subgroup analysis with respect to ages ............................................................................................................................................. 46

Figure H6. Forest plot for age group 1 .............................................................................................................................................. 46

Figure H7. Forest plot for age group 2 .............................................................................................................................................. 46

Figure H8. Forest plot for age group 3 ............................................................................................................................................... 47

Subgroup analysis with respect to income status of the country......................................................................................................... 48

Figure H9. Forest plot for high income countries based on socio-demographic index ranking ........................................................ 48

Figure H10. Forest plot for middle income countries based on socio-demographic index ranking .................................................. 48

Figure H11. Forest plot for high exposure countries based on summary exposure value ranking .................................................... 49

Figure H12. Forest plot for low exposure countries based on summary exposure value ranking ..................................................... 49

Figure H13. Forest plot of pooled risk estimates for studies restricted to the warm season……………………………………….50

Figure H14. Forest plot of pooled risk estimates for studies using year-round data……………………………………………….50

Figure H15. Leave-one-out analysis .................................................................................................................................................. 51

Figure H16. Funnel plot for Egger’s test ........................................................................................................................................... 52

Figure H17. Funnel plot for trim and fill test .................................................................................................................................... 53

Figure H18. Forest plot for meta-analytical results of temperature studies including one extra study that partly fills the inclusion criteria (*n* = 15) .................................................................................................................................................................................. 53

**Section I – Results for Heatwave Studies**

Figure I1. Forest plot of meta-analytical results.................................................................................................................................. 54

Subgroup analysis with respect to age groups ................................................................................................................................... 55

Figure I2. Forest plot for age group 1 ................................................................................................................................................ 55

Figure I3. Forest plot for age group 2 ................................................................................................................................................ 55

Figure I4. Forest plot for high income countries based on socio-demographic index ranking .......................................................... 56

Figure I5. Forest plot for high exposure countries based on summary exposure value ranking ....................................................... 56

Figure I6. Forest plot for low exposure countries based on summary exposure value ranking ........................................................ 57

Figure I7. Leave-one-out analysis ...................................................................................................................................................... 57

Figure I8. Funnel plot for Egger’s test ............................................................................................................................................... 58

Figure I9. Funnel plot for trim and fill test ......................................................................................................................................... 58

**Section J – Quality of evidence**

## Table J1. Quality of evidence in studies investigating the association between heat exposure and mental health outcomes .......... 59

**Section K – Narrative Synthesis**

**Table K1.** Narrative synthesis of studies excluded from the meta-analysis……………………………………………………….-60

**Section A – Prisma checklist**

**Table A1.** Prisma Checklist. SM = supplementary material.

| **section and topic** | **item #** | **checklist item** | **location where item**  **reported** |
| --- | --- | --- | --- |
| **title** | | |  |
| title | 1 | identify the report as a systematic review. | page 1 |
| **abstract** | | |  |
| abstract | 2 | see the prisma 2020 for abstracts checklist. | Page 2 |
| **introduction** | | |  |
| rationale | 3 | describe the rationale for the review in the context of existing knowledge. | page 4 |
| objectives | 4 | provide an explicit statement of the objective(s) or question(s) the review addresses. | page 4 |
| **methods** | | |  |
| eligibility criteria | 5 | specify the inclusion and exclusion criteria for the review and how studies were grouped for the syntheses. | page 5 |
| information sources | 6 | specify all databases, registers, websites, organisations, reference lists and other sources searched or consulted to identify studies. specify the date when each source was last searched or consulted. | page 6-7 and SM |
| search strategy | 7 | present the full search strategies for all databases, registers and websites, including any filters and limits used. | SM |
| selection process | 8 | specify the methods used to decide whether a study met the inclusion criteria of the review, including how many reviewers screened each record and each report retrieved, whether they worked independently, and if applicable, details of automation tools used in the process. | page 6-8 and SM |
| data collection process | 9 | specify the methods used to collect data from reports, including how many reviewers collected data from each report, whether they worked independently, any processes for obtaining or confirming data from study investigators, and if applicable, details of automation tools used in the process. | page 7-8 and SM |
| data items | 10a | list and define all outcomes for which data were sought. specify whether all results that were compatible with each outcome domain in each study were sought (e.g. for all measures, time points, analyses), and if not, the methods used to decide which results to collect. | page 7-8 and SM |
|  | 10b | list and define all other variables for which data were sought (e.g. participant and intervention characteristics, funding sources). describe any assumptions made about any missing or unclear information. | page 7-8 and SM |
| study risk-of-bias assessment | 11 | specify the methods used to assess risk of bias in the included studies, including details of the tool(s) used, how many reviewers assessed each study and whether they worked independently, and if applicable, details of automation tools used in the process. | page 8 and SM |
| effect measures | 12 | specify for each outcome the effect measure(s) (e.g. risk ratio, mean difference) used in the synthesis or presentation of results. | page 7-8 and SM |
| synthesis methods | 13a | describe the processes used to decide which studies were eligible for each synthesis (e.g. tabulating the study intervention characteristics and comparing against the planned groups for each synthesis [item #5]). | page 11-12 |
|  | 13b | describe any methods required to prepare the data for presentation or synthesis, such as handling of missing summary statistics, or data conversions. | page 7-8 and SM |
|  | 13c | describe any methods used to tabulate or visually display results of individual studies and syntheses. | page 7-8 and SM |
|  | 13d | describe any methods used to synthesize results and provide a rationale for the choice(s). if meta-analysis was performed, describe the model(s), method(s) to identify the presence and extent of statistical heterogeneity, and software package(s) used. | page 7-8 and SM |
|  | 13e | describe any methods used to explore possible causes of heterogeneity among study results (e.g. subgroup analysis, meta-regression). | page 7-87 and SM |
|  | 13f | describe any sensitivity analyses conducted to assess robustness of the synthesised results. | page 7-8 and SM |
| reporting bias assessment | 14 | describe any methods used to assess risk of bias due to missing results in a synthesis (arising from reporting biases). | pages 7-8 and SM |
| certainty assessment | 15 | describe any methods used to assess certainty (or confidence) in the body of evidence for an outcome. | pages 7-8 and SM |

*SM: Supplementary Materials*

*From:* Page MJ, McKenzie JE, Bossuyt PM, Boutron I, Hoffmann TC, Mulrow CD, et al. The PRISMA 2020 statement: an updated guideline for reporting systematic reviews. BMJ 2021;372:n71. doi:10.1136/bmj.n71.

**Section B — Search strategy**

**Table B1.** Search strategy.

| key concepts | key words | mesh terms |
| --- | --- | --- |
| mental health outcomes | mental illnesses, developmental delay, anxiety, depression, attention deficit disorder, attention-deficit hyperactivity disorder, mental health disorders, hyperkinetic disorders, mood disorder, emotional disorder, mental health outcomes, mental disorder, nervous disorder, mental health problems, mental health condition, psychiatric disability, psychological disorders, psychological stress | - "mental disorders"[MeSH Terms] - emotions"[MeSH Terms] AND ("disease"[MeSH terms] - "mood disorders"[MeSH Terms] - hyperkinetic"[All Fields]) AND ("disease"[MeSH terms] - "mental health"[MeSH Terms] - "mental health"[All Fields]) AND ("disease"[MeSH terms] - "anxiety"[MeSH terms] - ("attention deficit disorder with hyperactivity"[MeSH terms] - "depression"[MeSH terms] - "depressive disorder"[MeSH terms] |
| temperature | climate, weather, temperature zones, climate zone, climate change, hot weather, high temperature, low temperature, ambient temperature | - "climate"[MeSH terms] - weather"[MeSH terms] - "temperature"[MeSH terms] - "climate change"[MeSH terms] - "heatwave"[all fields] - "ambient"[all fields] - “weather"[MeSH terms] |
| population | child, toddler, school aged, young people, adolescent, teenager, primary school aged, preschool, youth, minors, kid | - "child"[MeSH terms] - "adolescent"[MeSH terms] - "school"[all fields]) AND ("aged"[MeSH terms] - primary school"[all fields]) AND ("aged"[MeSH terms] |

**Table B2:** ICD 9, ICD 10 and ICD 11 codes.

| Mental health outcome | ICD-9 Code | ICD-10 Code | ICD-11 Code |
| --- | --- | --- | --- |
| mood (affective) disorders | 296.x, 300.4, 311 | F30-F39 | 6A60-6A8Z (mood disorders) |
| major depressive disorder | 296.2, 296.3, 311 | F32.x, F33.x | 6A70 (single episode), 6A71 (recurrent) |
| bipolar disorder | 296.0, 296.1, 296.4-296.7 | F30, F31 | 6A60 (bipolar disorders) |
| anxiety disorders | 300.0, 300.2, 300.3 | F40-F41 | 6B00-6B0Z (anxiety or fear-related) |
| obsessive-compulsive disorder (OCD) | 300.3 | F42 | 6B20 (obsessive-compulsive disorder) |
| post-traumatic stress disorder (PTSD) | 309.81 | F43.1 | 6B40 (post-traumatic stress disorder) |
| schizophrenia and psychotic disorders | 295.x, 297.x, 298.x | F20-F29 | 6A20-6A2Z (schizophrenia spectrum) |
| substance use disorders | 303.x, 304.x | F10-F19 | 6C40-6C4Z (substance use disorders) |
| eating disorders | 307.1, 307.5 | F50 | 6B80 (eating disorders) |
| suicidal behaviour | E950-E959 | X60-X84, Y87.0 | MB21 (suicidal behaviour) |
| personality disorders | 301.x | F60-F69 | 6D10-6D1Z (personality disorders) |
| somatic symptom disorders | 300.7, 300.8 | F45 | 6B40-6B4Z (somatic symptom disorders) |
| neurodevelopmental disorders (e.g., ADHD) | 314.x | F90 | 6A05 (attention deficit hyperactivity) |
| conduct disorders | 312.x | F91 | 6C90 (conduct-dissocial disorder) |
| autism spectrum disorder | 299.0 | F84.0 | 6A02 (autism spectrum disorder) |
| dementia and cognitive disorders | 290.x | F00-F03 | 6D80-6D89 (neurocognitive disorders) |
| sleep-wake disorders | 307.4 | F51 | 7A20-7A2Z (sleep-wake disorders) |
| intellectual disabilities | 317-319 | F70-F79 | 6A00-6A0Z (intellectual developmental) |

**Section C — Data extraction and standardisation**

We systematically extracted key information from each eligible study into an Excel® spreadsheet, including author, year of publication, geographic location, Köppen–Geiger climate classification, age group, population characteristics, study period, design, analytical methods, exposure measures, and reported effect estimates. Age categories were harmonised across studies for subgroup analyses; however, the original study-specific age bands were not fully consistent and, in some cases, partially overlapped. Climate zones were assigned using the global Köppen–Geiger classification map. We also extracted two contextual variables for each study location: income level based on the Socio-demographic Index (SDI), and Summary Exposure Value (SEV) for environmental risk from the Global Health Data Exchange. Effect estimates were sourced from text, tables, figures, and supplementary files; where necessary, we contacted study authors to clarify or obtain missing data.

Studies used diverse approaches to quantify heat-related effects. Some reported percentage changes or relative risks per 1 °C increase in temperature, while others used comparisons between percentiles, quintiles, or defined extreme heat thresholds relative to reference values. For percentile-based studies, we also recorded the reference and peak temperatures used. To facilitate quantitative synthesis, we harmonised effect estimates to a comparable metric representing risk per 1 °C or per-quintile increase, depending on the original format used by each study. Table C1 provides summary of the formulas sued for conversions. In studies reporting multiple temperature metrics, we preferentially selected the estimate based on mean or average temperature where available. Where this was not reported for the relevant child or adolescent subgroup, we extracted the estimate corresponding to the most comparable available heat exposure metric reported for that subgroup.

**Handling of lag structures and multiple estimates within studies.** Some of the included studies (1-3) used different lag structures (e.g., cumulative lag 0–2, 0–5, or 0–30 days). For quantitative synthesis, we preferentially extracted cumulative lag effect estimates as reported by the original study authors, where available, and retained the reported lag window rather than imposing a uniform lag period across studies. When a study reported multiple eligible estimates for the same exposure category (e.g., by age group, sex, outcome subtype, dataset, or location), we combined these estimates using inverse-variance fixed-effect pooling to derive a single study-level estimate for the primary meta-analysis and avoid over-representation of individual studies.

**Table C1:** Summary of Data Processing and Effect Estimate Conversions

| **Item** | **Description** |
| --- | --- |
| Percent change, odds ratios per unit temperature increase | Applied:  p = 100(r−1)  r = o/[(1−i₀) + oi₀]  where p = % change, r = RR, o = OR, and i₀ = baseline incidence. When i₀ was unavailable, assumed RR = OR. |
| Adjustment of per-X °C effects | Converted estimates reported per X °C above reference to per-1 °C using: E₁ = E / X. |
| Relative risk estimates based on percentiles | Converted percentile-based RR to log-RR assuming a log-linear relationship:  RR_per 1°C_ = exp[log(RR_total_) ⁄ ΔT]  where ΔT is the temperature difference between percentiles. |
| Estimates using log-likelihood values and p-values | When effect estimates are given as log-likelihood values and p values, we estimated confidence intervals: CI = exp[log(RR) ± Z × SE*]  with SE approximated from likelihood ratios. |
| Studies reporting estimates for quintiles of temperature | For studies reporting relative risk by temperature quintile, we estimated per-quintile risk assuming a linear association. |
| Lag structure handling | Preferentially extracted cumulative lag estimates (as reported; e.g., lag 0–2, 0–5, 0–30). No uniform lag window was imposed across studies due to inconsistent reporting. Where multiple eligible estimates were reported within a study, inverse-variance fixed-effect pooling was used to derive one study-level estimate. |

*SE (Standard Error) was approximated using the formula: SE ≈ √[1 / (2 × (Lnull – Lmodel))], where Lnull and Lmodel are the log-likelihoods under the null and alternative hypotheses, respectively.

**Section D — Criteria for the quality assessment of each study**

**Table D1.** Criteria for the quality assessment of each study, modified according to the Office of Health Assessment and Translation risk-of-bias rating tool (4).

| bias | risk-of-bias questions | answer |
| --- | --- | --- |
| key criteria | | |
| exposure assessment  *includes measurement error or measurement limitations* | considerations:  1) number of temperature related monitoring station per geographical area  2) daily ambient temperature measurements were available | LOW: There is high confidence that the exposure to ambient temperature is the true average population exposure.  PROBABLY LOW: There is indirect evidence that suggests low risk of bias.  PROBABLY HIGH: There is insufficient information to permit a judgment of high risk of bias, but there is indirect evidence that suggests high risk of bias.  HIGH: There is direct evidence of high risk of misclassification bias. |
| outcome assessment  *includes blinding, systematic errors, or not comparable outcome measurement across exposure groups.* | considerations:  1) outcome measurements were not influenced by knowledge of the exposure (data were obtained from different databases)  2) validity of disease classification methods (icd coding) | LOW: mortality and morbidity cause classified based on diagnosis standard criteria such as the International Classification System and provided by a National or Regional Database.  PROBABLY LOW: outcome was assessed based on diagnosis standard criteria (ICD) and collected by researcher, but did not specify the data source.  PROBABLY HIGH: outcome was not assessed based on standard diagnosis criteria. Additionally, there is evidence that suggests the existence of misclassification bias.  HIGH: There is evidence that suggests the high risk of misclassification bias. Outcome was assessed based on self-reports. |
| confounding bias | considerations:  1) study appropriately accounted for confounders  2) authors used an appropriate analysis method or study design that accounted for confounding factors. | LOW: study accounted for all important confounders which were measured consistently.  PROBABLY LOW: study accounted for most of confounders AND is not expected to introduce bias.  PROBABLY HIGH: study accounted for some but not all confounders, which is expected to introduce bias  HIGH: study did not account for potential confounders and/or were inappropriately measured |
| other criteria | | |
| selection bias | considerations:  1) participants selected were not done in a manner that might introduce bias in the study | LOW: The descriptions of the studied population were sufficiently detailed to support the assertion that risk of selection effects was minimal.  PROBABLY LOW: There is insufficient information about population selection to permit a judgment of low risk of bias, but there is indirect evidence that suggests low risk of bias  PROBABLY HIGH: There is insufficient information about population selection to permit a judgment of high risk of bias, but there is indirect evidence that suggests high risk of bias  HIGH: There were indications from descriptions of the studied population of high risk of bias |
| incomplete data | considerations:  1) data for outcome measures are not missing, or any missing data is addressed  2) data for exposure measures are not missing, or any missing is addressed | LOW: no missing outcome data or missing data are unrelated to true outcome.  PROBABLY LOW: there was insufficient information about incomplete data to judge for low risk, but indirect evidence that suggests low risk of bias  PROBABLY HIGH: there was insufficient information about incomplete data to judge for high risk, but indirect evidence that suggests high risk bias  HIGH: missing outcome data are related to true outcome and/or there is substantial missing exposure data |
| selective reporting | considerations:  1) the authors report a prior primary and secondary study aims  2) the study reports data analysis over a complete or original database, with no selective reporting of outcomes or analysis | LOW: All of the studies pre-specified outcomes and findings are reported  PROBABLY LOW: there was insufficient information about selective outcome to judge for low risk, but indirect evidence that suggests study was free of selective report  PROBABLY HIGH: there was insufficient information about selective reporting to judge for high risk, but indirect evidence suggests that study was not free of selective reporting  HIGH: not all pre-specified outcomes and findings were reported. |
| conflict of interest | potential source of bias in reporting through source of funding | LOW: the study did not receive funding from an entity with financial interest in the outcome of study  PROBABLY LOW: there is insufficient information to judge for low risk, but indirect evidence suggests study was free of financial interest  PROBABLY HIGH: there is insufficient information to judge for high risk, but indirect evidence suggests study was not free of financial interest  HIGH: study received support from an entity with financial interest in the outcome of study |
| other sources of bias | bias due to other problems not covered elsewhere | LOW: No other sources of bias  PROBABLY LOW: there is insufficient information to judge for low risk, but indirect evidence suggests study was free of other problems  PROBABLY HIGH: Insufficient information to judge for high risk, but indirect evidence suggests study may have had other sources of bias  HIGH: One or more important risk of bias |

## **Section E – Study characteristics**

## **Table E1.** Study characteristics and main findings of all studies assessing the risk of mental health outcomes with high temperature.

| authors | location | climate | population age group of interest | outcomes | methods | period | main findings |
| --- | --- | --- | --- | --- | --- | --- | --- |
| Aydin-Ghormoz, 2022 (5) | New York, USA | humid subtropical (Cfa) | 5–17 years old | hospitalisation with behavioural health disorders and concurrent temperature-related illness | CS | 2005–2019 | overall risk:  1.16 (0.660–2.030) |
| Basu, 2018 (6) | California, USA | range of climates:  warm-summer mediterranean climate (Csb), cold semi-arid climate (BSk), hot-summer mediterranean climate (Csa) | 6–18 years old | emergency room visits for mental health related injuries | TS | 2005–2013 | heat-related risk of emergency department visits for mental health related injuries:  1.073 (1.040–1.108)  cold-related risk estimates: 1.154 (1.136–1.172) |
| Bernstein, 2022 (7) | USA | range of climates | 0–18 years old | emergency room visits for any cause | TS | 2016–2018 | mental disorders and neurodevelopment disorders:  1.20 (1.00–1.50)  suicidality and depression:  1.000 (0.800–1.200) |
| Calkins, 2016 (8) | King County, Washington, USA | humid subtropical (Cfa) | 0–14 years old | calls to emergency medical services | TS | 2007–2012 | risk estimates from two emergency medical services data, including:  basic life support (0–4 years old): 1.680 (0.780–3.600)  basic life support (5–14 years old): 0.990 (0.720–1.340)  advanced life support (0–4 years old): 6.790 (0.820–56.320)  advanced life support (5–14 years old): 0.990 (0.670–1.470) |
| Chan, 2018 (9) | Hong Kong Special Administrative Region | monsoon-influenced humid subtropical climate (Cwa) | <15 years old | mental health-related hospital visits | TS | 2002–2011 | risk was estimated at lag 0–2 days for children < 15:  0.990 (0.670–1.470) |
| Cohen, 2024 (10) | New York, USA | humid subtropical (Cfa) | 0–24 years old | mental health-related hospital visits | TS | 1995–2014 | schizophrenia and other psychotic disorders: 1.491 (1.268–1.758)  mood disorder: 1.15 (0.95–1.39)  anxiety disorders:  1.250 (10.050–1.400)  adjustment disorders: 1.300 (1.200–1.630) |
| Corvetto, 2023 (11) | Curitiba, Brazil | temperate oceanic (Cfb) | 0–17 years old | mental health-related ed visits | TS | 2017–2021 | overall risk:  0.795 (0.530–1.190) |
| Da Silva, 2020 (12) | Curitiba, Paraná, Brazil | temperate oceanic (Cfb) | 0–24 years old | mental health-related hospital visits | TS | 2010–2016 | young men: 1.013 (1.011–1.048)  young women: 1.060 (1.020–1.103) |
| Deng, 2022 (1) | New York, USA | humid subtropical (Cfa) | 0–17 years old | mental health-related ed visits | TS | 2017–2018 | excess risk for each interquartile increase of meteorological factors on emergency department visits for mental disorders for children aged 0–17.  age (0–5 years): 7.500 (-16.800–38.800) age (6–17 years):  3.100 (-0.800–7.200) |
| Doganay, 2003 (13) | Samsun Province, Turkey | humid subtropical (Cfa) | 0–24 years old | suicide | Retrospective | 1996–2001 | seasonal peaks in suicide attempts highest during spring and summer, and among the 15–24-year age group |
| Komulainen, 2022 (14) | Finland | continental subarctic (Dfc) | 10 years old | schizophrenia diagnosis | Cohort | 1990–2017 | hazard exposure ratio:  1.100 (1.080–1.120) |
| Niu, 2024 (15) | Beijing, China | monsoon influenced hot-summer humid continental climate (Dwa) | <18 years old | mental health-related ed visits | Retrospective Cohort | 2016–2018 | overall risk:  2.160 (0.850–9.000) |
| Niu 2023 (2) | New York City, USA | humid subtropical (Cfa) | 6–25 years old | mental health-related ed visits and hospital encounters | CCS | 2005–2011 | 6–11 years old:  1.280 (1.130, 1.460)  12–17 years old:  1.170 (1.090–1.250)  18–25 years old:  1.090 (1.040–1.150) |
| Stowell, 2023 (16) | USA | range of climates | <18 years old | mental health-related ed visits | CCS | 2016–2019 | overall risk:  1.010 (0.930–1.100) |
| Sugg, 2019 (17) | Atlanta  Chicago  New York | Atlanta: humid subtropical (Cfa) Chicago: hot summer humid continental climate (Csa) New York: humid subtropical (Cfa) | Adolescents and young adults | crisis support-seeking calls | TS | 2013–2017 | Atlanta:  1.700 (1.070–2.700)  Chicago:  1.890 (1.370–2.590)  New York:  2.040 (1.730–3.290)  Los Angeles:  1.250 (0.77–1.890) |
| Wang, 2014 (3) | Toronto, Canada | hot summer humid continental climate (Dfa) | 0–14 years old | mental health-related ed visits | TS | 2002–2010 | overall risk:  1.210 (0.730–2.080) |
| Younan, 2018 (18) | Southern California, USA | range of climates: hot summer humid continental climate (Csa)  warm-summer mediterranean climate (Csb) | 9–18 years old | aggressive behaviour |  | 2000–2012 | higher long-term ambient temperatures were linked to increased aggressive behaviours in urban adolescents, with each 1°c rise equating to a 1.5-3-year delay in behavioural maturation |
| Zhang, 2020 (19) | Shenzhen, Zhaoqing & Huizhou, China | range of climates:  monsoon-influenced humid subtropical climate (Cwa)  humid subtropical climate (Cfa) | <18 years old | outpatient visits for mental disorders | TS | 2013–2018 | depressive disorders:  1.040 (0.950–1.140)  anxiety:  0.980 (0.590–1.610)  organic mental disorders: 1.170 (0.480, 2.850)  schizophrenia:  1.080 (0.990–1.170)  affective disorders:  1.040 (0.90–1.210) |
| Zhou, 2023 (20) | Chongqing, China | monsoon-influenced humid subtropical climate (Cwa) | ≤18 years old | outpatient visits for depression | TS | 2014–2019 | overall risk:  0.868 (0.698–1.080) |

**CS**: cross-section, **TS**: time series, **CCS**: case-crossover study; **Cfa** = temperature of warmest month ≥ 10 °C, and temperature of coldest month < 18 °C but > –3 °C; precipitation more evenly distributed throughout year; temperature of warmest month ≥ 22 °C; **Csb** = temperature of warmest month ≥ 10 °C, and temperature of coldest month < 18 °C but > –3 °C; precipitation in driest month of summer half of the year < 30 mm and < ⅓ of wettest month of winter half; temperature of each of four warmest months ≥ 10 °C, but warmest month < 22 °C; **BSk** = ≥ 70% of annual precipitation falls in summer half of year and average annual precipitation total < 20 × (average annual temperature) + 280, or ≥ 70% of annual precipitation falls in winter half of year and average annual precipitation total < 20 × (average annual temperature), or neither half of the year has ≥ 70% of annual precipitation and average annual precipitation total < 20 × (average annual temperature) + 140; average annual precipitation < upper limit for classification as a B type, but > ½ of that amount; average annual temperature < 18 °C; **Csa** = temperature of warmest month ≥ 10 °C, and temperature of coldest month < 18 °C but > –3 °C; precipitation in driest month of summer half of the year < 30 mm and < ⅓ of wettest month of winter half; temperature of warmest month ≥ 22 °C; **Cwa** = temperature of warmest month ≥ 10 °C, and temperature of coldest month < 18 °C but > –3 °C; precipitation in driest month of winter half of year < ⅒ of amount in the wettest month of summer half; temperature of warmest month ≥ 22 °C; **Cfb** = temperature of warmest month ≥ 10 °C, and temperature of coldest month < 18 °C but > –3 °C; precipitation more evenly distributed throughout year; temperature of each of four warmest months ≥ 10 °C, but warmest month < 22 °C; **Dfc** = temperature of warmest month ≥ 10 °C, and temperature of coldest month ≤ –3 °C; precipitation more evenly distributed throughout year; temperature of 1 to 3 months ≥ 10 °C, but warmest month < 22 °C; **Dwa** = temperature of warmest month ≥ 10 °C, and temperature of coldest month ≤ –3 °C; precipitation in driest month of winter half of year < ⅒ of amount in wettest month of summer half; temperature of warmest month ≥ 22 °C; **Dfa** = temperature of warmest month ≥ 10 °C, and temperature of coldest month ≤ –3 °C; precipitation more evenly distributed throughout year; temperature of warmest month ≥ 22 °C

## **Table E2.** Study characteristics and main findings of all studies assessing the risk of mental health outcomes with heatwaves.

| authors | location | climate | population age group of interest | exposure metric | methods | period | main findings  relative risk (95% confidence interval) |
| --- | --- | --- | --- | --- | --- | --- | --- |
| Nitschke, 2007 (21) | Adelaide, Australia | warm-summer mediterranean climate (Csb) | 0–14 years old | ambulance transports, hospital admissions, and mortality | CCS | 1993–2006 | overall risk: 1.080 (1.020–1.140) |
| Nitschke, 2011 (22) | Adelaide, Australia | warm-summer mediterranean climate (Csb) | 0–14 years old | risk estimates for daily hospital admissions | CS | 2008–2009 | 2008 heatwave:  1.090 (0.440–2.700) 2009 heatwave:  0.850 (0.580–1.250) |
| Trang, 2016 (23) | Northern Vietnam | tropical savanna (Aw) | <18 years old | risk for admissions for mental disorders | TS | 2008–2012 | overall risk:  0.060 (0.200–16.710) |
| Sewell, 2024 (24) | North Carolina, USA | humid subtropical (Cfa) | 6–24 years old | mood disorders | observational | 2016–2019 | high-risk cluster: 1.290 (0.680–2.450) low-risk cluster:  1.270 (0.710–2.280) |

**CS**: cross-section, **TS**: time series, **CCS**: case-crossover study; **Csb** = temperature of warmest month ≥ 10 °C, and temperature of coldest month < 18 °C but > –3 °C; precipitation in driest month of summer half of the year < 30 mm and < ⅓ of wettest month of winter half; temperature of each of four warmest months ≥ 10 °C, but warmest month < 22 °C; **Aw** = temperature of coolest month ≥ 18 °C; precipitation in driest month < 60 mm and < 100 – (average annual precipitation total/25); **Cfa** = temperature of warmest month ≥ 10 °C, and temperature of coldest month < 18 °C but > –3 °C; precipitation more evenly distributed throughout year; temperature of warmest month ≥ 22 °C

**Section F — Assessment of risk of bias in individual studies for temperature studies**

**Table F1.** Risk-of-bias assessment of Aydin-Ghormoz et al., 2022.

| #1 Aydin-Ghormoz et al., 2022 | | | |
| --- | --- | --- | --- |
| design | cross section | | |
| participants | aged 5 years and older between 2005–2019 in New York, USA | | |
| exposure | seasonal average | | |
| outcomes | hospitalisation with behavioural health disorders and concurrent temperature-related illness, specifically heat-related illness and cold-related illness | | |
| risk-of-bias Assessment | | | |
| bias domain | | **author’s judgment** | **criterion supportive response** |
| exposure assessment | | low | Data on temperature exposure obtained from regional weather stations across New York State. Temperature records interpolated to cover urban and rural areas, reducing potential misclassification. Authors used daily ambient temperature to link hospital admissions with heat- and cold-related illnesses, ensuring a consistent exposure metric across different regions and periods. |
| outcome assessment | | low | Hospitalisations for heat- and cold-related illness identified using ICD-9 and ICD-10 codes from the New York statewide planning and research cooperative system. Coding done by independent healthcare providers. |
| confounding bias | | low | Study controlled for several confounders, including age, sex, race/ethnicity, and comorbidities (e.g., diabetes, cardiovascular disease, respiratory disease). Additionally, models adjusted for hospital size and location, mitigating potential bias related to socio-economic status or healthcare access. |
| selection bias | | low | Study included all hospitalisations with behavioural health disorders across New York State, capturing a large and diverse population from 2005 to 2019. Inclusion of various demographic groups, including different age ranges, racial and ethnic groups, and insurance statuses, supports representativeness of the sample. |
| incomplete data | | probably low | Statewide Planning and Research Cooperative System dataset provided complete hospitalisation records for the study period. Missing data on comorbidities or insurance status were addressed in sensitivity analyses. |
| selective reporting | | low | All pre-specified outcomes, including hospitalisation rates for heat- and cold-related illness reported. Study followed its analysis plan, and no selective reporting of findings identified. |
| conflict of interest | |  | Funding sources and disclosures do not suggest conflicts of interest. |
| other sources of bias | |  | No indications of other sources of bias. |

**Table F2.** Risk-of-bias assessment of Basu et al., 2018.

| # 2 Basu et al., 2018 | | | |
| --- | --- | --- | --- |
| design | time series | | |
| participants | aged 6 years and older between 2005 to 2013 in California, USA | | |
| exposure | mean apparent temperature | | |
| outcomes | emergency room visits for mental health related injuries | | |
| risk-of-bias assessment | | | |
| bias domain | | **author’s judgment** | **criterion supportive response** |
| exposure assessment | | probably low | Study used apparent temperature (a combination of temperature and humidity) data from 401 meteorological monitors across California, representing 16 climate zones. Population-weighted averages calculated for each zone to estimate exposure. Lagged temperature data help account for short-term effects, but longer-term heat exposure patterns not assessed. |
| outcome assessment | | probably low | Outcomes included mental health-related emergency room visits, classified using ICD-9 codes for various mental health conditions, self-injury/suicide, and inflicted injury/homicide. Records sourced from the California Office of Statewide Health Planning and Development. |
| confounding bias | | probably high | Study adjusted for potential confounders using Poisson regression models and random-effects meta-analysis. However, individual-level factors, such as socio-economic status, medication use, and pre-existing conditions, not directly considered. Authors acknowledged potential confounding effects of socieo-economic factors, but did not address through direct measurement. |
| selection bias | | probably low | Study used emergency room visit data for the entire state of California, representing a large and diverse population. However, analysis excluded individuals treated outside of emergency room settings. |
| incomplete data | | probably high | Study relied on administrative records, which typically provide comprehensive coverage of emergency visits. However, authors noted they could not differentiate between incident and prevalent cases, and study also did not capture emergency room visits outside of State’s public health system. |
| selective reporting | | low | Study reported associations between apparent temperature and broad range of mental health-related outcomes. Analysis stratified results by race/ethnicity, age, and season (warm vs. cold), providing detailed examination of potential effect modification. Both statistically supported and unsupported results presented. |
| conflict of interest | |  | Funding sources and disclosures do not suggest conflicts of interest. |
| other sources of bias | |  | No indications of other sources of bias. |

**Table F3.** Risk-of-bias assessment of Bernstein et al., 2022.

| #3 Bernstein et al., 2022 | | | |
| --- | --- | --- | --- |
| design | time series | | |
| participants | children and adolescents aged 0–18 years in the USA from 2016–2018 | | |
| exposure | daily maximum ambient temperature | | |
| outcomes | emergency department visits for all causes and specific conditions, identified using ICD-10 codes | | |
| risk-of-bias assessment | | | |
| bias domain | | **author’s judgment** | **criterion supportive response** |
| exposure assessment | | probably low | Study used PRISM Climate Group data, which provide gridded, high-resolution daily maximum temperatures (~ 4-km grid). Approach ensures spatial relevance for large geographical areas and minimises misclassification bias. Temperature estimates population-weighted for counties where hospitals located. While PRISM data are robust, they do not account for localised effects, such as urban heat islands, or individual-level exposures |
| outcome assessment | | low | Emergency department visits identified using ICD-10 codes, which are standardised and validated for medical diagnoses. Study used data from the Paediatric Health Information System. |
| confounding bias | | probably high | Study adjusted for confounders, including seasonality, day of the week, temporal trends, and relative humidity. Distributed lag nonlinear models ensure appropriate adjustment for lagged temperature effects, a confounding factor in temperature-health studies. No mention of potential behavioural factors or social determinants. Study did not account for air pollution. |
| selection bias | | probably low | Study included all emergency department visits from 47 children’s hospitals across 27 U.S. states, representing a diverse patient population. However, emergency department visits from non-participating hospitals excluded; approximately 64.2% of participants had public insurance. |
| incomplete data | | probably low | Missing data for outcomes and exposures not explicitly discussed, but large sample size and reliance on the Paediatric Health Information System and PRISM datasets suggest few missing data. Exclusion of hospital days with incomplete temperature or visit data not explicitly described, leaving some uncertainty. No sensitivity analyses reported to account for potential impact of missing data. |
| selective reporting | | low | Study reported outcomes for all pre-specified hypotheses, including all-cause and cause-specific emergency department visits. Subgroup analyses included and transparently reported, enhancing comprehensiveness of findings. |
| conflict of interest | |  | Funding sources and disclosures do not suggest conflicts of interest. |
| other sources of bias | |  | No indications of other sources of bias. |

**Table F4.** Risk-of-bias assessment of Calkins et al., 2016.

| #4 Calkins et al., 2016 | | | |
| --- | --- | --- | --- |
| design | time series | | |
| participants | all residents of King County, Washington from 2007-2012 | | |
| exposure | humidex (temperature and humidity) exposure | | |
| outcomes | number of emergency medical service calls | | |
| risk-of-bias assessment | | | |
| bias domain | | **author’s judgment** | **criterion supportive response** |
| exposure assessment | | probably low | Study used countywide maximum daily humidex (measure of apparent temperature combining heat and humidity) to quantify exposure, with data derived from a detailed meteorological model (PRISM) that accounted for geographic variation across King County. |
| outcome assessment | | probably low | Emergency medical service calls were primary outcome, classified into basic life support and advanced life support categories based on severity of medical condition. The emergency medical service data coded by responders using a unique classification system. |
| confounding bias | | probably high | Study controlled for temporal factors, such as day of the week and yearly trends, using Poisson regression and time-series analyses. However, important individual-level confounders, such as socio-economic status, pre-existing health conditions, and access to healthcare or cooling resources, not available in dataset. |
| selection bias | | probably high | Study included all emergency medical service calls in King County from 2007 to 2012, representing a large and diverse population. However, calls made directly to private ambulance services excluded, potentially causing selection bias, particularly among elderly or institutionalised populations who rely more on private services. Additionally, exclusion of calls with missing age or gender data (~ 20% of total dataset) could result in bias if missing cases disproportionate in certain demographic groups. |
| incomplete data | | probably high | Study used a comprehensive dataset of emergency medical service calls, but excluded calls with missing demographic data, thereby reducing sample size and potentially introducing bias. Sensitivity analysis indicated this exclusion did not affect overall findings. Lack of information on secondary health concerns or specific environmental exposures limits ability to account for individual-level variation in exposure. |
| selective reporting | | low | Study reported results for all pre-specified health conditions and stratified analysis by age group and type of emergency medical service call. Both statistically supported and unsupported results discussed. |
| conflict of interest | |  | Funding sources and disclosures do not suggest conflicts of interest. |
| other sources of bias | |  | No indications of other sources of bias. |

**Table F5.** Risk-of-bias assessment of Chan et al., 2018.

| #5 Chan et al., 2018 | | | |
| --- | --- | --- | --- |
| design | time series | | |
| participants | all residents in Hong Kong Special Administrative Region from 2002 to 2011 | | |
| exposure | daily average temperature | | |
| outcomes | hospital admissions for mental disorders | | |
| risk-of-bias assessment | | | |
| bias domain | | **author’s judgment** | **criterion supportive response** |
| exposure assessment | | high | Study used daily mean temperature recorded by the Hong Kong Observatory, which is centrally located in the city and provides complete temperature data for study period. Choice of a single weather station introduces potential for exposure misclassification because temperatures could vary across different parts of Hong Kong, especially in rural *versus* urban areas. |
| outcome assessment | | low | Outcomes were hospitalisations for mental disorders, identified through ICD-9 codes from the Hong Kong public hospital system, which covers > 99% of hospitalisations in the region. |
| confounding bias | | probably high | Study adjusted for confounders such as day of the week, holiday effects, relative humidity, and air pollution (specifically NO_2_, PM_10_, SO_2_, and O_3_). Confounding effects of air pollutants carefully assessed, and NO_2_ associated with mental disorder hospitalisations, controlled for in final model. However, individual-level confounders, such as socio-economic status, pre-existing mental health conditions, and access to cooling resources, not directly controlled. |
| selection bias | | probably low | Study included all mental disorder hospitalisations in Hong Kong from 2002 to 2011, accounting for > 99% of public hospital admissions. This comprehensive dataset minimises selection bias, because it covers nearly entire population of interest. However, excluded private hospital data and non-hospitalised cases. |
| incomplete data | | probably low | Study appears to have used complete and consistent hospitalisation records from public hospital system, minimising the risk of missing outcome data. |
| selective reporting | | low | Study reported wide range of mental disorders, stratified by disease class, gender, and age group, providing thorough analysis of statistically supported and unsupported findings. |
| conflict of interest | |  | Funding sources and disclosures do not suggest conflicts of interest. |
| other sources of bias | |  | No indications of other sources of bias. |

**Table F6.** Risk-of-bias assessment of Cohen, 2024.

| # 6 Cohen et al., 2024 | | | |
| --- | --- | --- | --- |
| design | time series | | |
| participants | all population in New York State from 1995–2014 | | |
| exposure | daily mean temperature | | |
| outcomes | mental health-related hospital visits | | |
| Risk-of-bias assessment | | | |
| bias domain | | **author’s judgment** | **criterion supportive response** |
| exposure assessment | | low | Study used daily temperature variability, measured as diurnal temperature range, from North American Land Data Assimilation System (NLDAS-2) with fine spatial resolution (~ 11 km × 14 km). |
| outcome assessment | | probably low | Study obtained mental health-related hospital visits from New York State Statewide Planning and Research Cooperative System database, which covers ~ 98% of all hospital visits in the State. However, only hospital visits captured, potentially excluding less severe cases managed in outpatient or primary care settings. |
| confounding bias | | probably high | Study controlled for mean daily temperature and included distributed lag non-linear models to account for time-lagged effects. Additionally, used case-crossover design that controls for time-invariant confounders (e.g., individual characteristics). However, important confounders such as socio-economic status, medication use, and access to cooling resources not accounted for, which could influence both exposure and risk of mental health-related hospital visits. |
| selection bias | | probably low | Study included all hospital visits for mental health-related disorders in New York State, ensuring that sample is representative of population at risk. However, exclusion of private hospital data and non-hospitalised cases, such as those treated in outpatient or emergency settings, could bias selection. |
| incomplete data | | probably low | Study used complete hospitalisation data from Statewide Planning and Research Cooperative System, but potential exposure measurement error exists due to spatial resolution of NLDAS-2 dataset. |
| selective reporting | | low | Study reported associations for all pre-specified outcomes, including hospital visit rates for mood, anxiety, adjustment, and schizophrenia disorders, across various age groups, seasons, and gender. Results presented transparently, and both statistically supported and unsupported findings discussed. |
| conflict of interest | |  | Funding sources and disclosures do not suggest conflicts of interest. |
| other sources of bias | |  | No indications of other sources of bias. |

**Table F7.** Risk-of-bias assessment of Corvetto et al., 2023.

| # 7 Corvetto et al., 2023 | | | |
| --- | --- | --- | --- |
| design | time series | | |
| participants | all populations in Curitiba, Brazil between 2017 and 2021 | | |
| exposure | daily maximum and average temperature | | |
| outcomes | emergency department visits for mental health disorders | | |
| risk-of-bias assessment | | | |
| bias domain | | **author’s judgment** | **criterion supportive response** |
| exposure assessment | | probably low | Study used average daily mean temperatures from 3 weather stations in Curitiba. Although data averaged from multiple stations, capturing broader exposure across city, limitation in representing micro-environmental or individualised exposures. Due to limited station distribution, data interpolation for local temperature variation not possible. |
| outcome assessment | | low | Study used emergency department visits data categorised by ICD-10 codes, including mental health subgroups and suicide attempts, obtained from a public health database. |
| confounding bias | | probably high | Study adjusted for several confounders, including air pollution (ozone and PM_10_), humidity, seasonality, and long-term trends. However, other relevant air pollutants, such as NO_2_, not included due to data limitations and no control for socio-economic factors. |
| selection bias | | probably low | Study included all emergency department visits for mental health across all public centres in Curitiba during study period, minimising likelihood of selection bias. Exclusion limited to non-residents. No exclusion based on age, gender, or socio-economic factors. |
| incomplete data | | probably low | Temperature data imputed where necessary, using correlation-based validation among available weather stations. However, specific percentages of missing data not detailed, and no imputation applied to air pollution data where some missing values occurred. |
| selective reporting | | low | Study reported all relevant mental health subgroups and pre-specified hypotheses, including both statistically supported and unsupported findings. Detailed subgroup analysis done and reported for outcomes. |
| conflict of interest | |  | Funding sources and disclosures do not suggest conflicts of interest. |
| other sources of bias | |  | No indications of other sources of bias. |

**Table F8.** Risk-of-bias assessment of DaSilva et al., 2020.

| #8 DaSilva et al., 2020 | | | |
| --- | --- | --- | --- |
| design | time series | | |
| participants | all populations in Curitiba, Brazil between 2010 and 2017 | | |
| exposure | daily average temperature | | |
| outcomes | hospital admissions for mental and behavioural disorders | | |
| risk-of-bias assessment | | | |
| bias domain | | **author’s judgment** | **criterion supportive response** |
| exposure assessment | | low | Study used data from 3 urban air quality monitoring stations in Curitiba to measure daily average temperature, relative humidity, and air pollutants (SO_2_, NO_2_, O_3_, PM_10_) over 6 years. Study did not apply gridded or population-weighted temperature data. Meteorological data consistent and likely representative of urban population, but absence of individual-level exposure data slightly elevates risk of misclassification |
| outcome assessment | | low | Outcome (hospital admissions for mental and behavioural disorders) assessed using International Classification of Diseases (ICD-10) coding system, a standard and reliable classification system for medical diagnoses. Data obtained from the public Single System of Health, a national database. |
| confounding bias | | probably low | Study controlled for environmental confounders, including relative humidity, day of the week, time trends and air pollutants in the model. However, individual-level socio-economic and health-related confounders not considered. Distributed lag non-linear model and generalised additive model strengthen analysis. |
| selection bias | | low | Study included all hospital admissions for mental and behavioural disorders in Curitiba from 2010 to 2016, minimising risk of selection bias. Large, representative sample across different population subgroups (e.g., age, gender) and all seasons suggest results unlikely to be biased by selection. |
| incomplete data | | probably low | While study did not explicitly address missing data, air-quality stations used for exposure assessment had ≥ 80% valid data, and meteorological and hospitalisation data appeared comprehensive. However, study does not specify how missing hospital admissions data, if any, handled. |
| selective reporting | | low | Study reports findings across multiple age groups, genders, and exposures, including both statistically supported and unsupported results. Full range of environmental variables discussed, and all pre-specified outcomes presented. |
| conflict of interest | |  | Funding sources and disclosures do not suggest conflicts of interest. |
| other sources of bias | |  | No indications of other sources of bias. |

**Table F9.** Risk-of-bias assessment of Deng et al., 2022.

| #9 Deng et al., 2022 | | | |
| --- | --- | --- | --- |
| design | time series | | |
| participants | all population in New York State from 2017–2018 | | |
| exposure | daily temperature data and heat index | | |
| outcomes | mental disorder-related emergency department visits | | |
| risk-of-bias assessment | | | |
| bias domain | | **author’s judgment** | **criterion supportive response** |
| exposure assessment | | low | Study used high-resolution weather monitoring system, New York State Mesonet, to collect data on multiple meteorological factors, including solar radiation, temperature, relative humidity, heat index, and rainfall. |
| outcome assessment | | probably low | Mental disorder-related emergency department visits identified from New York State Statewide Planning and Research Cooperative System database using ICD-10 codes. Database covers 95% of hospitals in the State, ensuring nearly comprehensive coverage of population. |
| confounding bias | | probably high | Study employed time-stratified case-crossover design, which controls for time-invariant confounders such as age, gender, and race. Additionally, study adjusted for air pollutants, holidays, and time trends in conditional logistic regression models. However, individual-level confounders, such as socio-economic status and personal activity patterns not recorded or controlled. |
| selection bias | | probably low | Study included all emergency department visits for mental disorders from nearly all hospitals in New York State. This broad coverage minimises risk of selection bias related to geographic or demographic factors. However, private hospitals and non-hospitalised cases excluded. |
| incomplete data | | probably low | Dense network of weather monitoring stations and comprehensive hospital database suggest both exposure and outcome data relatively complete. |
| selective reporting | | low | Study reported associations between multiple meteorological factors (solar radiation, temperature, relative humidty, heat index, rainfall) and emergency department visits for mental disorders across various subgroups (e.g., demographic groups, mental disorder subtypes). Both statistically supported and unsupported findings were, and results stratified by month to highlight seasonal variation. |
| conflict of interest | |  | Funding sources and disclosures do not suggest conflicts of interest. |
| other sources of bias | |  | No indications of other sources of bias. |

**Table F10.** Risk-of-bias assessment of Doganay et al., 2003.

| #10 Doganay et al., 2003 | | | |
| --- | --- | --- | --- |
| design | time series | | |
| participants | all population in Samsun Province, Turkey from1996 - 2001 | | |
| exposure | monthly maximum and average temperature | | |
| outcomes | suicide | | |
| risk-of-bias assessment | | | |
| bias domain | | **author’s judgment** | **criterion supportive response** |
| exposure assessment | | probably low | Study used meteorological data from Regional Meteorological Institute of Samsun, Turkey, to examine associations between weather variables (temperature, humidity, duration and intensity of sunlight, cloudiness, and atmospheric pressure) and suicide attempts. |
| outcome assessment | | probably low | Study obtained data on suicide attempts from hospital records at Ondokuz Mayis University Hospital between 1996 and 2001, with suicide attempts identified using ICD-9 codes. All cases reviewed by a psychiatrist to confirm psychiatric diagnoses. However, only included hospital-treated cases, potentially missing less severe attempts that did not result in hospitalisation. |
| confounding bias | | high | Study did not extensively control for individual-level confounders, such as socio-economic status, pre-existing mental health conditions, or substance use. |
| selection bias | | probably high | Study included all suicide attempts admitted to a single hospital (Ondokuz Mayis University Hospital), which reduces potential for selection bias within that hospital’s population. However, single hospital limits generalisability of findings to broader population. |
| incomplete data | | probably low | Study does not mention missing data related to hospital records or meteorological data, suggesting data completeness likely not a major problem. However, no indication of whether all suicide attempts in the region captured. |
| selective reporting | | low | Study reported association between suicide attempts and various weather conditions, with results stratified by age, sex, and season. Both statistically supported and unsupported findings presented, and analysis of different types of suicide attempts (violent *vs*. non-violent) adds depth to report. |
| conflict of interest | |  | Funding sources and disclosures do not suggest conflicts of interest. |
| other sources of bias | |  | No indications of other sources of bias. |

**Table F11.** Risk-of-bias assessment of Komulainen et al., 2022.

| #11 Komulainen et al., 2022 | | | |
| --- | --- | --- | --- |
| design | cohort | | |
| participants | all population born between 1990 and 1995 in Finland | | |
| exposure | ambient mean temperature (°C), calculated as 10-year cumulative exposure from birth to 10^th^ birthday | | |
| outcomes | schizophrenia diagnosis | | |
| Risk-of-bias assessment | | | |
| bias domain | | **author’s judgment** | **criterion supportive response** |
| exposure assessment | | probably low | Study used gridded daily climatic data, including solar radiation and ambient temperature, from Finnish Meteorological Institute. Data linked to participants’ residential postal code, providing spatial accuracy. However, individual-level exposure not captured. Gridded climate data improve reliability, but do not eliminate risk of exposure misclassification. |
| outcome assessment | | low | Study assessed schizophrenia onset using ICD-10 codes from Finnish Care Register for Health Care. |
| confounding bias | | probably low | Study controlled for wide range of important confounders, including parental mental disorders, parental education, income, urbanicity, and area-level socio-economic characteristics. However, analysis did not account for other potential environmental confounders such as air pollution, which could influence mental health independently of climatic exposure. |
| selection bias | | low | Study included all individuals born in Finland between 1990 and 1995, with comprehensive follow-up until age of 27 or until first schizophrenia diagnosis. Large, population-based cohort not restricted by demographic or geographic factors. |
| incomplete data | | low | Study did not report issues with missing data. National health and meteorological databases ensure comprehensive data coverage. No indication that incomplete data could have biased results. Data handling from birth cohorts and nationwide registries further supports confidence in data completeness. |
| selective reporting | | low | Study presented results for all pre-specified analyses, including statistically supported and unsupported findings. Secondary analyses also done to assess continuous exposure-response relationship, and these results transparently reported |
| conflict of interest | |  | Funding sources and disclosures do not suggest conflicts of interest. |
| other sources of bias | |  | No indications of other sources of bias. |

**Table F12.** Risk-of-bias assessment of Niu et al., 2024.

| # 12 Niu et al., 2024 | | | |
| --- | --- | --- | --- |
| design | retrospective cohort | | |
| participants | all population in Beijing, China from 2016 to 2018 | | |
| exposure | daily mean temperature | | |
| outcomes | emergency department visits for mental disorders | | |
| risk-of-bias assessment | | | |
| bias domain | | **author’s judgment** | **criterion supportive response** |
| exposure assessment | | low | Study used daily temperature data from China Meteorological Data Service Centre, as well as data on relative humidity, sunshine duration, and precipitation. Projected temperature data derived from 26 general circulation models under four climate change scenarios. |
| outcome assessment | | probably low | Study used hospital emergency department visit records for mental disorders from 30 hospitals in Beijing, covering 34% of all emergency visits in city during study period. Mental disorders classified using ICD-10 codes. |
| confounding bias | | probably high | Study controlled for several confounders, including relative humidity, sunshine duration, precipitation, and air pollutants using a quasi-Poisson model and distributed lag nonlinear model. However, individual-level confounders, such as socio-economic status, access to mental healthcare, and pre-existing health conditions, were not controlled. |
| selection bias | | probably high | Study included all emergency department visits from 30 hospitals in Beijing, covering ~ 34% of total visits during study period. Exclusion of data from private hospitals and outpatient settings could introduce selection bias. |
| incomplete data | | probably low | Meteorological data and hospital records appear to complete, and study employed rigorous methods to project future temperature-related health outcomes. However, study did not account for potential future demographic shifts or changes in healthcare practices. |
| selective reporting | | low | Study reported range of mental health outcomes, including visits for psychoactive substance use, schizophrenia, and mood disorders, and stratified analysis by sex and age. Both statistically supported and unsupported results presented, and analysis appears thorough and transparent. |
| conflict of interest | |  | Funding sources and disclosures do not suggest conflicts of interest. |
| other sources of bias | |  | No indications of other sources of bias. |

**Table F13.** Risk-of-bias assessment of Niu et al., 2023.

| #13 Niu et al., 2023 | | | |
| --- | --- | --- | --- |
| design | case-crossover study | | |
| participants | 6–25 years olds, New York City, USA; 2005–2011 | | |
| exposure | daily minimum temperature | | |
| outcomes | mental health-related ed visits and hospital encounters | | |
| risk-of-bias assessment | | | |
| bias domain | | **author’s judgment** | **criterion supportive response** |
| exposure assessment | | low | Study used daily minimum temperature data from four meteorological stations in New York City to examine association between temperature and mental health-related healthcare encounters. |
| outcome assessment | | probably low | Study used data from New York State Statewide Planning and Research Cooperative System database, which includes comprehensive information on emergency department visits and hospital admissions for mental health disorders, covering large proportion of hospitalisations in New York City. ICD-9 codes for classifying mental health diagnoses ensures standardised outcome identification. However, study only captured severe cases that resulted in hospitalisation or emergency department visits, excluding outpatient or non-emergency cases. Additionally, study unable to control for potential biases in mental health diagnostic patterns, particularly those across racial or socio-economic subgroups. |
| confounding bias | | probably high | Study controlled for several environmental confounders, including relative humidity and air pollution (PM_2.5_, SO_2_, and O_3_), using case-crossover design, which adjusts for time-invariant individual characteristics. However, individual-level confounders, such as socio-economic status, access to mental healthcare, pre-existing mental health conditions, and medication use, not controlled. |
| selection bias | | probably low | Study included large, representative sample of emergency department visits and hospital admissions for mental health disorders from multiple hospitals across New York City. However, excluded patients treated in private hospitals or outpatient settings. |
| incomplete data | | low | Study used complete meteorological and hospital data over several years, with no indication of missing data. |
| selective reporting | | low | Study reported results for multiple subgroups (age, sex, race/ethnicity, and payment source), and both statistically supported and unsupported findings presented. |
| conflict of interest | |  | Funding sources and disclosures do not suggest conflicts of interest. |
| other sources of bias | |  | No indications of other sources of bias. |

**Table F14.** Risk-of-bias assessment of Stowell et al., 2023.

| #14 Stowell et al., 2023 | | | |
| --- | --- | --- | --- |
| design | cohort study | | |
| participants | aged ≥ 5 years from 2005–2019 in New York, USA | | |
| exposure | seasonal average | | |
| outcomes | hospitalisation with behavioural health disorders and concurrent temperature-related illness, specifically heat- and cold-related illness | | |
| risk-of-bias assessment | | | |
| bias domain | | **author’s judgment** | **criterion supportive response** |
| exposure assessment | | low | Study publicly available, high-resolution gridded climate dataset. Temperature estimates population-weighted to represent exposures at county level, ensuring spatial relevance. Daily maximum ambient temperature calculated and aggregated to county level using census data. |
| outcome assessment | | low | Outcome data obtained from medical claims records using ICD-10 codes. No evidence that outcome measurements influenced by knowledge of exposure. Data extracted from a national database. |
| confounding bias | | low | Study used case-crossover design, which controls for time-invariant confounders, such as age, sex, and socio-economic status. Models adjusted for confounders, including relative humidity, seasonality, and holidays. Case period compared to control periods within same month, year, and day of the week to address temporal confounding. |
| selection bias | | probably high | Study included children with commercial health insurance across 2489 counties, representing 97% of U.S. population < 18 years. However, children without commercial insurance (e.g., those on public insurance or uninsured) excluded. |
| incomplete data | | probably low | County-days with > 20% missing data excluded, affecting < 0.01% of dataset. Study does not report major issues with missing data for outcomes or exposures. |
| selective reporting | | low | Study analysed all-cause and cause-specific emergency department visits, as outlined in objectives. Results presented for all main outcomes, including subgroup analyses |
| conflict of interest | | none | Funding sources and disclosures do not suggest conflicts of interest. |
| other sources of bias | | none | No indications of other sources of bias. |

**Table F15.** Risk-of-bias assessment of Sugg et al., 2019.

| # 15 Sugg et al., 2019 | | | |
| --- | --- | --- | --- |
| design | time series | | |
| participants | adolescents and young adults | | |
| exposure | daily minimum and maximum temperature | | |
| outcomes | number of crisis support-seeking calls | | |
| risk-of-bias assessment | | | |
| bias domain | | **author’s judgment** | **criterion supportive response** |
| exposure assessment | | probably low | Study used meteorological data from North Carolina Climate Office to assess the relationship between temperature and crisis support-seeking behaviour among young adults and adolescents. Exposure measure was daily maximum temperature for the region, averaged across multiple weather stations. |
| outcome assessment | | probably low | Outcome measure was volume of crisis text line messages from young adults and adolescents in response to mental health issues. Data gathered from a national crisis text line service. |
| confounding bias | | probably high | Study accounted for day of the week, month, and other temporal factors, such as holidays, using generalised additive models. However, individual-level confounders, such as socio-economic status, access to mental health services or prior mental health diagnoses not controlled. |
| selection bias | | probably high | Study included data from large national crisis text line, which serves a wide demographic of young adults and adolescents. This minimises risk of selection bias related to the type of service used, because service is available 24/7 and widely accessible. National service ensures a large, representative sample, but outcome misclassification could occur because data only capture individuals who actively seek help through this service. |
| incomplete data | | low | Study used a comprehensive dataset from crisis text line and publicly available meteorological data. |
| selective reporting | | low | Study presented results for different temperature ranges and stratified them by time of day and day of the week. Both statistically supported and unsupported findings were reported, and the results discussed transparently. |
| conflict of interest | |  | Funding sources and disclosures do not suggest conflicts of interest. |
| other sources of bias | |  | No indications of other sources of bias. |

**Table F16.** Risk-of-bias assessment of Wang et al., 2014.

| # 16 Wang et al., 2014 | | | |
| --- | --- | --- | --- |
| design | time series | | |
| participants | all population in Toronto, Canada from 2002 to 2010 | | |
| exposure | mean temperature | | |
| outcomes | emergency room visits related to mental and behavioural disorders | | |
| risk-of-bias assessment | | | |
| bias domain | | **author’s judgment** | **criterion supportive response** |
| exposure assessment | | probably high | Study used daily mean temperature data from Environment Canada weather station at Toronto Pearson International Airport to examine association between ambient temperature and emergency room admissions for mental and behavioural disorders. |
| outcome assessment | | low | Study used data from National Ambulatory Care Reporting System, which covers > 97% of emergency room visits in Ontario, providing a comprehensive dataset. Mental and behavioural disorders classified using ICD-10 codes, ensuring consistent outcome identification. |
| confounding bias | | probably high | Study controlled for several confounders, including day of the week, air pollution (NO_2_, CO, and O_3_), humidity, and seasonality, using a distributed lag nonlinear model. However, individual-level confounders, such as socio-economic status, access to healthcare, pre-existing health conditions, and medication use, were not controlled. |
| selection bias | | probably low | Study included nearly all emergency room visits in Toronto, minimising risk of selection bias due to missing data from hospitals. However, study excluded patients who did not seek emergency room care or who were treated in outpatient settings. |
| incomplete data | | probably low | Weather and emergency room data comprehensive for study period (2002–2010), but study lacked individual-level data, such as specific medication use, or socio-economic factors. Additionally, study did not include data on non-hospitalised mental health crises. |
| selective reporting | | low | Study reported wide range of mental health outcomes, including schizophrenia, mood disorders, neurotic disorders, and substance abuse, with both statistically supported and unsupported findings presented. |
| conflict of interest | |  | Funding sources and disclosures do not suggest conflicts of interest. |
| other sources of bias | |  | No indications of other sources of bias. |

**Table F17.** Risk-of-bias assessment of Younan et al., 2018.

| # 17 Younan et al., 2018 | | | |
| --- | --- | --- | --- |
| design | longitudinal | | |
| participants | southern California, USA, 9–18 years; 2000–2012 | | |
| exposure | average ambient temperatures over 1, 2, and 3 years | | |
| outcomes | aggressive behaviour | | |
| risk-of-bias assessment | | | |
| bias domain | | **author’s judgment** | **criterion supportive response** |
| exposure assessment | | low | Study used hourly meteorological data obtained from California Air Resources Board’s Air Quality and Meteorological Information System. Temperature data assigned to each geocoded residential address based on proximity to nearest weather station, providing detailed spatial resolution for ambient temperature exposure. Study aggregated temperatures for the 1, 2, and 3 years preceding behavioural assessments. |
| outcome assessment | | high | Outcome measures aggressive and delinquent behaviours assessed using parent-reported version of the Child Behaviour Checklist. However, relies on subjective reporting. |
| confounding bias | | probably low | Study controlled for several important confounders, including age, sex, ethnicity, socio-economic status, and neighbourhood characteristics. Covariates selected using a directed acyclic graph to ensure comprehensive approach to controlling confounding variables. However, study did not adjust for individual-level confounders, such as pre-existing mental health conditions, family dynamics, or access to mental health services. |
| selection bias | | probably low | Study included participants from ongoing Risk Factors for Antisocial Behaviour twin study, reflecting socio-economic diversity of greater Los Angeles area. However, selection bias could arise due to exclusion of individuals without complete behavioural assessments or geocoded residential addresses. |
| incomplete data | | low | Meteorological and behavioural data comprehensive, and study did sensitivity analyses to account for potential confounders. |
| selective reporting | | low | Study reported results for multiple time periods (1-, 2-, and 3-year temperature exposures) and included both statistically supported and unsupported findings. Authors did extensive sensitivity analyses and presented results for subgroups based on sex, socio-economic status, and neighbourhood green space, providing a comprehensive view of data. |
| conflict of interest | |  | Funding sources and disclosures do not suggest conflicts of interest. |
| other sources of bias | |  | No indications of other sources of bias. |

**Table F18.** Risk-of-bias assessment of Zhang et al., 2020.

| # 18 Zhang et al., 2020 | | | |
| --- | --- | --- | --- |
| design | time series | | |
| participants | all populations in Shenzhen, Zhaoqing, and Huizhou, China between 2013 and 2018 | | |
| exposure | exposure to extreme cold effects (2.5^th^ percentile) and hot effects (97.5^th^ percentile) | | |
| outcomes | outpatient visits for mental disorders | | |
| risk-of-bias assessment | | | |
| bias domain | | **author’s judgment** | **criterion supportive response** |
| exposure assessment | | probably low | Study used daily average ambient temperature data from multiple monitoring stations across Shenzhen, Zhaoqing, and Huizhou, providing good coverage and mitigating potential exposure misclassification. Data collected from central meteorological source, enhancing consistency. However, some exposure misclassification can still arise due to differences in individual exposures within various microenvironments not captured. |
| outcome assessment | | low | Study used mental health outpatient visit data from local psychiatric hospitals, with diagnoses based on ICD-10 coding standards, ensuring outcome reliability and comparability. |
| confounding bias | | low | Study employed time-stratified case-crossover design, which controls for confounders, individual characteristics and temporal trends. Including additional adjustments for air pollutants (PM_2.5_, O_3_, NO_2_, SO_2_) and relative humidity further minimised potential confounding effects. Additionally, study used distributed lag nonlinear model. |
| selection bias | | low | Study included all outpatient visits for mental disorders across three cities in Guangdong Province, encompassing broad and representative sample. By covering diverse geographic region without exclusions, study likely minimised selection bias. |
| incomplete data | | low | No direct information on missing data; study did not report any major gaps in health or meteorological records. |
| selective reporting | | low | Study appears comprehensive in reporting associations between temperature extremes and various mental health conditions, including both statistically supported and unsupported findings. |
| conflict of interest | |  | Funding sources and disclosures do not suggest conflicts of interest. |
| other sources of bias | |  | No indications of other sources of bias. |

**Table F19.** Risk-of-bias assessment of Zhou et al., 2023.

| # 19 Zhou et al., 2023 | | | |
| --- | --- | --- | --- |
| design | time series | | |
| participants | all populations in Chongqing, China between 2014 and 2019 | | |
| exposure | humidex | | |
| outcomes | outpatient visits for depression | | |
| risk-of-bias assessment | | | |
| bias domain | | **author’s judgment** | **criterion supportive response** |
| exposure assessment | | probably low | Study assessed exposure using multiple meteorological data, including 28 monitoring stations across Chongqing to cover urban areas. Humidex values, combining temperature and humidity, calculated based on standardised methods. However, relying on ambient data without individual-level exposure details does not capture specific microenvironments. |
| outcome assessment | | low | Depression outpatient visits tracked using hospital records in Chongqing’s tertiary hospitals, providing reliable source of clinical data. Study used International Classification of Diseases codes to ensure standardised outcome measurement across visits. |
| confounding bias | | probably high | Study employed distributed lag nonlinear models and accounted for confounding variables like air pollutants (PM_2.5_ and O_3_), calendar time, day of the week, and meteorological factors such as rainfall and wind velocity. Individual factors such as socio-economic status and detailed personal health history not included. |
| selection bias | | probably low | Study included many depression outpatient records from two main tertiary hospitals that serve Chongqing’s urban population. While comprehensive, there is slight risk of selection bias, because data specific to hospital-based outpatient visits. |
| incomplete data | | low | Data completeness high, with no missing values reported for both meteorological exposure and outpatient depression visit data. Study used comprehensive dataset for daily monitoring across region, and no indication that incomplete records impacted results. |
| selective reporting | | low | Study presents findings transparently, including effect estimates across different lags and subgroups. All statistically supported and unsupported results discussed. |
| conflict of interest | |  | Funding sources and disclosures do not suggest conflicts of interest. |
| other sources of bias | |  | No indications of other sources of bias. |

**Figure F1:** Risk assessment of high temperature studies as per the 4-point scale of the Office of Health Assessment and Translation tool.

| **study** | **exposure** | **outcome** | **confounding** | **selection** | **incomplete data** | **selective reporting** |
| --- | --- | --- | --- | --- | --- | --- |
| **Aydin-Ghormoz et al., 2022** | low risk | low risk | low risk | low risk | probably low risk | low risk |
| **Basu et al.,2018** | probably low risk | probably low risk | probably high risk | probably low risk | probably high risk | low risk |
| **Bernstein et al., 2022** | probably low risk | low risk | probably high risk | probably low risk | probably low risk | low risk |
| **Calkins et al.,2016** | probably low risk | probably low risk | probably high risk | probably high risk | probably high risk | low risk |
| **Chan et al., 2018** | high risk | low risk | probably high risk | probably low risk | probably low risk | low risk |
| **Cohen et al.,2024** | low risk | probably low risk | probably high risk | probably low risk | probably low risk | low risk |
| **Corvetto et al, 2023** | probably low risk | low risk | probably high risk | probably low risk | probably low risk | low risk |
| **Dasilva et al.,2020** | low risk | low risk | probably low risk | low risk | probably low risk | low risk |
| **Deng et al., 2022** | low risk | probably low risk | probably high risk | probably low risk | probably low risk | low risk |
| **Doganay et a., 2003** | probably low risk | probably low risk | high risk | probably high risk | probably low risk | low risk |
| **Komulainen et al., 2022** | probably low risk | low risk | probably low risk | low risk | low risk | low risk |
| **Niu et al., 2023** | low risk | probably low risk | probably high risk | probably low risk | low risk | low risk |
| **Niu et al., 2024** | low risk | probably low risk | probably high risk | probably high risk | probably low risk | low risk |
| **Stowell et al., 2023** | low risk | low risk | low risk | high risk | probably low risk | low risk |
| **Sugg et al., 2019** | probably low risk | probably low risk | probably high risk | probably high risk | low risk | low risk |
| **Wang et al.,2014** | probably high risk | low risk | probably high risk | probably low risk | probably low risk | low risk |
| **Younan et al., 2018** | low risk | high risk | probably low risk | probably low risk | low risk | low risk |
| **Zhang et al., 2020** | probably low risk | low risk | probably high risk | probably low risk | low risk | low risk |
| **Zhou et al., 2023** | probably low risk | low risk | low risk | low risk | low risk | low risk |

**Section G – Assessment of risk of bias in individual studies for heatwave studies**

**Table G1.** Risk-of-bias assessment of Nitschke et al., 2007.

| # 1 Nitschke et al., 2007 | | | |
| --- | --- | --- | --- |
| design | case-crossover study | | |
| participants | all populations in Adelaide, Australia between 1993 to 2006 | | |
| exposure | heatwaves | | |
| outcomes | ambulance transports, hospital admissions, and mortality | | |
| risk-of-bias assessment | | | |
| bias domain | | **author’s judgment** | **criterion supportive response** |
| exposure assessment | | probably high | Study used daily maximum temperature data from single weather station in Adelaide. While Kent Town weather station is representative of metropolitan area’s climate, relying on a single station limits spatial coverage and could result in some exposure misclassification, especially in regions with distinct microclimates. Study’s heatwave definition (≥ 35°C for ≥ 3 consecutive days) appropriate for context. |
| outcome assessment | | low | Study collected hospital admissions, ambulance transport, and mortality data from well-established sources using the ICD-9 and ICD-10 classification systems. Data obtained from public health databases, which are reliable and consistent. |
| confounding bias | | probably high | Study controlled for seasonal variation and long-term time trends using Poisson regression models. However, did not account for important potential confounders like air pollution or individual-level socio-economic status, which could affect both exposure (heatwaves) and outcomes (morbidity and mortality). |
| selection bias | | low | Study included all ambulance callouts, hospital admissions, and mortality data from the metropolitan Adelaide population during heatwaves and non-heatwave periods. Broad inclusion of all relevant cases minimises risk of selection bias, because no exclusion criteria based on age, sex, or socio-economic factors applied. |
| incomplete data | | probably low | Study does not explicitly mention how missing data handled, particularly for ambulance and hospital records. While datasets used generally reliable and comprehensive, lack of detailed information on data completeness introduces uncertainty. |
| selective reporting | | low | Study reports all pre-specified health outcomes (ambulance callouts, hospital admissions, and mortality), with findings presented for both statistically supported and unsupported results across various health conditions. |
| conflict of interest | |  | Funding sources and disclosures do not suggest conflicts of interest. |
| other sources of bias | |  | No indications of other sources of bias. |

**Table G2.** Risk-of-bias assessment of Nitschke, 2011.

| # 2 Nitschke et al., 2011 | | | |
| --- | --- | --- | --- |
| design | cohort study | | |
| participants | all populations in Adelaide, Australia from 2008–2009 | | |
| exposure | heatwaves | | |
| outcomes | morbidity and mortality related to heatwaves | | |
| risk-of-bias assessment | | | |
| bias domain | | **author’s judgment** | **criterion supportive response** |
| exposure assessment | | probably high | Study measured ambient temperature using single weather station in Adelaide, which recorded daily maximum temperatures. While the station described as representative of Adelaide metropolitan area, a single station introduces potential for exposure misclassification, because temperature can vary across different parts of city. Heatwave definition (3 consecutive days with temperatures ≥ 35°C) is appropriate |
| outcome assessment | | low | Study collected morbidity and mortality data from hospital admissions, ambulance callouts, and emergency department presentations using ICD-9 and ICD-10 codes. |
| confounding bias | | probably high | Study adjusted for important confounders, such as seasonality and day of the week, and included a time-stratified analysis to reduce bias from time-invariant confounders. However, other potential confounders, such as air pollution or socio-economic factors, not fully controlled. |
| selection bias | | probably low | Study included all ambulance callouts, hospital admissions, and emergency department visits during heatwave periods and compared them to non-heatwave periods. No exclusion criteria applied based on demographic factors. |
| incomplete data | | probably low | Study did not explicitly mention missing data issues, and used well-established hospital and emergency service databases. |
| selective reporting | | low | Study reports all health outcomes it set out to measure, including ambulance callouts, hospital admissions, and mortality across various health conditions. Both statistically supported and unsupported findings are transparently reported. |
| conflict of interest | |  | Funding sources and disclosures do not suggest conflicts of interest. |
| other sources of bias | |  | No indications of other sources of bias. |

**Table G3.** Risk-of-bias assessment of Sewell et al., 2024.

| # 3 Sewell et al., 2024 | | | |
| --- | --- | --- | --- |
| design | observational | | |
| participants | 6–24 years in Northern Carolina, USA between 2016–2019 | | |
| exposure | exposure to three separate climate stressors (including heatwaves) | | |
| outcomes | suicide and mood disorder | | |
| risk-of-bias assessment | | | |
| bias domain | | **author’s judgment** | **criterion supportive response** |
| exposure assessment | | low | Study uses daily ambient temperature data obtained from the Finnish Meteorological Institute, with daily recordings linked to each individual's residential postal code area (10 km × 10 km grid data). Study used temperature and solar radiation measurements across a large area, coupled with a 10-year exposure window. |
| outcome assessment | | low | Outcome (schizophrenia diagnosis) assessed using ICD-10 codes from Care Register for Health Care. |
| confounding bias | | low | Study controls for several important confounders, including sex, birth year, parental history of mental disorders, and socio-economic characteristics such as parental education and income. However, limited information on how seasonal variation and temporal trends in environmental conditions handled. |
| selection bias | | low | Study included all children born in Finland between 1990 and 1995 who were alive and living in Finland until 10^th^ birthday. Population-based cohort minimises risk of selection bias because includes a comprehensive sample, ensuring equal opportunity for participants across different exposures. |
| incomplete data | | low | No indication of missing data that would affect study’s validity. |
| selective reporting | | low | Study reports all pre-specified outcomes, including schizophrenia diagnoses and climatic exposure. Does not appear to report results selectively or withhold findings. |
| conflict of interest | |  | Funding sources and disclosures do not suggest conflicts of interest. |
| other sources of bias | |  | No indications of other sources of bias. |

**Table G4.** Risk-of-bias assessment of Trang, 2016.

| # 4 Trang et al., 2016 | | | |
| --- | --- | --- | --- |
| design | time series | | |
| participants | all populations in northern Vietnam between 2008–2012 | | |
| exposure | heatwaves | | |
| outcomes | risk for admissions for mental disorders | | |
| risk-of-bias assessment | | | |
| bias domain | | **author’s judgment** | **criterion supportive response** |
| exposure assessment | | low | Study used meteorological data from several monitoring stations in Hanoi to estimate daily maximum temperatures and applied 90^th^ percentile (35 °C) to define heatwaves. |
| outcome assessment | | low | Study used hospital admissions data for mental disorders from Hanoi Mental Hospital, with diagnoses classified according to International Classification of Diseases (ICD-10). Reliance on centralised, established hospital system and standardised diagnostic codes minimised risk of outcome misclassification |
| confounding bias | | probably high | Study controlled for several important confounders, including seasonality, time trends, and humidity. However, did not include adjustments for air pollution, socio-economic status, or pre-existing health conditions, which could have influenced outcomes independently of heat exposure. |
| selection bias | | probably high | Study included all hospital admissions for mental disorders during the study period at a major mental health hospital in Hanoi, but it relied on data from a single hospital. This could limit the generalisability of findings to broader population, particularly those who sought care at other facilities or not admitted to the hospital. |
| incomplete data | | probably low | Study did not report problems with missing data, but also did not explicitly address how incomplete data, if present, handled. |
| selective reporting | | low | Study transparently reported results for all pre-specified hypotheses, including effects of heatwaves on different subgroups. Both statistically supported and unsupported results presented |
| conflict of interest | |  | Funding sources and disclosures do not suggest conflicts of interest. |
| other sources of bias | |  | No indications of other sources of bias. |

**Figure G1:** Risk assessment of heatwave studies as per the 4-point scale of the Office of Health Assessment and Translation tool.

| **study** | **exposure** | **outcome** | **confounding** | **selection** | **incomplete data** | **selective reporting** |
| --- | --- | --- | --- | --- | --- | --- |
| **Nitschke et al., 2011** | probably high risk | low risk | probably high risk | probably low risk | probably low risk | low risk |
| **Nitschke et al., 2007** | probably high risk | low risk | probably high risk | low risk | probably low risk | low risk |
| **Sewell et al., 2024** | low risk | low risk | low risk | low risk | low risk | low risk |
| **Trang et al., 2016** | low risk | low risk | probably high risk | probably high risk | probably low risk | low risk |

**Section H – Results for temperature studies**

**Figure H1.** Forest plot of meta-analytical results for temperature studies with prediction intervals (*n* = 14)


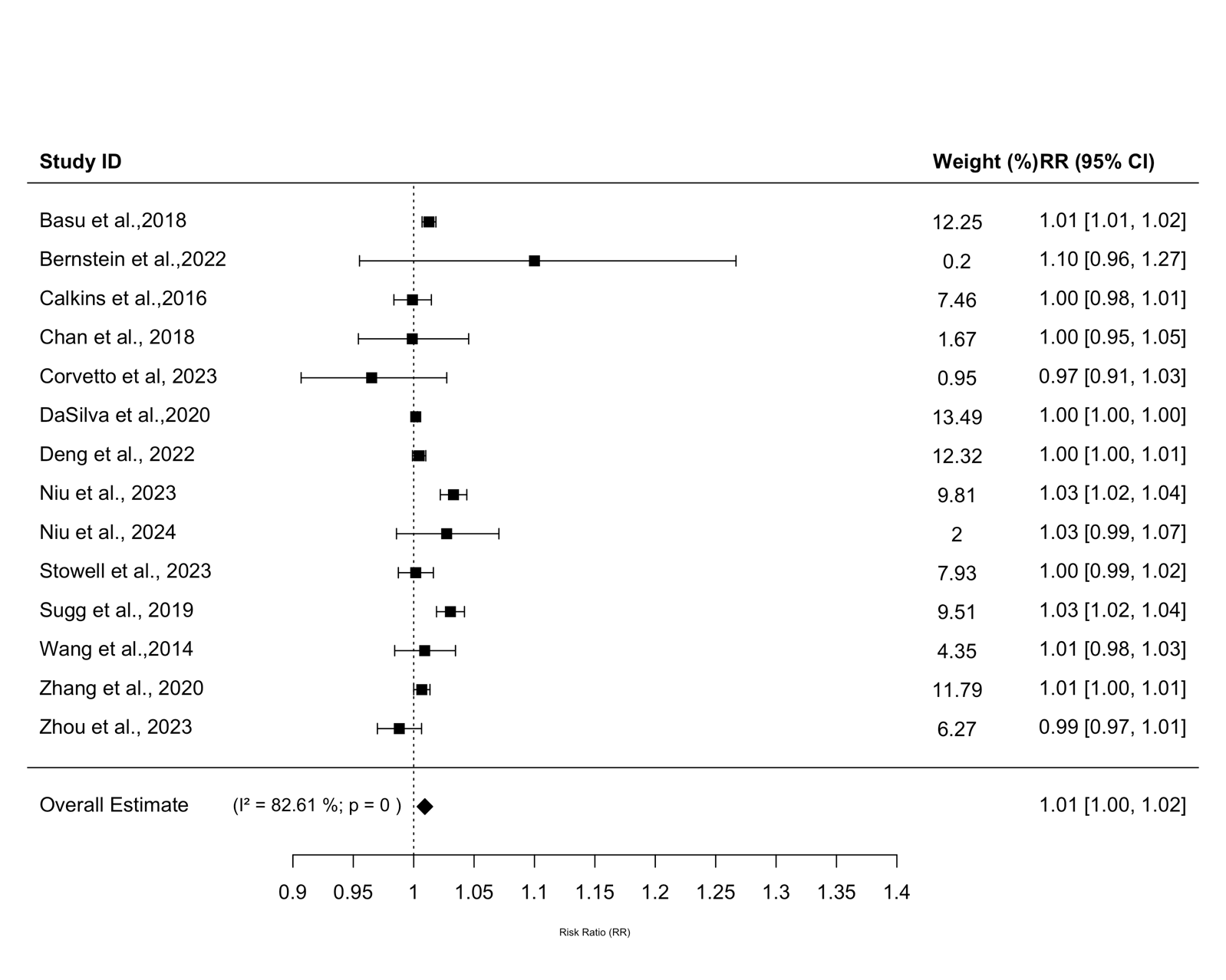


**Subgroup analysis with respect to climate zones**

**Figure H2.** Forest plot for climate 1 analysis: humid subtropical climate (Cfa).


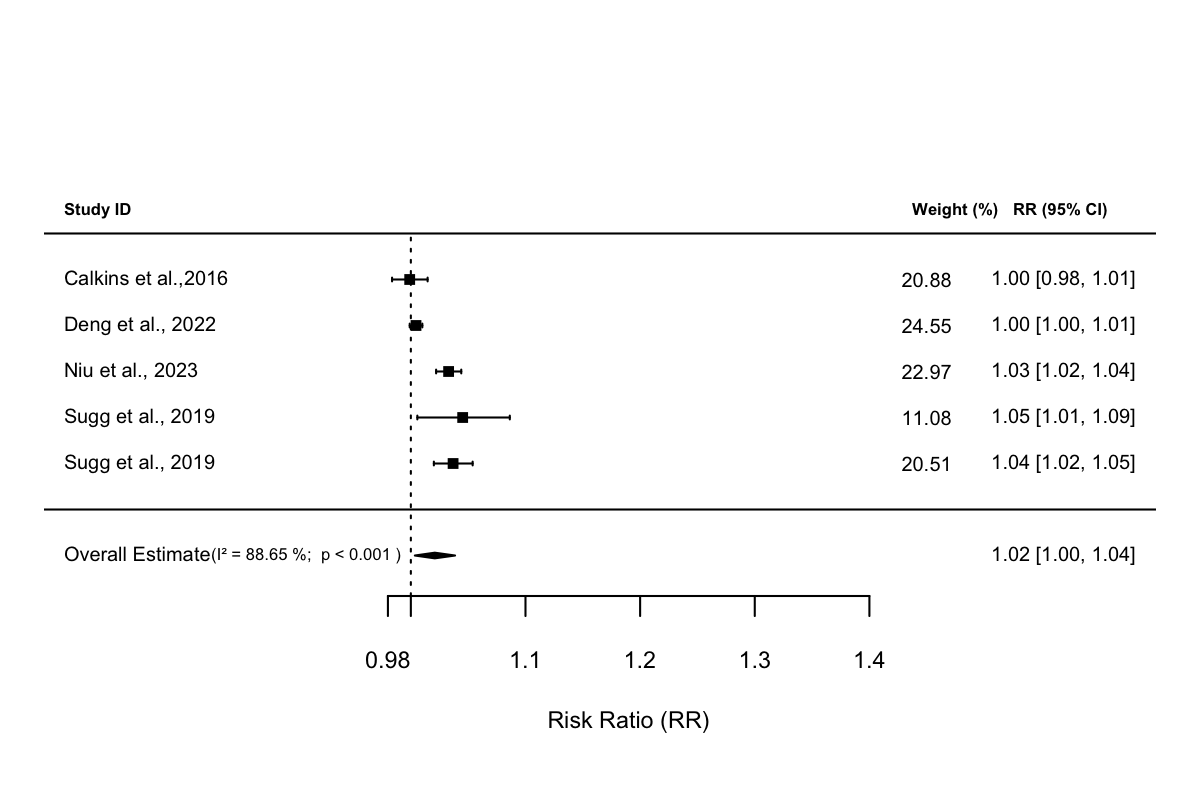


**Figure H3.** Forest Plot for climate 2 analysis: temperate oceanic climate (Cfb).


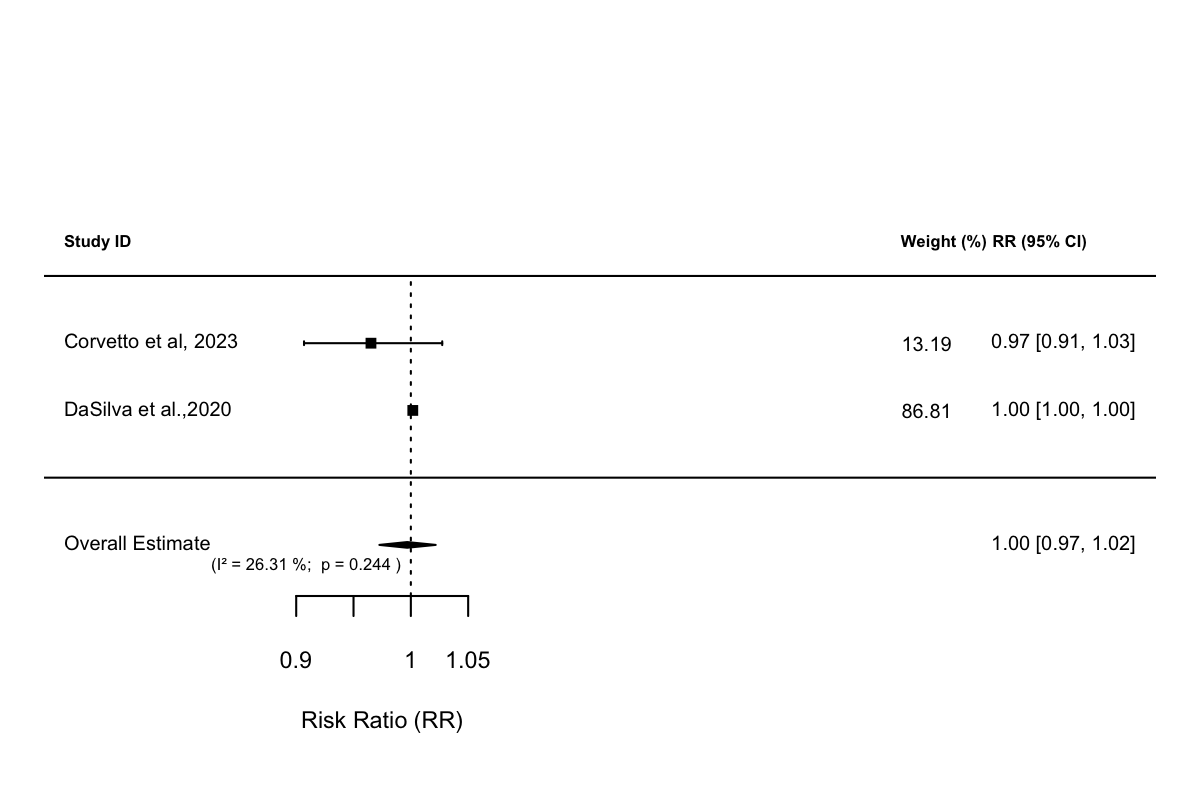


**Figure H4.** Forest Plot for climate 4 analysis: monsoon-influenced humid subtropical climate (Cwa).


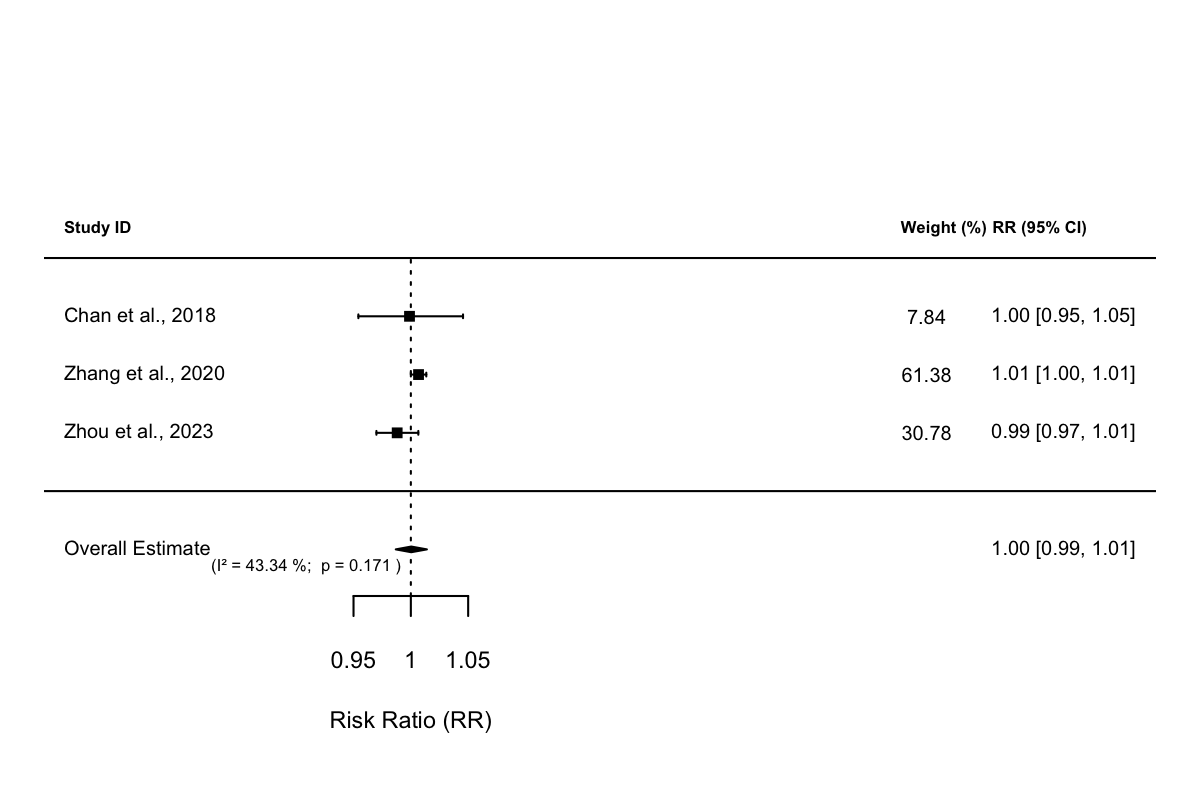


**Figure H5.** Forest plot for climate 5: hot-summer humid continental climate (Dfa).


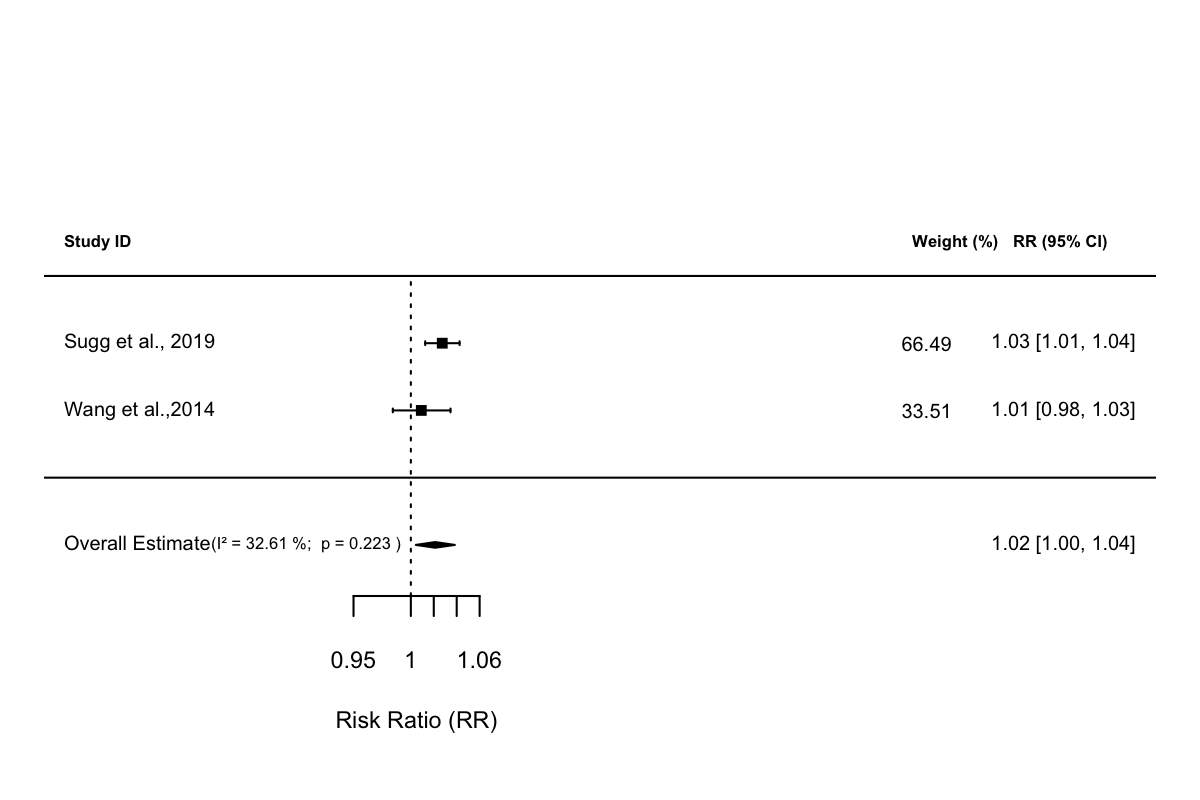


**Subgroup analysis with respect to ages**

**Figure H6.** Forest plot for age group 1 (age group 0–5).


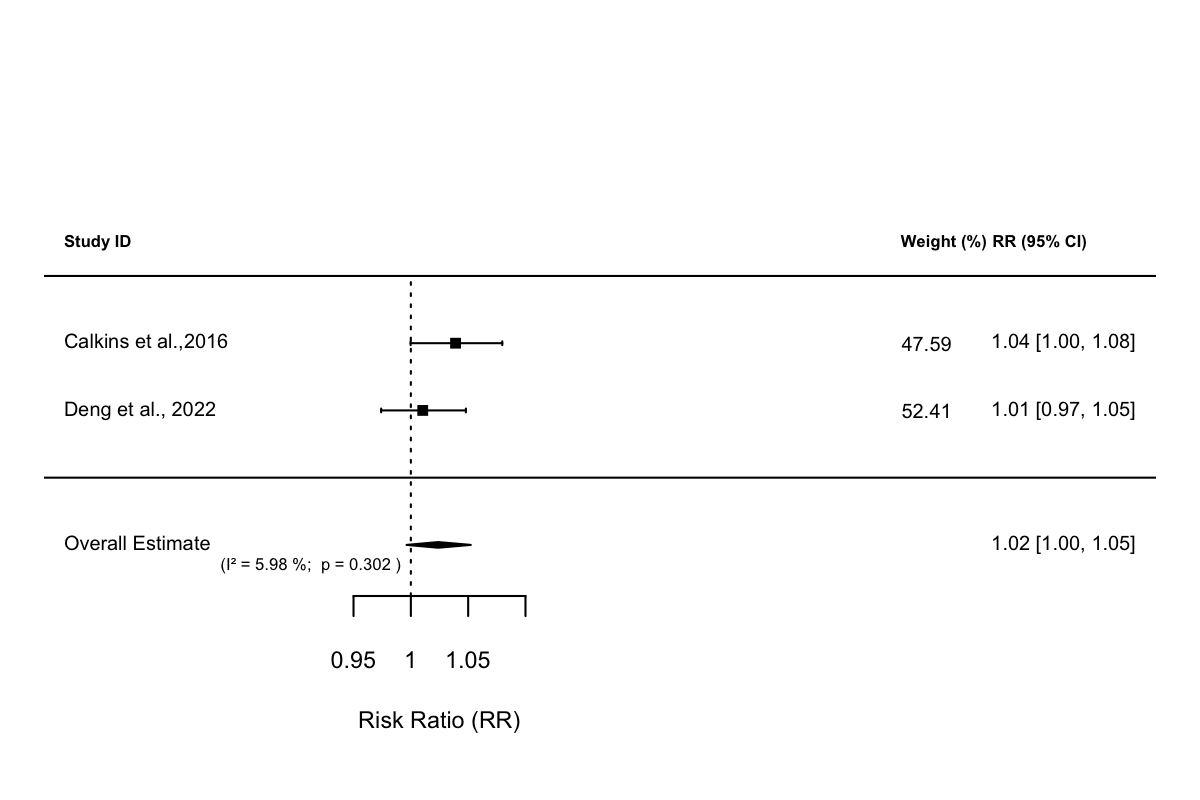


**Figure H7.** Forest plot for age group 2 (age group: 5–18).


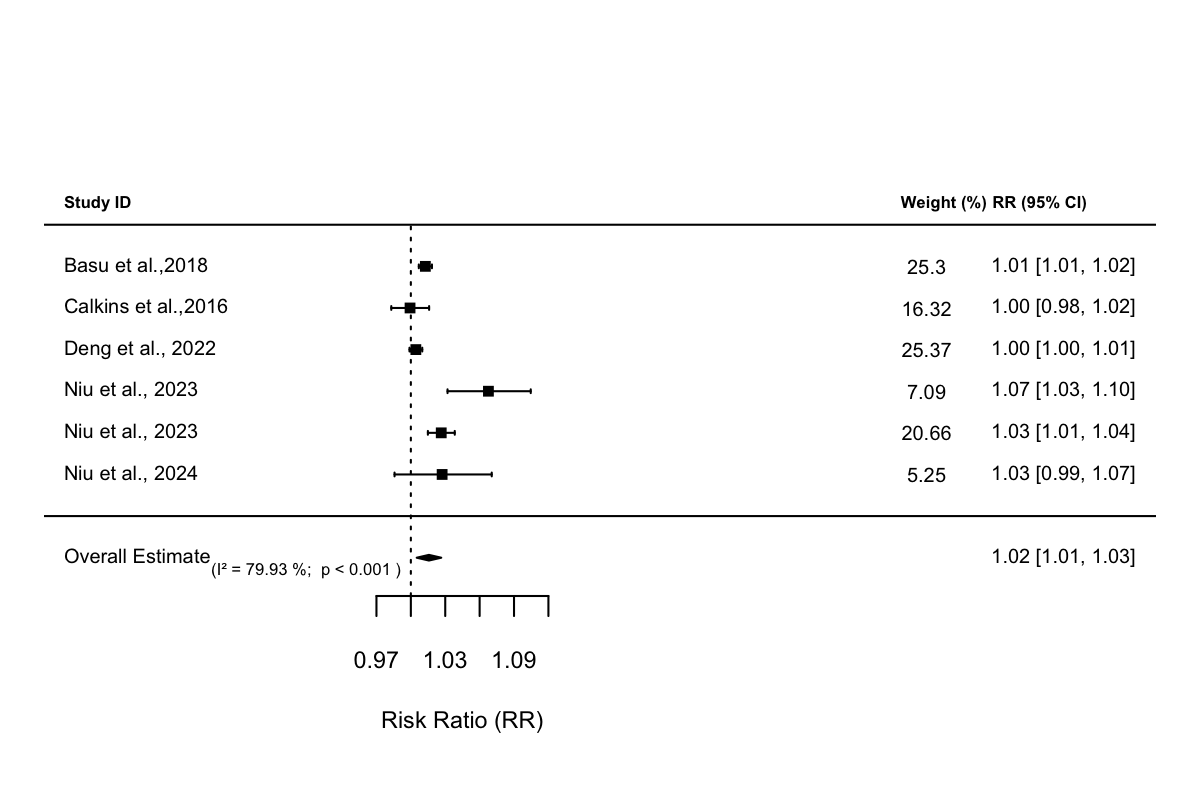


**Figure H8.** Forest plot for age group 3 (age group: ≥ 18 years).


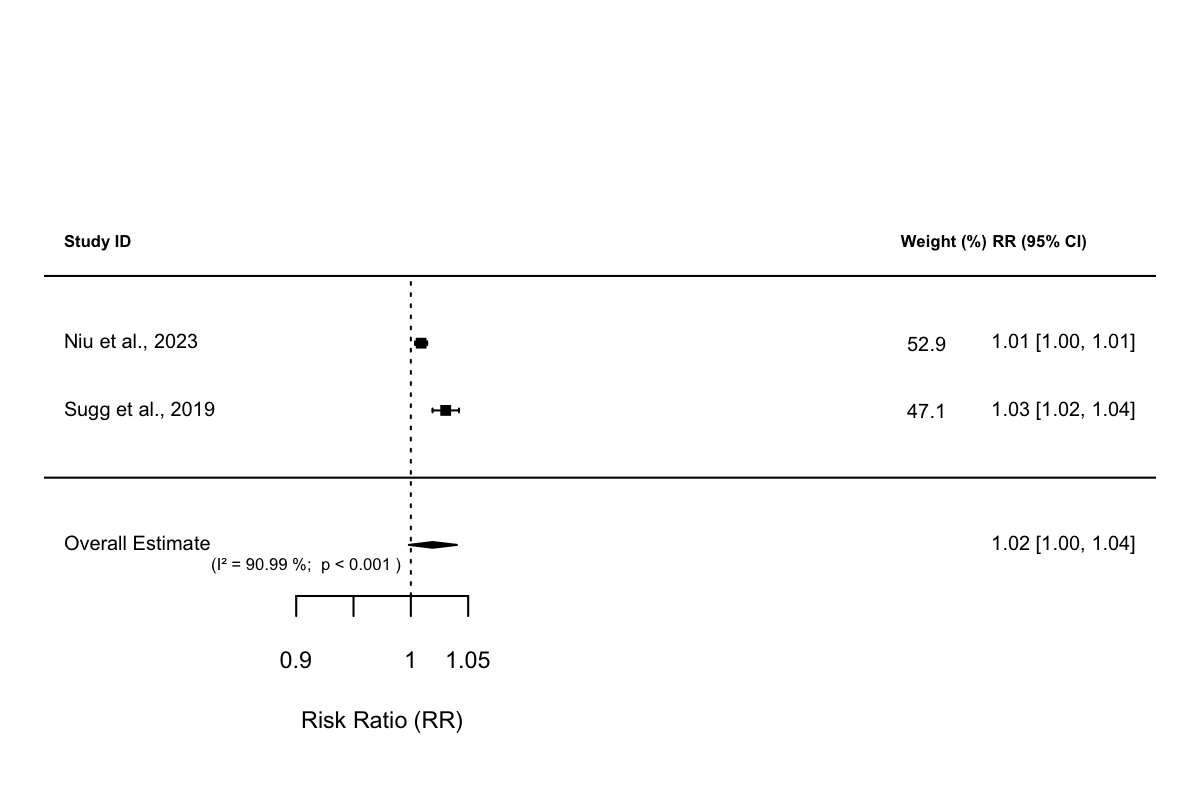


**Subgroup analysis with respect to income levels**

**Figure H9.** Forest plot for high income countries based on socio-demographic index ranking.


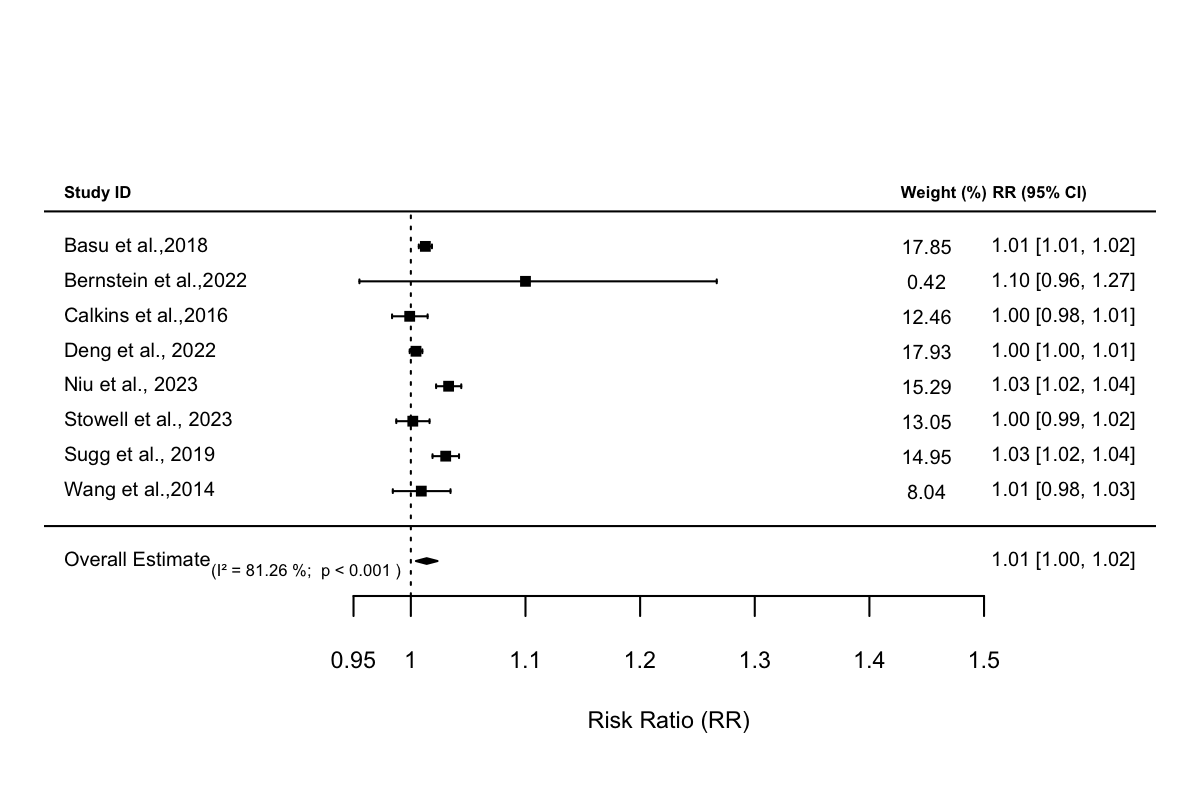


**Figure H10.** Forest plot for low-income countries based on socio-demographic index ranking.


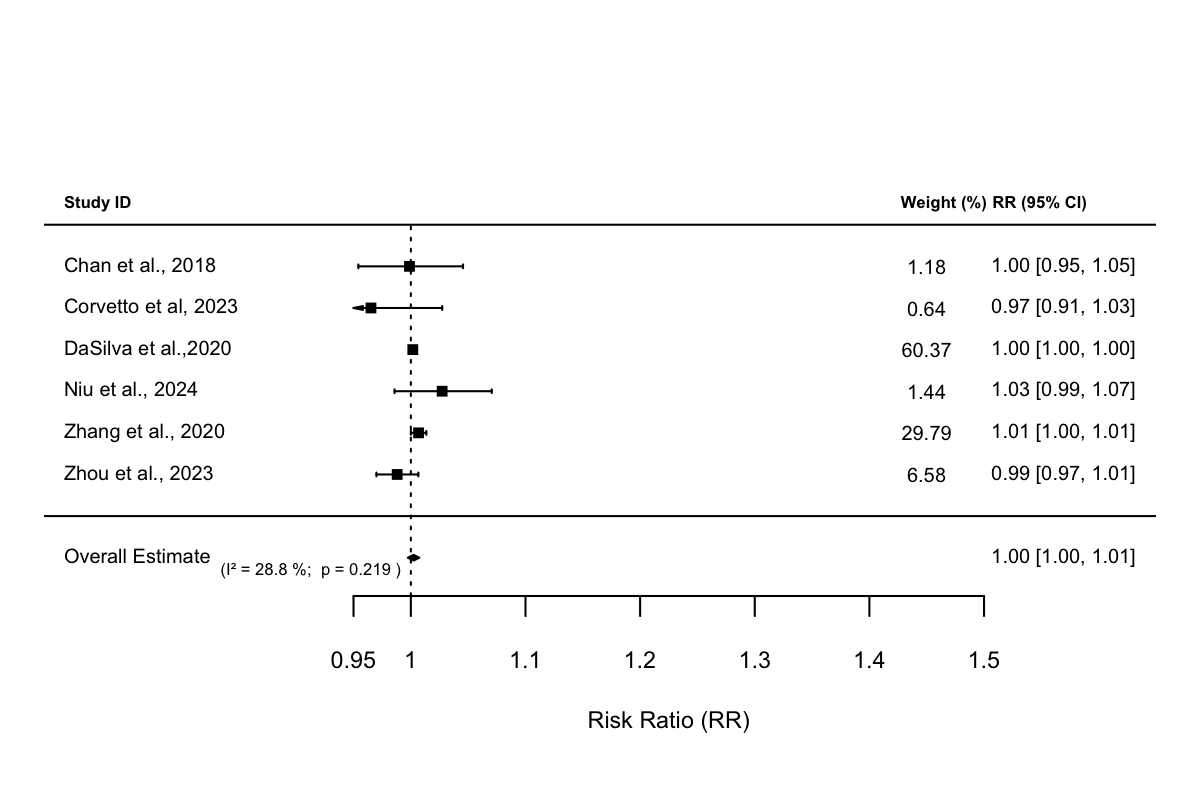


**Figure H11.** Forest plot for high exposure countries based on summary exposure value ranking.


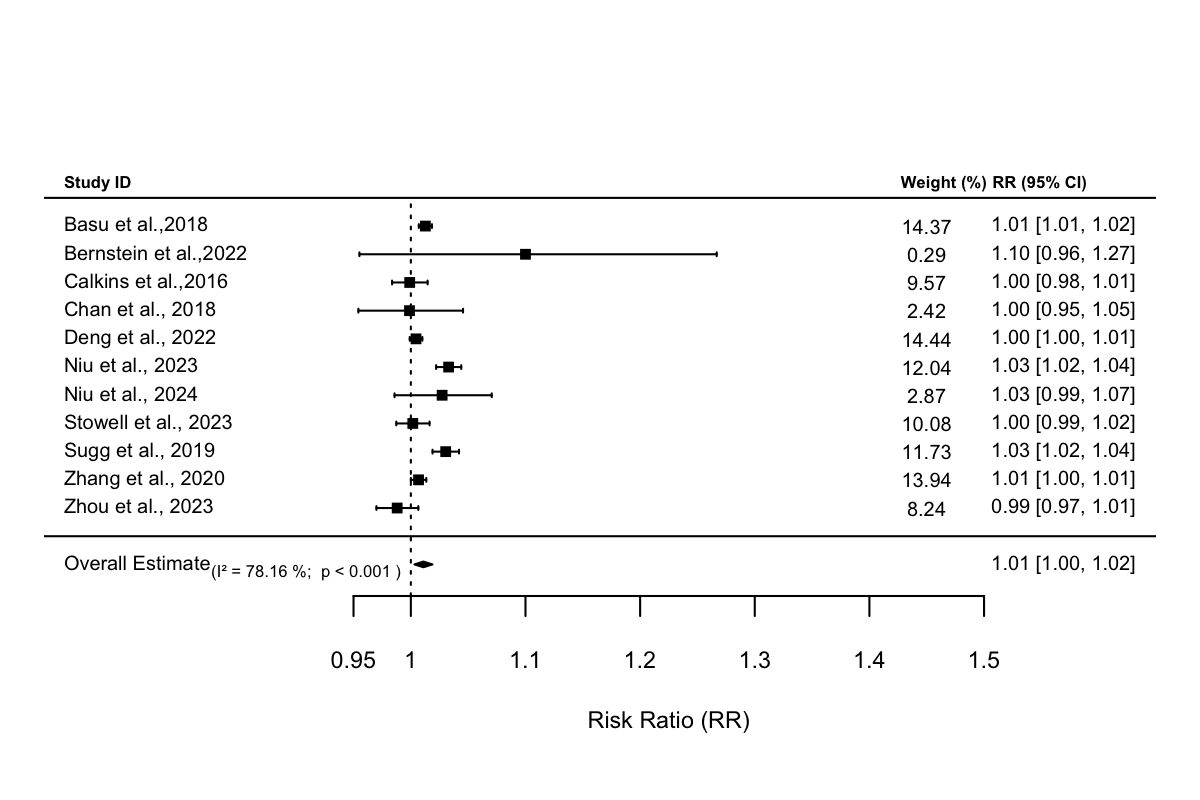


**Figure H12.** Forest plot for low exposure countries based on summary exposure value ranking.


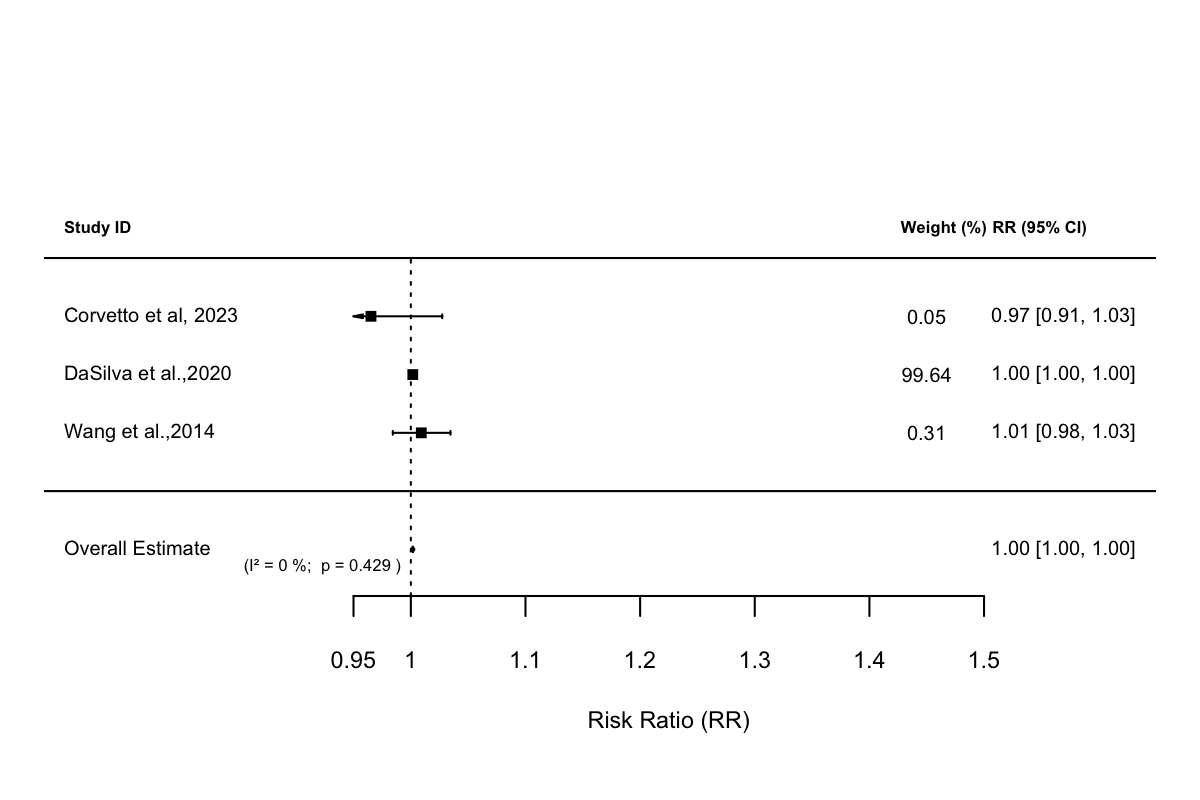


**Figure H13.** Forest plot of pooled risk estimates for studies restricted to the warm season.


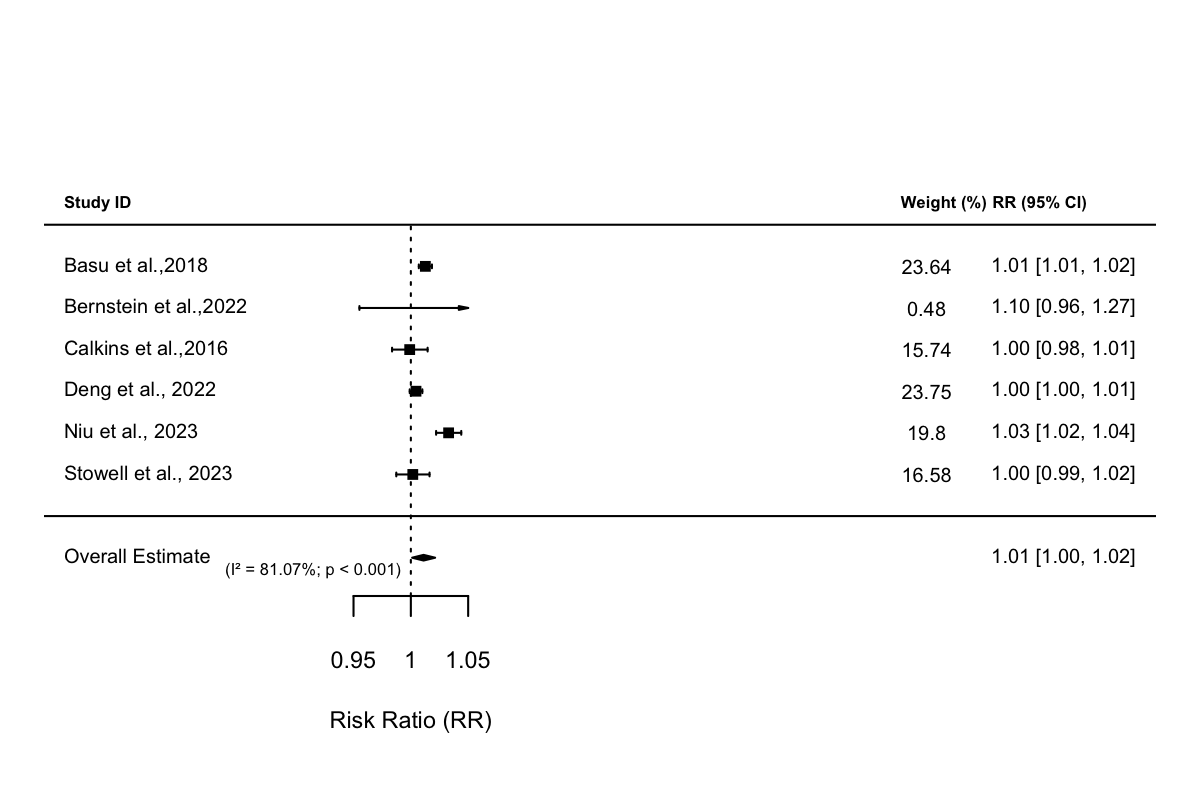


**Figure H14.** Forest plot of pooled risk estimates for studies using year-round data.


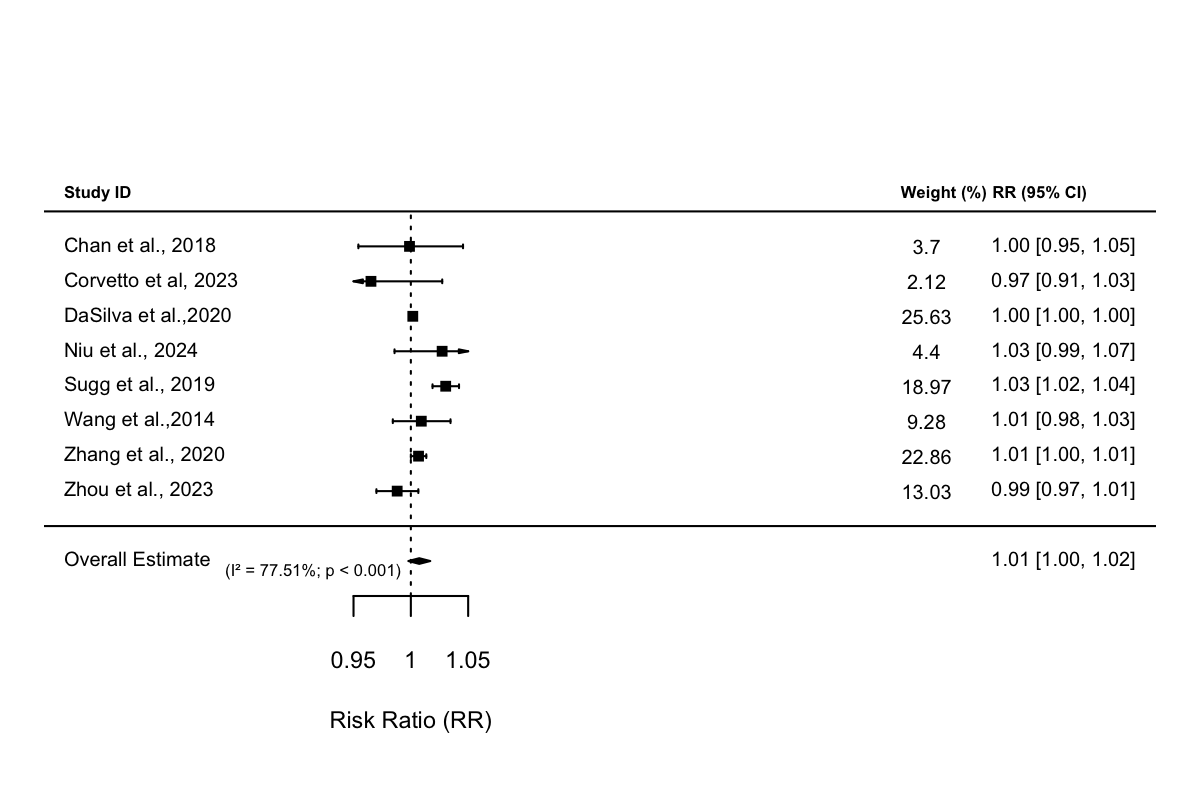


**Figure H15.** Leave-one-out analysis: overall estimate and 95% confidence interval by excluding each study. RR = relative risk


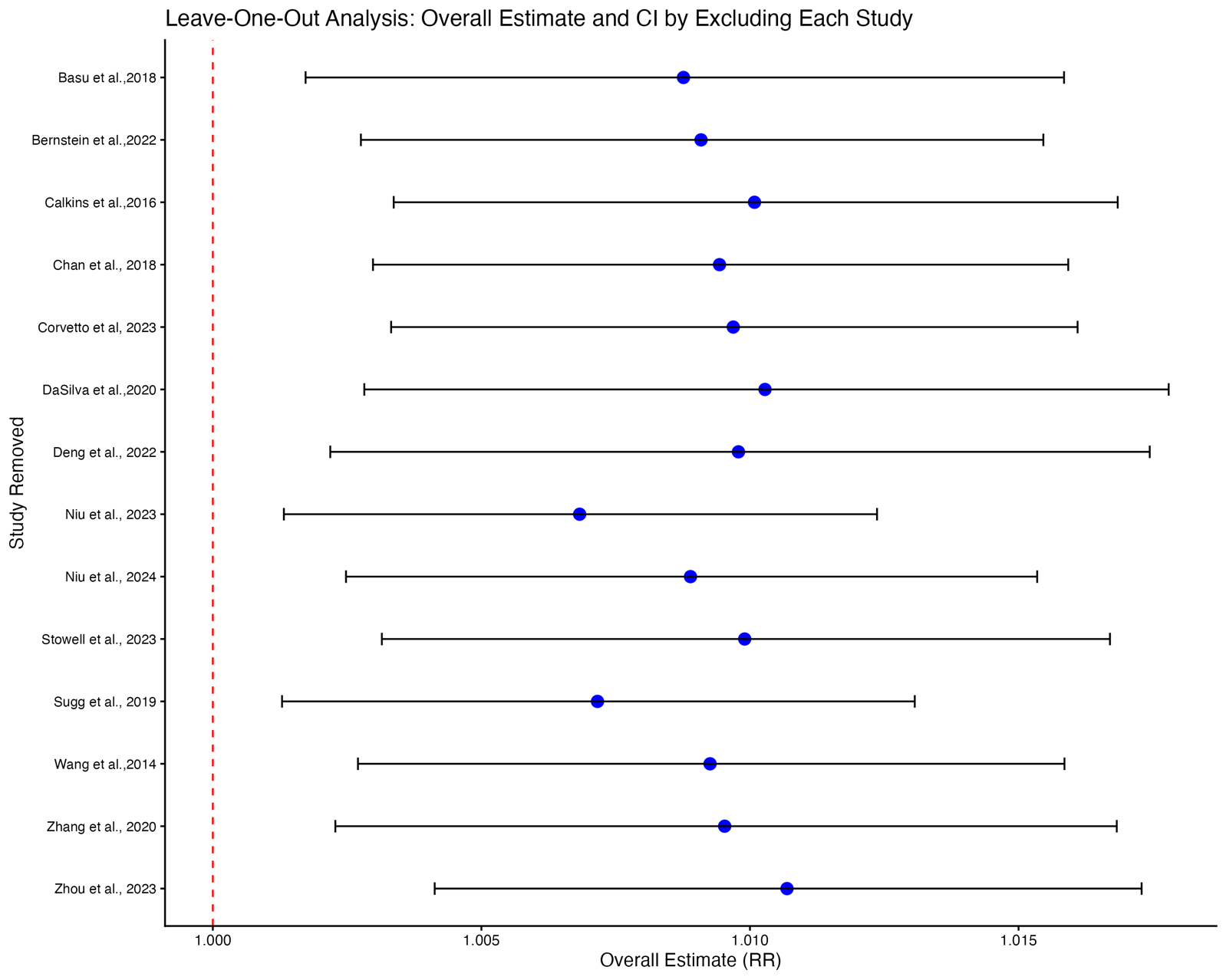


**Figure H16.** Funnel plot for Egger’s test.


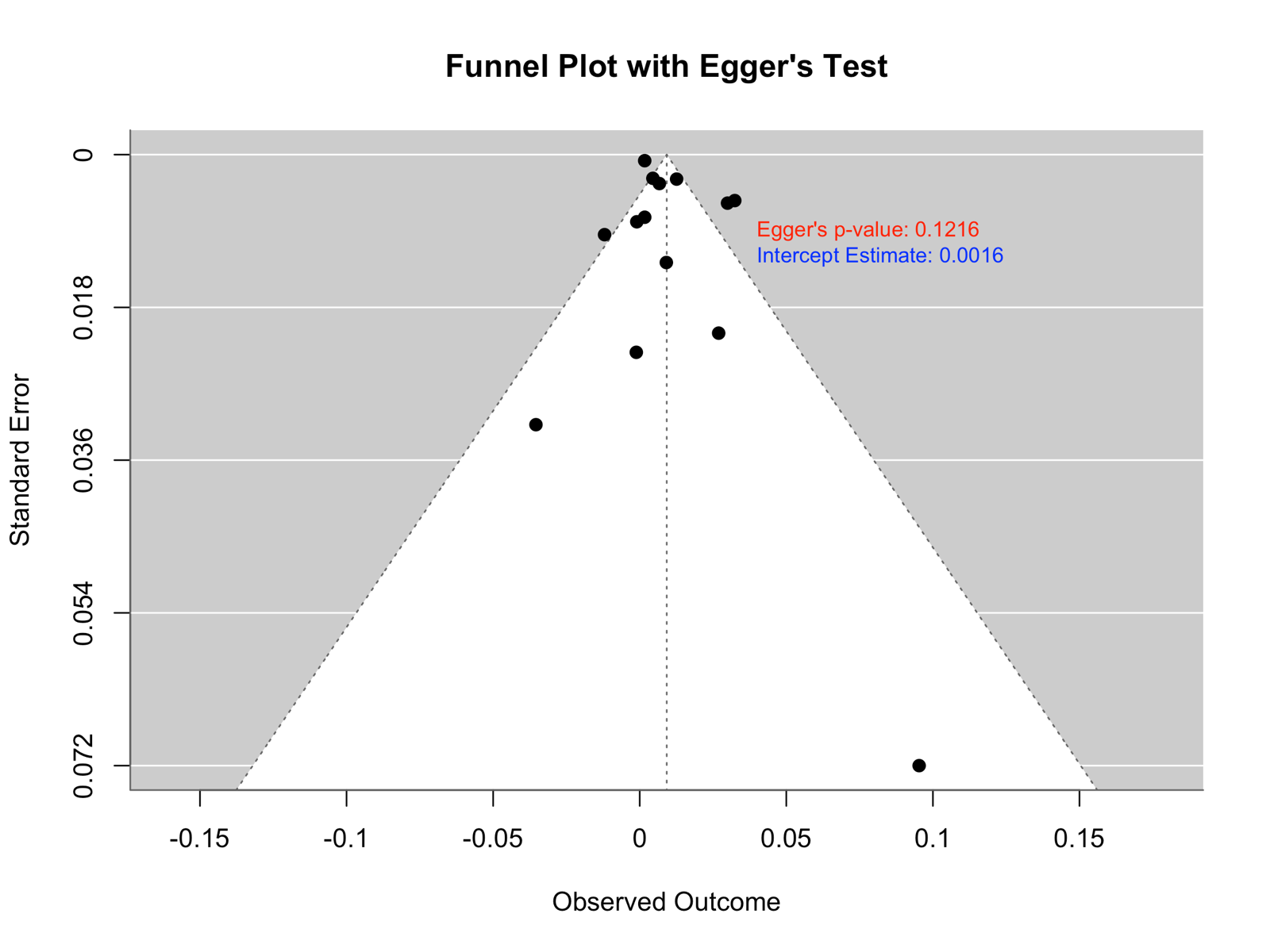


**Figure H17.** Funnel plot for trim and fill test.


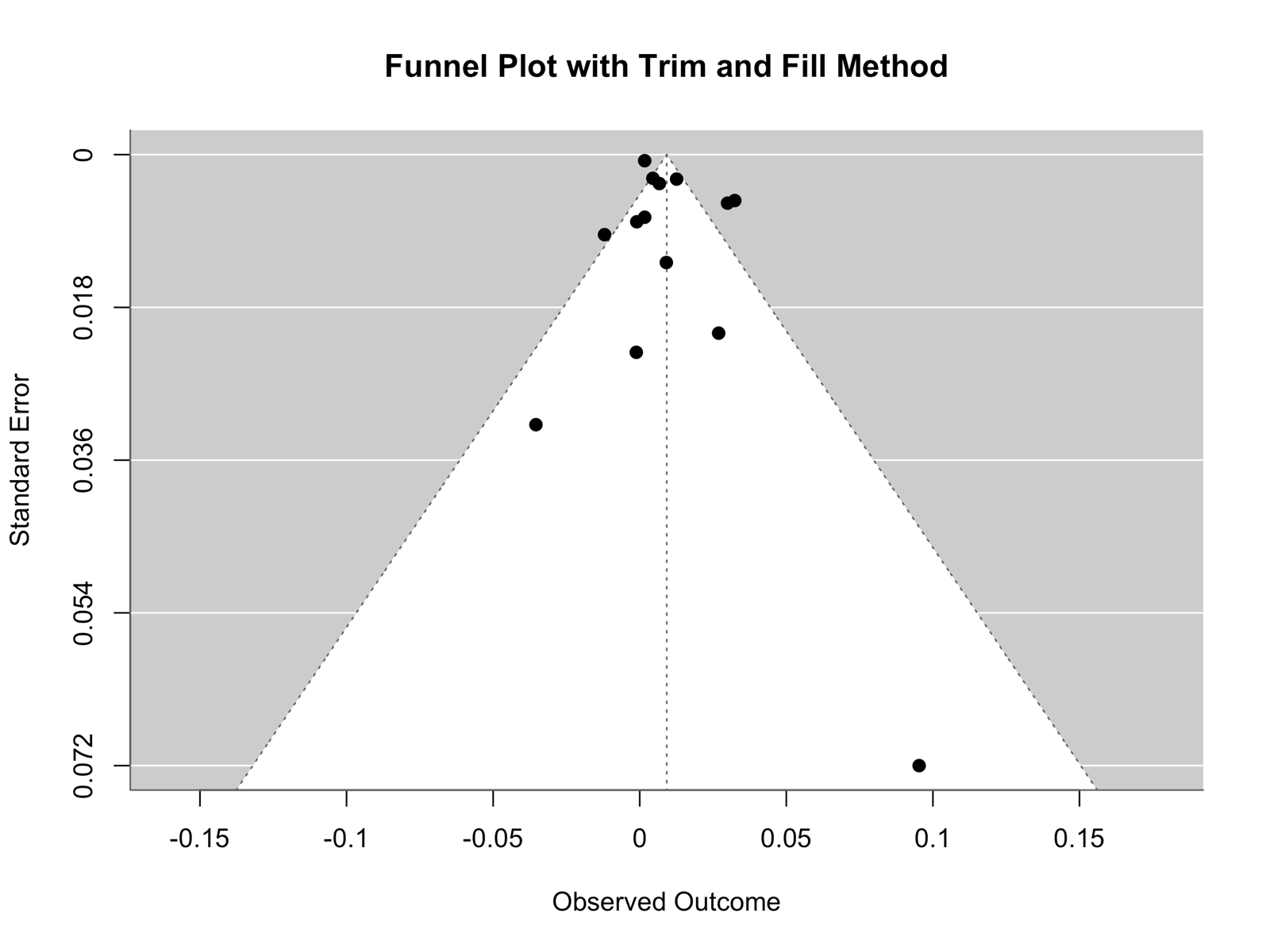


**Figure H18.** Forest plot for meta-analytical results of temperature studies including one extra study that partly fills the inclusion criteria (*n* = 15).


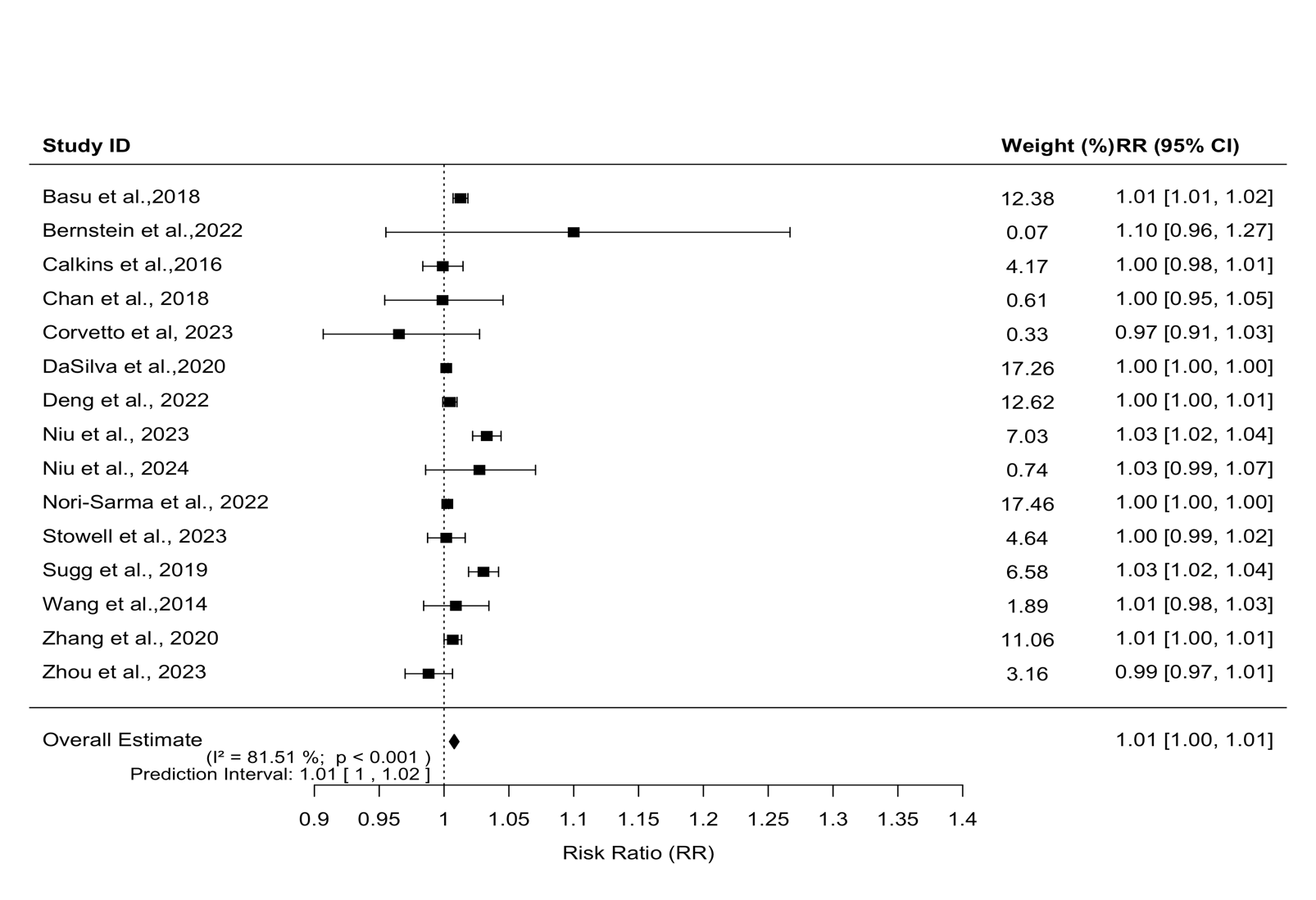


**Section I – Results for heatwave studies**

**Figure I1.** Forest plot of meta-analytical results.


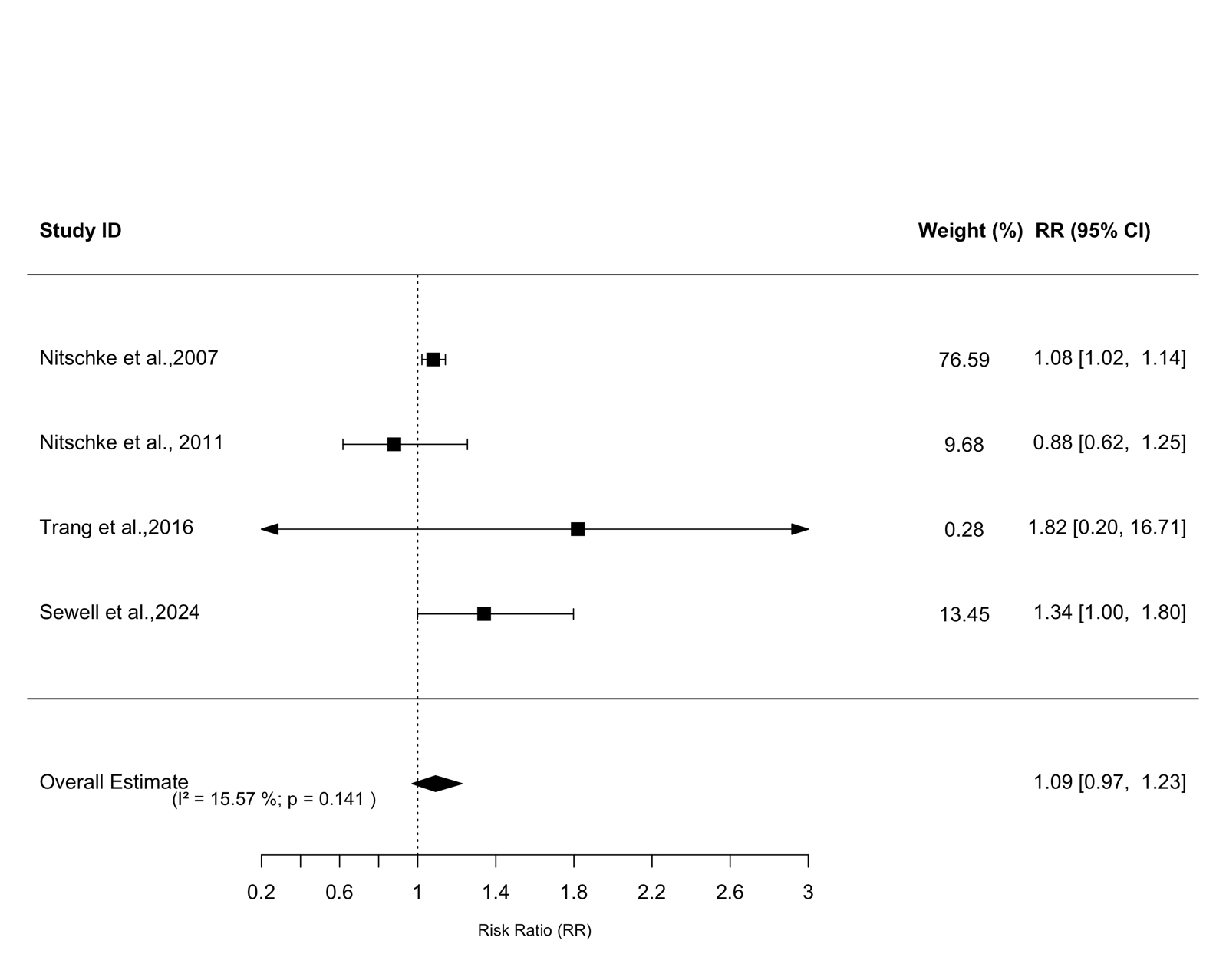


**Subgroup analysis with respect to age groups**

**Figure I2.** Forest plot for age group 1 (age group: 0–5)


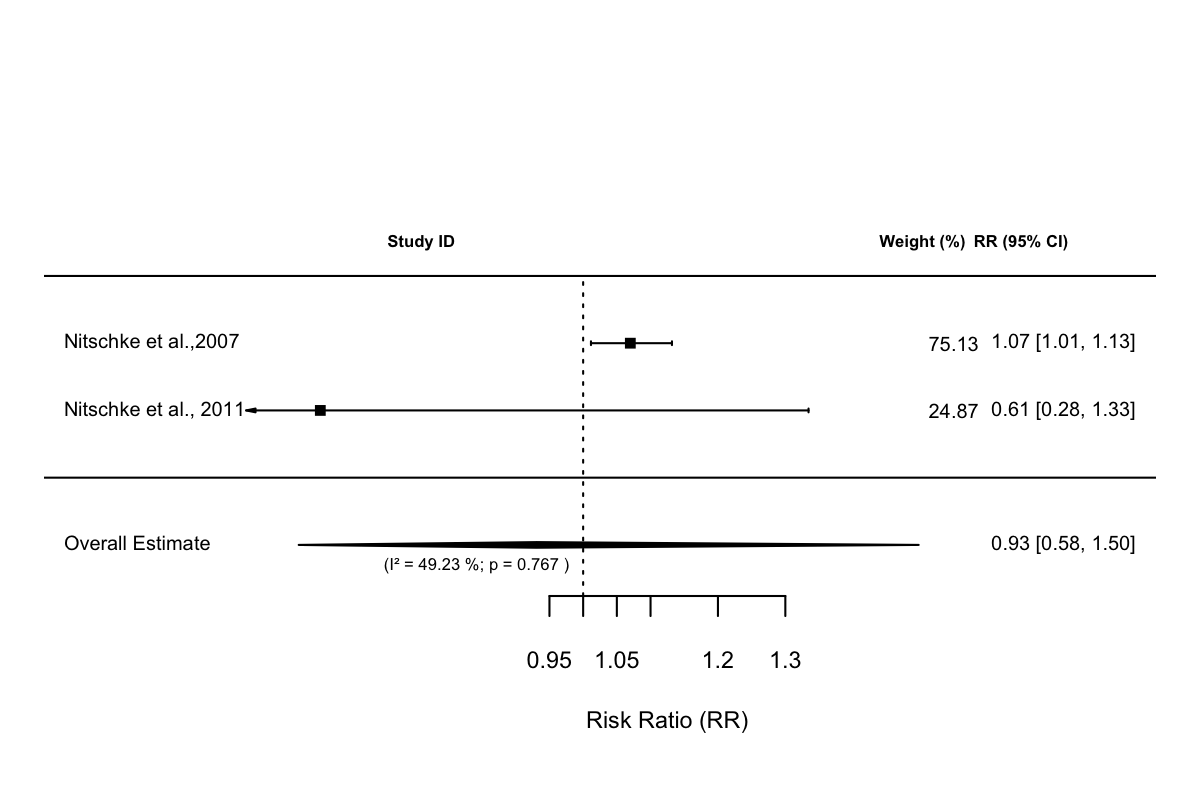


**Figure I3.** Forest plot for age group 2 (age group: 5–18)

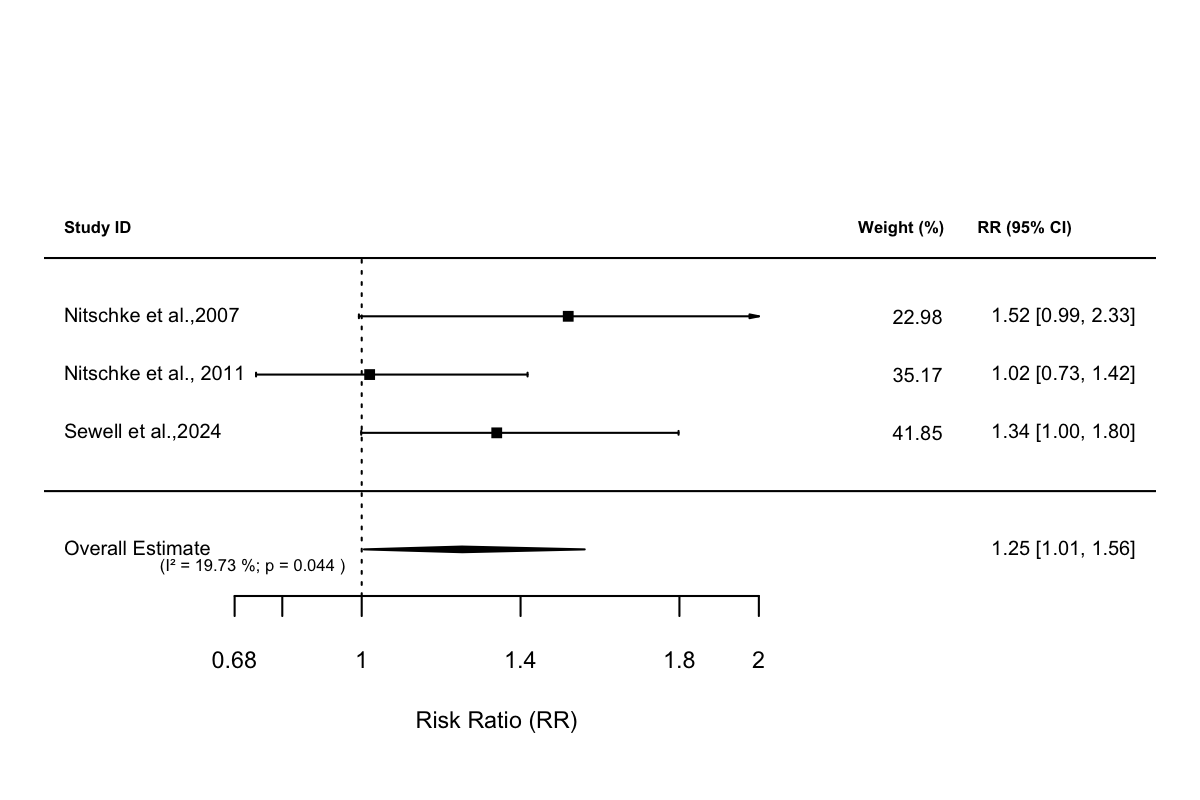


**Figure I4.** Forest plot for high income countries based on socio-demographic index ranking.


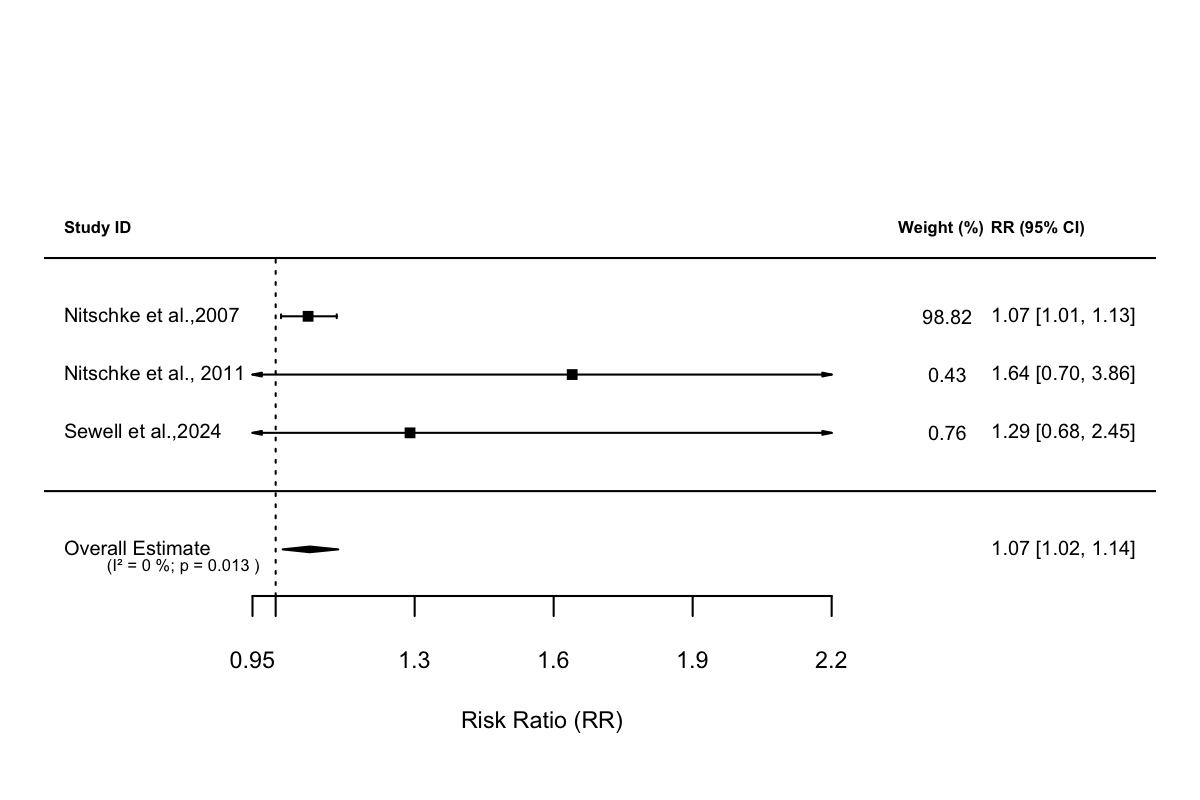


**Figure I5.** Forest plot for high exposure countries based on summary exposure value ranking.


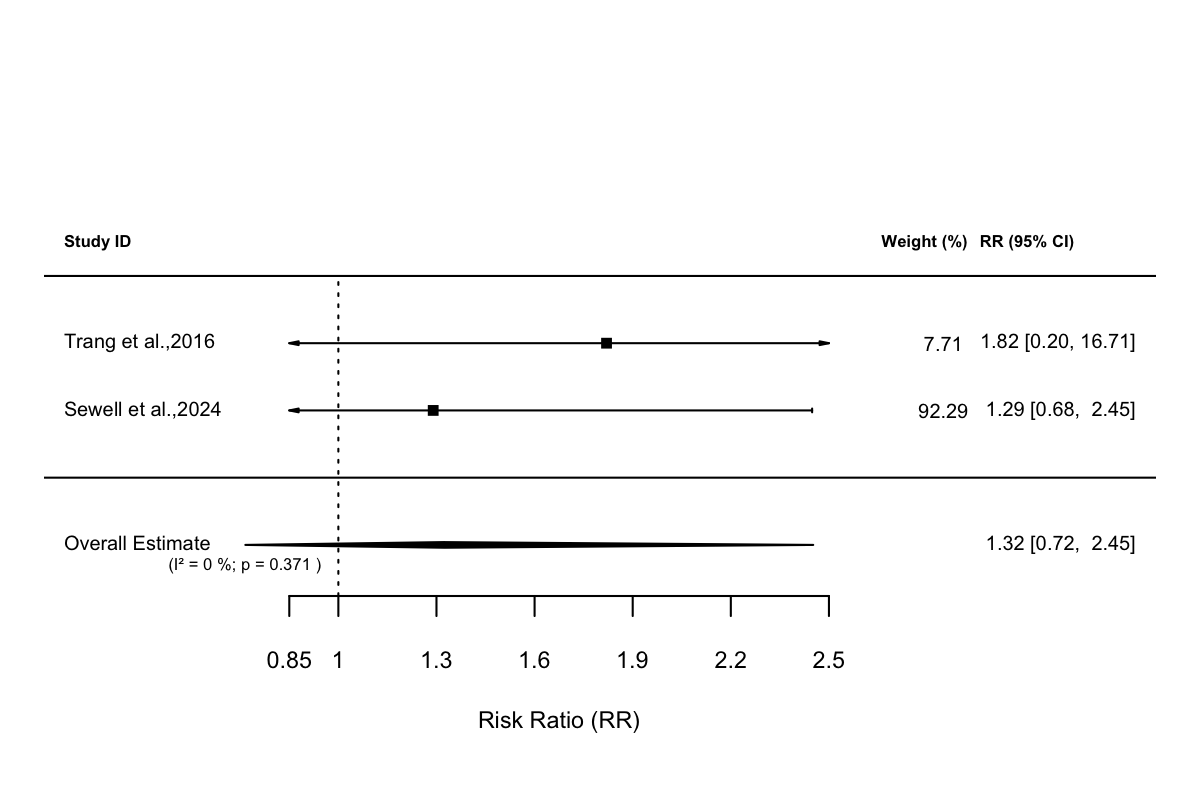


**Figure I6.** Forest plot for low exposure countries based on summary exposure value ranking.


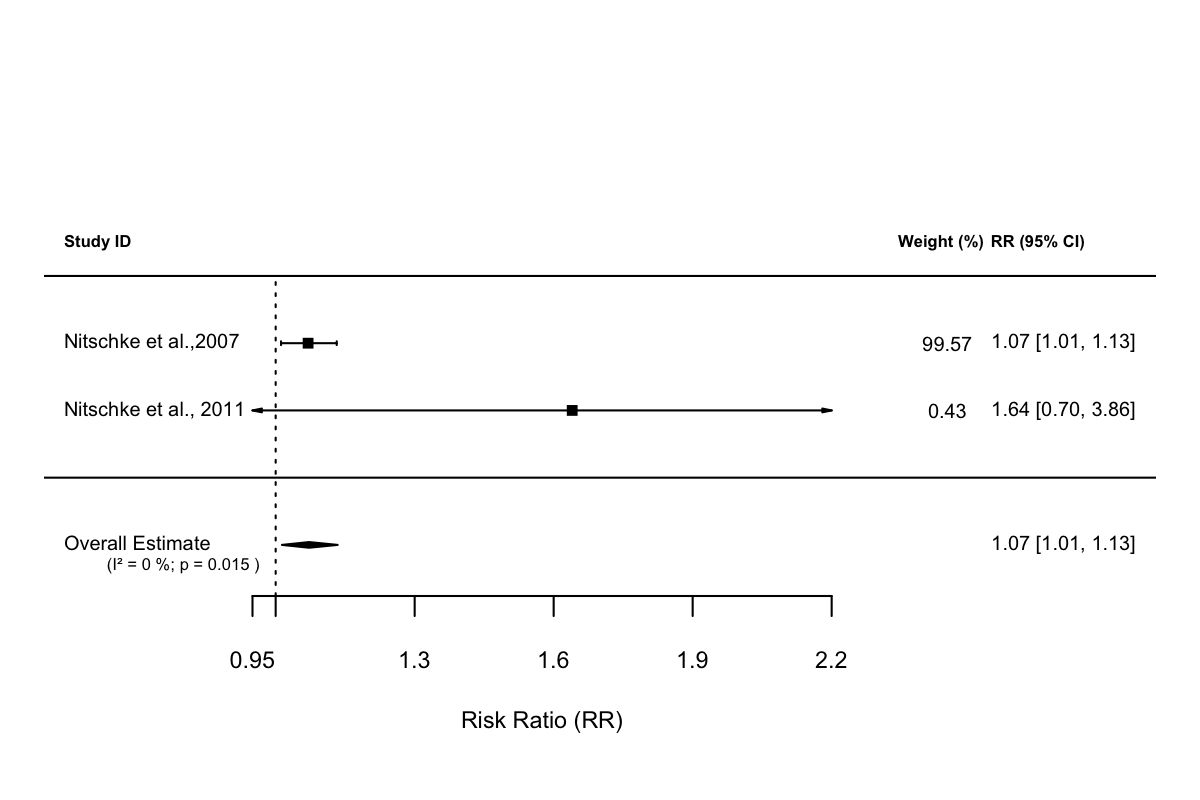


**Figure I7.** Leave-one-out analysis.


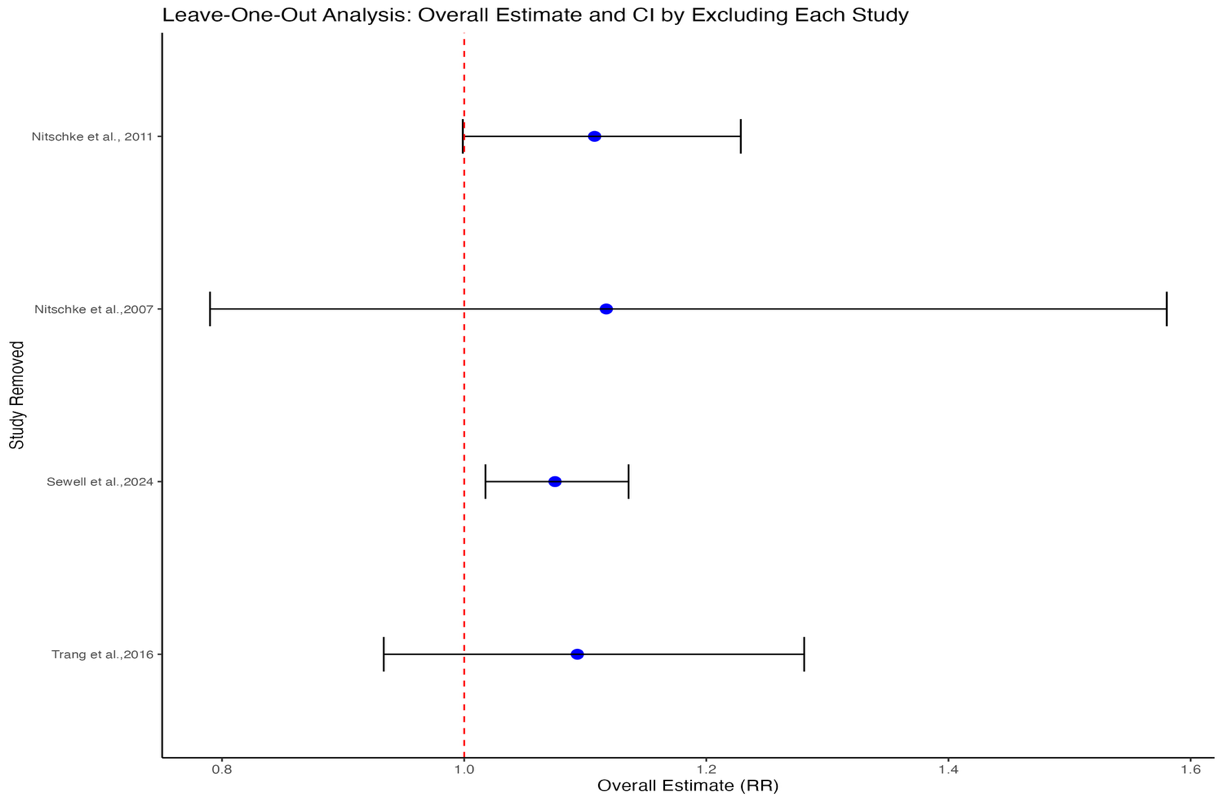


**Figure I8.** Funnel plot for Egger’s test.


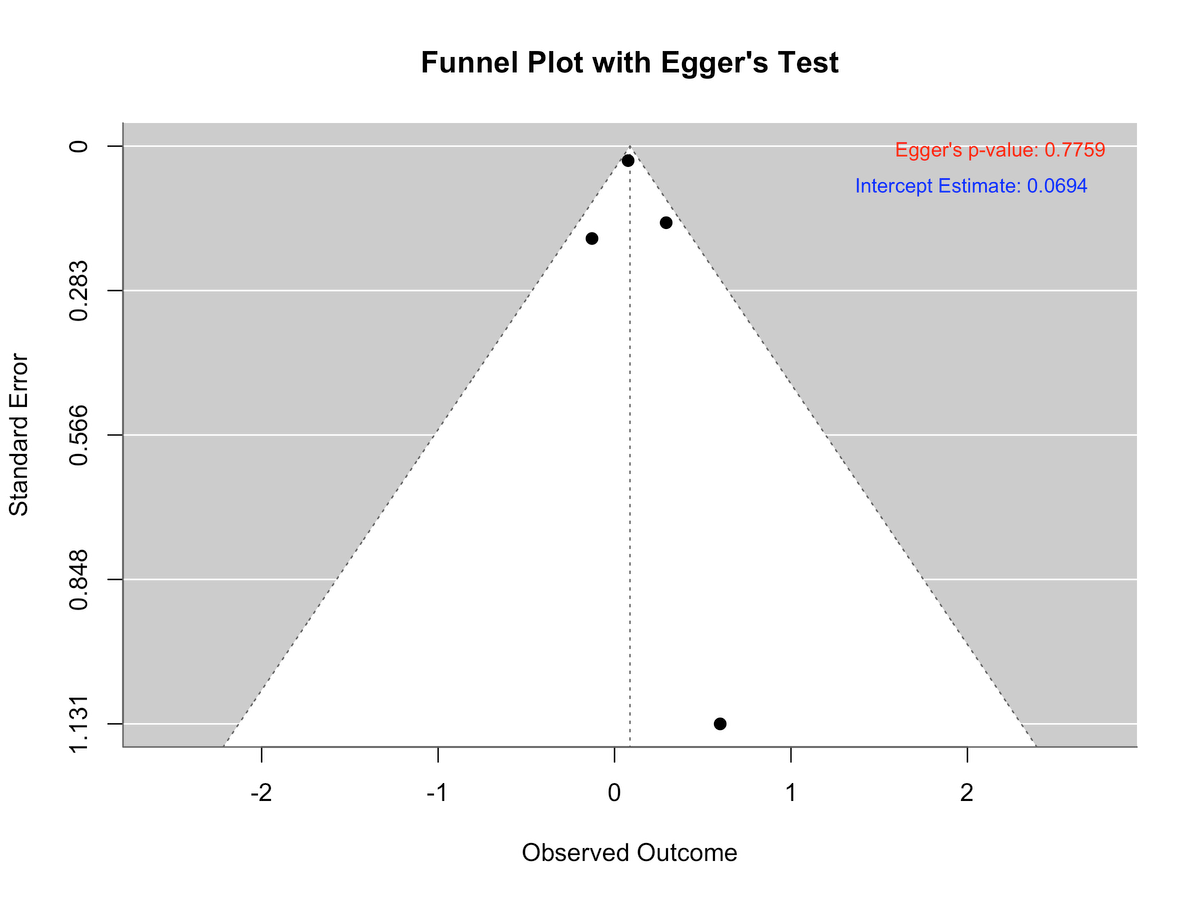


**Figure I9.** Funnel plot for trim and fill test.

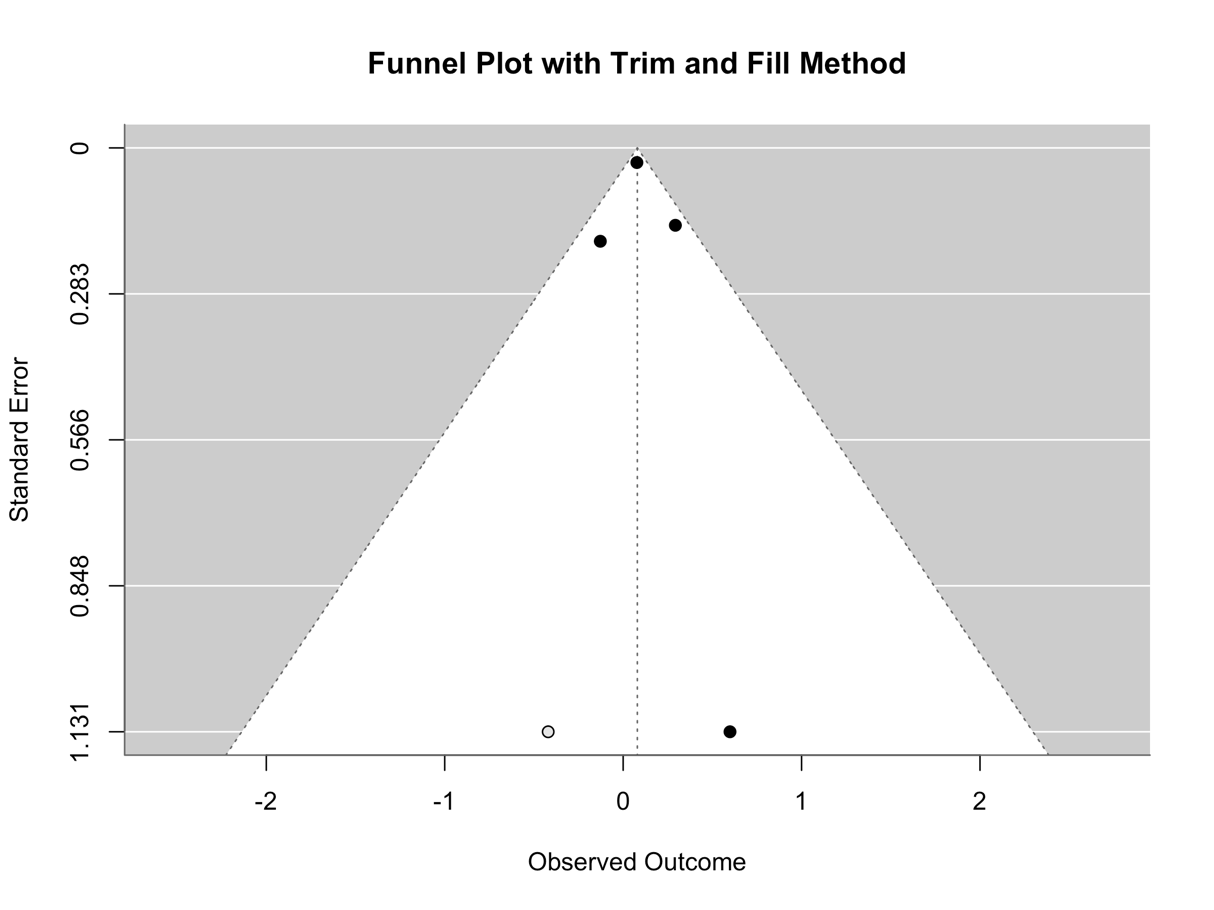


**Section J – Quality of evidence across high temperature and heatwave studies**

## **Table J1.** Quality of evidence in studies investigating the association between heat exposure and mental health outcomes**.**

| **exposure** | **risk of bias** | **indirectness** | **inconsistency** | **imprecision** | **publication bias** | **large effect** | **dose-response** | **Confounding minimises effect** | **overall quality** |
| --- | --- | --- | --- | --- | --- | --- | --- | --- | --- |
| high temperature | 0: most studies low or probably low risk of bias; no substantial difference across studies | 0: direct measures of exposure and mental health outcomes used in most studies | -1: high heterogeneity across studies (*I*² = 80.11%) | 0: 95% confidence intervals sufficiently narrow & did not include null | 0: Egger’s test did not indicate publication bias (p = 0.0957) | 0: no evidence of large magnitude effects (pooled relative risk < 2) | +1: most studies had dose-response relationship, with increase in adverse mental outcomes with high temperatures within a range | 0: confounders controlled through study design; some studies had residual confounding, but not expected to underestimated pooled estimates | moderate |
| heatwave | 0: as above | 0: as above | 0: low heterogeneity so no downgrade  (*I*² = 15.57%) | -1: 95% confidence intervals broad & include null, so downgraded | 0: as above | 0: as above | +1: as most studies had dose-response relationship, with increase in adverse mental outcomes with heatwaves within a range | 0: as above | Moderate |

**Section K - Narrative synthesis**

Five studies excluded from the meta-analysis are summarised below because their exposure metrics or exposure-outcome frameworks were not directly comparable with the pooled short-term temperature and heatwave estimates, although their findings were broadly relevant to the review question.

**Table K1.** Narrative synthesis of studies excluded from the meta-analysis

| **Study** | **Exposure** | **Outcome** | **Reason excluded from meta-analysis** | **Main finding** | **Relevance to review** |
| --- | --- | --- | --- | --- | --- |
| Cohen et al. 2024 (10) | Diurnal temperature range | Mental health-related hospital visits, including mood and schizophrenia-spectrum disorders | Exposure metric not directly comparable with short-term ambient temperature or heatwave measures used in the pooled meta-analysis | A 16% increase in hospital visits for mood disorders was reported as diurnal temperature range increased from 0.1 to 12.2 °C, with strongest effects for mood and schizophrenia-spectrum disorders in those aged 0–24 years. | Suggests that temperature variability, in addition to absolute heat exposure, may be associated with adverse mental health outcomes in children and young people. |
| Doganay et al. 2003 (13) | Temperature, sunlight, and humidity (seasonal/meteorological variation) | Suicide attempts among 15–24-year-olds | Exposure-outcome framework and design not directly comparable with pooled short-term temperature and heatwave estimates | Seasonal peaks in suicide attempts were observed and were positively correlated with temperature, sunlight, and humidity. | Supports a possible association between hotter conditions and adverse mental health outcomes, although findings were not directly comparable for quantitative pooling. |
| Younan et al. 2018 (18) | Long-term ambient temperature exposure | Aggressive/externalising behaviours in adolescents | Long-term exposure metric not comparable with short-term temperature increases or heatwave measures | Each 1 °C increase in long-term ambient temperature was associated with increased aggressive behaviours in adolescents, equivalent to a 1.5–3-year delay in behavioural maturation, particularly among girls and socio-economically disadvantaged youth. | Suggests that sustained heat exposure may influence behavioural and mental health development in adolescents. |
| Aydin-Ghormoz et al. 2022 (5) | Concurrent temperature-related illness / heat-related illness context | Behavioural health disorder hospitalizations with concurrent heat-related illness | Exposure-outcome definition not directly comparable with pooled ambient temperature and heatwave measures | Schizophrenia was associated with an increased risk of concurrent heat-related illness among behavioural health disorder hospitalizations (RR 1.38, 95% CI: 1.19–1.60). | Indicates that severe psychiatric presentations may be vulnerable during heat-related illness episodes, but the framework differed from other included studies. |
| Komulainen et al. 2022 (14) | Childhood ambient temperature exposure (long-term) | Risk of schizophrenia from childhood to early adulthood | Long-term exposure metric not comparable with pooled short-term temperature and heatwave measures | Higher childhood ambient temperature exposure was associated with greater schizophrenia risk in partially adjusted models, but the association was not consistent after full adjustment. | Provides tentative evidence for a possible long-term association, but findings were not robust after full adjustment. |

1. Deng X, Brotzge J, Tracy M, Chang HH, Romeiko X, Zhang W, et al. Identifying joint impacts of sun radiation, temperature, humidity, and rain duration on triggering mental disorders using a high-resolution weather monitoring system. Environment International. 2022;167:107411.

2. Niu L, Girma B, Liu B, Schinasi LH, Clougherty JE, Sheffield P. Temperature and mental health–related emergency department and hospital encounters among children, adolescents and young adults. Epidemiology and psychiatric sciences. 2023;32:e22.

3. Wang X, Lavigne E, Ouellette-kuntz H, Chen BE. Acute impacts of extreme temperature exposure on emergency room admissions related to mental and behavior disorders in Toronto, Canada. Journal of affective disorders. 2014;155:154-61.

4. Xie CX, Machado GC. Clinimetrics: Grading of recommendations, assessment, development and evaluation (GRADE). Journal of physiotherapy. 2021;67(1):66.

5. Aydin-Ghormoz H, Adeyeye T, Muscatiello N, Nayak S, Savadatti S, Insaf TZ. Identifying risk factors for hospitalization with behavioral health disorders and concurrent temperature-related illness in New York State. International journal of environmental research and public health. 2022;19(24):16411.

6. Basu R, Gavin L, Pearson D, Ebisu K, Malig B. Examining the association between apparent temperature and mental health-related emergency room visits in California. American journal of epidemiology. 2018;187(4):726-35.

7. Bernstein AS, Sun S, Weinberger KR, Spangler KR, Sheffield PE, Wellenius GA. Warm season and emergency department visits to US children’s hospitals. Environmental health perspectives. 2022;130(1):017001.

8. Calkins MM, Isaksen TB, Stubbs BA, Yost MG, Fenske RA. Impacts of extreme heat on emergency medical service calls in King County, Washington, 2007–2012: relative risk and time series analyses of basic and advanced life support. Environmental health. 2016;15:1-13.

9. Chan EY, Lam HC, So SH, Goggins III WB, Ho JY, Liu S, et al. Association between ambient temperatures and mental disorder hospitalizations in a subtropical city: a time-series study of Hong Kong special administrative region. International journal of environmental research and public health. 2018;15(4):754.

10. Cohen G, Rowland ST, Benavides J, Lindert J, Kioumourtzoglou M-A, Parks RM. Daily temperature variability and mental health-related hospital visits in New York State. Environmental Research. 2024;257:119238.

11. Corvetto JF, Federspiel A, Sewe MO, Müller T, Bunker A, Sauerborn R. Impact of heat on mental health emergency visits: a time series study from all public emergency centres, in Curitiba, Brazil. BMJ open. 2023;13(12):e079049.

12. da Silva I, de Almeida DS, Hashimoto EM, Martins LD. Risk assessment of temperature and air pollutants on hospitalizations for mental and behavioral disorders in Curitiba, Brazil. Environmental health. 2020;19:1-11.

13. Doganay Z, Sunter AT, Guz H, Ozkan A, Altintop L, Kati C, et al. Climatic and diurnal variation in suicide attempts in the ED. The American Journal of emergency medicine. 2003;21(4):271-5.

14. Komulainen K, Elovainio M, Törmälehto S, Ruuhela R, Sund R, Partonen T, et al. Climatic exposures in childhood and the risk of schizophrenia from childhood to early adulthood. Schizophrenia Research. 2022;248:233-9.

15. Niu Y-L, Lu F, Liu X-J, Wang J, Li Liu D, Liu Q-Y, et al. Global climate change: Effects of future temperatures on emergency department visits for mental disorders in Beijing, China. Environmental Research. 2024;252:119044.

16. Stowell JD, Sun Y, Spangler KR, Milando CW, Bernstein A, Weinberger KR, et al. Warm-season temperatures and emergency department visits among children with health insurance. Environmental Research: Health. 2022;1(1):015002.

17. Sugg MM, Dixon PG, Runkle JD. Crisis support-seeking behavior and temperature in the United States: is there an association in young adults and adolescents? Science of the Total Environment. 2019;669:400-11.

18. Younan D, Li L, Tuvblad C, Wu J, Lurmann F, Franklin M, et al. Long-term ambient temperature and externalizing behaviors in adolescents. American journal of epidemiology. 2018;187(9):1931-41.

19. Zhang S, Yang Y, Xie X, Li H, Han R, Hou J, et al. The effect of temperature on cause-specific mental disorders in three subtropical cities: a case-crossover study in China. Environment international. 2020;143:105938.

20. Zhou Y, Ji A, Tang E, Liu J, Yao C, Liu X, et al. The role of extreme high humidex in depression in chongqing, China: A time series-analysis. Environmental Research. 2023;222:115400.

21. Nitschke M, Tucker GR, Bi P. Morbidity and mortality during heatwaves in metropolitan Adelaide. Medical journal of Australia. 2007;187(11-12):662-5.

22. Nitschke M, Tucker GR, Hansen AL, Williams S, Zhang Y, Bi P. Impact of two recent extreme heat episodes on morbidity and mortality in Adelaide, South Australia: a case-series analysis. Environmental Health. 2011;10:1-9.

23. Trang PM, Rocklöv J, Giang KB, Kullgren G, Nilsson M. Heatwaves and hospital admissions for mental disorders in northern Vietnam. PloS one. 2016;11(5):e0155609.

24. Sewell K, Paul S, De Polt K, Sugg MM, Leeper RD, Rao D, et al. Impacts of compounding drought and heatwave events on child mental health: insights from a spatial clustering analysis. Discover mental health. 2024;4(1):1.
